# Genome Analysis of Two *Pseudonocardia* Phylotypes Associated with *Acromyrmex* Leafcutter Ants Reveals Their Biosynthetic Potential

**DOI:** 10.3389/fmicb.2016.02073

**Published:** 2016-12-26

**Authors:** Neil A. Holmes, Tabitha M. Innocent, Daniel Heine, Mahmoud Al Bassam, Sarah F. Worsley, Felix Trottmann, Elaine H. Patrick, Douglas W. Yu, J. C. Murrell, Morten Schiøtt, Barrie Wilkinson, Jacobus J. Boomsma, Matthew I. Hutchings

**Affiliations:** ^1^School of Biological Sciences, University of East Anglia (UEA)Norwich, UK; ^2^Centre for Social Evolution, University of CopenhagenCopenhagen, Denmark; ^3^Department of Molecular Microbiology, John Innes CentreNorwich, UK; ^4^State Key Laboratory of Genetic Resources and Evolution, Kunming Institute of ZoologyKunming, China; ^5^School of Environmental Sciences, University of East Anglia (UEA)Norwich, UK

**Keywords:** leafcutter ants, antibiotics, actinomycetes, *Pseudonocardia*, nystatin, polyene, genome mining, *Acromyrmex*

## Abstract

The attine ants of South and Central America are ancient farmers, having evolved a symbiosis with a fungal food crop >50 million years ago. The most evolutionarily derived attines are the *Atta* and *Acromyrmex* leafcutter ants, which harvest fresh leaves to feed their fungus. *Acromyrmex* and many other attines vertically transmit a mutualistic strain of *Pseudonocardia* and use antifungal compounds made by these bacteria to protect their fungal partner against co-evolved fungal pathogens of the genus *Escovopsis*. *Pseudonocardia* mutualists associated with the attines *Apterostigma dentigerum* and *Trachymyrmex cornetzi* make novel cyclic depsipeptide compounds called gerumycins, while a mutualist strain isolated from derived *Acromyrmex octospinosus* makes an unusual polyene antifungal called nystatin P1. The novelty of these antimicrobials suggests there is merit in exploring secondary metabolites of *Pseudonocardia* on a genome-wide scale. Here, we report a genomic analysis of the *Pseudonocardia* phylotypes Ps1 and Ps2 that are consistently associated with *Acromyrmex* ants collected in Gamboa, Panama. These were previously distinguished solely on the basis of 16S rRNA gene sequencing but genome sequencing of five Ps1 and five Ps2 strains revealed that the phylotypes are distinct species and each encodes between 11 and 15 secondary metabolite biosynthetic gene clusters (BGCs). There are signature BGCs for Ps1 and Ps2 strains and some that are conserved in both. Ps1 strains all contain BGCs encoding nystatin P1-like antifungals, while the Ps2 strains encode novel nystatin-like molecules. Strains show variations in the arrangement of these BGCs that resemble those seen in gerumycin gene clusters. Genome analyses and invasion assays support our hypothesis that vertically transmitted Ps1 and Ps2 strains have antibacterial activity that could help shape the cuticular microbiome. Thus, our work defines the *Pseudonocardia* species associated with *Acromyrmex* ants and supports the hypothesis that *Pseudonocardia* species could provide a valuable source of new antimicrobials.

## Introduction

Almost all antibiotics currently in clinical use are derived from the secondary metabolites of a group of soil bacteria called actinomycetes, but the discovery of these strains and their natural products (NPs) peaked in the 1950s. Since then, problems of rediscovery of extant strains and compounds has led to a decline in the discovery of new classes of metabolites. As a result, few new antibiotics have made it to market in the last 50 years. More recently, however, the field of NP discovery has been revitalized by large scale genome sequencing, which has revealed that actinomycetes express less than 25% of their secondary metabolite biosynthetic gene clusters (BGCs) *in vitro* (Doroghazi et al., 2014). Genome mining for novel BGCs in soil actinomycetes isolated over the last 80 years, plus new actinomycete strains isolated from under-explored environments, promises to yield 1000s of new NPs, including new anti-infective drugs (Katz and Baltz, 2016). One promising new approach is to genome mine strains that have co-evolved with their eukaryotic hosts. Such symbiotic relationships are known as protective mutualisms, because the plant or animal host houses, feeds, and sometimes vertically transmits the bacteria in exchange for antibiotics that protect them against infection (Clardy et al., 2009; Kaltenpoth, 2009; Seipke et al., 2011b). Arguably, one of the best characterized examples are the protective mutualisms between the attine ants of South and Central America and their vertically transmitted strains of *Pseudonocardia* (Cafaro et al., 2011; Caldera and Currie, 2012).

**FIGURE 1:**
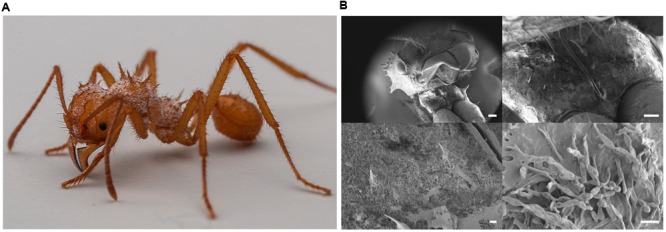
**Imaging of filamentous bacteria associated with *Acromyrmex echinatior* ants. (A)** Photographs of an *A. echinatior* callow large worker ant reveals a dusty white covering of bacteria. (Image: David Nash). **(B)** Scanning electron microscopy of an *A. echinatior* worker reveals filamentous bacteria dominate the microbiome (Images: Kim Findlay). Top left panel: wide view of a worker, scale bar equals 200 μm. Top right panel: the laterocervical plates, scale bar equals 100 μm. Bottom left panel: zoomed in view of the filamentous bacteria on the laterocervical plates, scale bar equals 10 μm. Bottom right panel: high magnification image of filamentous *Pseudonocardia* on the surface of an *A. echinatior* worker, scale bar equals 2 μm.

The common ancestor of attine ants developed fungiculture 50–60 million years ago, leading to the tribe Attini, which consists of ca. 15 genera and more than 230 species (Schultz and Brady, 2008; Nygaard et al., 2016). The most evolutionary derived attines are the genera *Acromyrmex* and *Atta* which are known as leafcutter ants because they actively cut fresh leaves and feed them to their co-evolved symbiotic fungus *Leucoagaricus gongylophorus*. In return for the maintenance and manuring services provided by the ant farmers, the fungal cultivar produces gongylidia that are rich in fats and sugars and which the ants harvest as the sole food source for their larvae and queen (Schiøtt et al., 2010; De Fine Licht et al., 2014). *Acromyrmex* ants grow *Pseudonocardia* on their cuticles (**Figure 1**), housed in specialized crypts that are connected to subcuticular glands through which the hosts likely provide nutrients (Currie et al., 2006; Andersen et al., 2013). The *Pseudonocardia* strain provides the ants with at least one antifungal compound and the ants use it to protect their fungus against disease, in particular from the specialized fungal pathogen *Escovopsis* (Currie et al., 2003; Oh et al., 2009; Barke et al., 2010; Sit et al., 2015).

*Pseudonocardia* are considered rare actinomycetes because they are hard to isolate from soil, and there are relatively few (<20) available genome sequences for this genus (Barke et al., 2010, 2011; Sit et al., 2015). Like other filamentous actinomycetes, they grow as multicellular hyphae and reproduce under nutrient stress conditions by erecting aerial hyphae that undergo cell division to form spores. The spores provide an effective dispersal mechanism for these non-motile bacteria. In the well-studied actinomycete genus *Streptomyces*, antibiotic production occurs at the onset of sporulation, but little is known about the developmental life-cycle of *Pseudonocardia* or the secondary metabolites they encode. It was recently discovered that *Pseudonocardia* mutualists of the lower attine *Apterostigma dentigerum* and the basal higher non-leafcutter attine *Trachymyrmex cornetzi* make cyclized depsipeptide antifungals called dentigerumycin and gerumycins, respectively, which are closely related in structure (Oh et al., 2009; Sit et al., 2015). In addition, we have previously reported that a *Pseudonocardia* mutualist of *Acromyrmex octospinosus* ants collected in Trinidad also makes a polyene antifungal named nystatin P1 (Barke et al., 2010). Nystatin P1 is closely related to another polyene called NPP, which was subsequently identified in a strain called *Pseudonocardia autotrophica* of unknown origin (Lee et al., 2012). These polyenes are assembled by a polyketide synthase (PKS) and are very different to gerumycins, but they are closely related to the widely used antifungal agent nystatin A1 made by *Streptomyces noursei* with nystatin P1 having an extra hexose sugar attached to its mycosamine moiety when compared to nystatin A1 (Brautaset et al., 2000; Barke et al., 2010). The BGC for nystatin P1 and NPP encodes an additional glycosyl transferase which attaches the second deoxysugar to the mycosamine already attached to the nystatin backbone (Barke et al., 2010; Kim et al., 2015). These additional tailoring enzymes have potential for bioengineering nystatin A1 producers, because the addition of an additional deoxysugar makes NPP 300 times more soluble in water (Lee et al., 2012). Polyenes bind to ergosterol and form channels in the fungal cell membrane, which ultimately kills the fungus. They have broad spectrum activity, and resistance to polyenes is rare, including for clinically important molecules like amphotericins, which likely makes them valuable defense molecules against pathogens like *Escovopsis*, which are specifically adapted to exploit attine cultivars.

In this work we used whole-genome sequencing and analysis to investigate the secondary metabolites encoded by *Pseudonocardia* mutualists of *Acromyrmex echinatior* ants. All *Acromyrmex* colonies were collected from a population in Gamboa, Panama and are associated with one of two *Pseudonocardia* phylotypes distinguished by 16S rRNA gene sequencing and named Ps1 and Ps2. All ants within a single colony share the same phylotype, making the two strains almost completely mutually exclusive at the colony level (Poulsen et al., 2005; Andersen et al., 2013). Little is known about these strains, but previous studies have found clear differences between Ps1 and Ps2 at the 16S rRNA gene level (Poulsen et al., 2005). Also, older worker ants carrying a Ps1 strain are associated with a more diverse host cuticular microbiome than those carrying Ps2 (Andersen et al., 2013), and there are some colony-level behavioral differences in the ant hosts that are related to disease control performance (Andersen et al., 2015). We reasoned this could be due to differences in the *Pseudonocardia* symbiont growth rates, the antibiotics they encode, or both, and this prompted us to investigate the differences between the genomes of the two phylotypes.

In this study we report the first detailed analysis of the Ps1 and Ps2 *Pseudonocardia* phylotypes that are associated with *Acromyrmex* leafcutter ants and show that they are sufficiently different as to represent two distinct species with distinct BGC profiles. Ps1 strains encode 14–15 BGCs, of which seven are conserved in Ps1 but not found in Ps2 strains. Ps2 strains encode 11–15 BGCs, and five of these are unique to Ps2. Six BGCs are shared between Ps1 and Ps2 strains and they all encode nystatin-like gene clusters. However, the Ps1 strains encode nystatin P1, while the Ps2 strains are predicted to make novel variants of nystatin. We also show that both phylotypes have antibacterial activity *in vitro*, which may help them to monopolize the entire cuticular microbiomes of large workers (Andersen et al., 2013) as predicted by Scheuring and Yu (2012). The antibacterial NPs cannot be predicted from the BGC sequences alone, which suggests they have the potential to yield new classes of antibiotics.

## Materials and Methods

### Ant Collection and Bacterial Isolations

This study used a total of 23 *A. echinatior* colonies collected during fieldwork in the Gamboa area of Soberania National Park, Panama, between 2001 and 2014. Colonies were subsequently maintained at the University of Copenhagen, in climate-controlled rooms (ca. 70% humidity, 25°C), with the exception of the Ae707 sub-colony, which was set up in the laboratory for ∼12 months at the University of East Anglia, and fed bramble leaves, apple, and dry rice, similar to the colonies maintained in Copenhagen. *Pseudonocardia* strains were isolated from 1–2 *A. echinatior* worker ants from each of the 23 captive colonies by direct contact of a needle with the laterocervical plates. Bacteria were cultured from 1–2 single worker ants taken from each colony (**Figure 1**). They were plated onto Lennox agar (20 g agar, 10 g Tryptone, 5 g Yeast Extract, 5 g NaCl made up to 1 L with distilled water and then autoclaved) and incubated at 30°C until small white *Pseudonocardia* colonies were visible. Isolates were re-streaked until pure and then spread for confluent lawns on MS agar (20 g agar, 20 g mannitol, 20 g soya flour made up to 1 L with tap water and autoclaved twice) to induce sporulation. Spores were collected by gentle washing in 3 mL sterile 20% (v/v) glycerol using sterile cotton buds and then transferred to 2 mL screw cap tubes and stored at -20°C. Phylotype identification of *Pseudonocardia* was performed by PCR amplification of the 16S rRNA gene using primers PRK341F (5′-CCT ACG GGR BGC ASC AG-3′) and MPRK806R (5′-GGA CTA CNN GGG TAT CTA AT-3′).

### Antibacterial Bioassays

To simulate challenges to *Pseudonocardia* dominated leafcutter ant cuticles and thereby assess invasion of *Pseudonocardia* microbiomes by other bacteria, we devised the following bioassays. *Pseudonocardia* spores were spread onto sterile cellophane disks placed on top of MS agar plates and grown at 30°C for 7 days until they covered the plates in a confluent lawn. The cellophane disks were then peeled off to remove the *Pseudonocardia* and the agar plates were replenished with nutrients by adding 200 μl Lennox broth and then air dried to get them ready for the bioassays. Unicellular bacterial test strains were grown from frozen glycerol stocks overnight in 10 mL Lennox broth at 37°C. They were then sub-cultured 1:100 in 10 mL fresh Lennox broth. Subcultures were grown for ∼4.5 h, after which an OD_600_ was measured, where OD_600_ = 1 was assumed to represent 8 × 10^9^ cells. Working stocks were then made of the test strains to 10^8^ cells. Equivalently, titred spore stocks of *Streptomyces* (Kieser et al., 2000) were diluted to a working concentration of 10^8^ spores. Subsequently, 10 μl of the working stocks of all 10 bacterial test strains (five unicellular and five *Streptomyces*) were added to the plates by pipetting and the plates were incubated for 2 days at 30°C.

### Genome Sequencing and Assembly

High quality draft genomes of strains Ae150A_Ps1, Ae168_Ps1, Ae263_Ps1, Ae356_Ps1, Ae331_Ps2, Ae406_Ps2, Ae505_Ps2, and Ae706_Ps2 were obtained using Illumina technology. The two strains Ae707_Ps1 and Ae717_Ps2 were sequenced using PacBio to obtain high quality draft genomes to use as a reference for comparison. *Pseudonocardia* spores were inoculated onto Lennox agar plates covered with sterilized cellophanes. After 2–3 weeks of incubation at 30°C the cellophanes were removed and the mycelium was scraped off into sterile tubes. The salting out method was used to prepare genomic DNA from these mycelium samples (Kieser et al., 2000). Minor modifications included the addition of achromopeptidase and DNase-free RNase (Qiagen) to the lysozyme step, as well as longer incubation steps for lysozyme and proteinase K treatment. DNA was quantified and quality checked using a Nanodrop 2000c spectrophotometer. Illumina sequencing of DNA was carried out at the DNA Sequencing Facility, Department of Biochemistry, University of Cambridge, UK, using TruSeq PCR-free and Nextera Mate Pair libraries and a MiSeq 600 sequencer. Genome assembly was performed using Roche Newbler v3.0, scaffolds were polished using PILON version 1.13, and reads were mapped using Burrows–Wheeler transformation version 0.7.12-r1039 (Walker et al., 2014). PacBio sequencing of DNA was carried out at the Earlham Institute, Norwich Research Park, Norwich, UK, using SMRT bell adaptor libraries and PacBio standard large insert conditions, with 2–3 SMRT cells per sample. PacBio data were assembled using SMRT analysis software (Pacific Biosciences of California, Inc.) incorporating HGAP3 (Chin et al., 2013). Assembled genomes were submitted to antiSMASH for BGC analysis and individual BGCs were compared across all strains using MultiGeneBlast (Weber et al., 2015). Each BGC was downloaded as a GenBank file from the antiSMASH output and amino acid sequences from those clusters were used to generate a database. The database was then searched using individual BGCs as query sequences using the MultiGeneBlast algorithm. 16S rRNA, *rpsL* and *rpoB* sequences were aligned using ClustalW 2.1 (Larkin et al., 2007). Blast analysis was carried out using ncbi-blast-2.2.31+ (Camacho et al., 2009).

## Results

### Isolation of Mutualist *Pseudonocardia* Strains

The large callow workers of *A. echinatior* are covered in filamentous *Pseudonocardia* that are visible as a whitish covering on the cuticle (**Figure 1**) and can be cultured on nutrient agar. For a few weeks after hatching the filamentous bacterial growth can also be seen all over the worker ant cuticles and the rest of the body using scanning electron microscopy (**Figure 1B**; Poulsen et al., 2003). The *A. echinatior* workers typically have a concentration of *Pseudonocardia* on their laterocervical plates, so we scraped the plates of 1–2 workers taken from 23 separate *A. echinatior* colonies to isolate their mutualist strains. We consistently isolated either a single Ps1 or Ps2 *Pseudonocardia* strain from 22 out of the 23 ant colonies but for colony Ae_707, the only colony reared in Norwich rather than Copenhagen, we isolated both Ps1 and Ps2 strains. This result matched an earlier survey (Andersen et al., 2015), which had a single double infection in a sample of 19 colonies of Panamanian *A. echinatior* and *A. octospinosus*. In total, we isolated 24 strains of *Pseudonocardia*. We then selected five Ps1 strains and five Ps2 strains for genome sequencing (**Table 1**); these included six strains originating from colonies also used in the Andersen et al. (2015) study. Under laboratory culture conditions, Ps2 strains generally grew faster and generated more biomass than Ps1 strains, but all the *Pseudonocardia* isolates grew weakly compared to *Streptomyces* species and to *Pseudonocardia* strains isolated previously from *A. octospinosus* worker ants (Barke et al., 2010). Only one of the *Pseudonocardia* strains sequenced grew in liquid culture, and we could not conjugate any vectors into the strains.

**Table 1:** Bacterial strains used in this work.

Strain	Description	GenBank accession no.	Source
Ae150A_Ps1	*Pseudonocardia* Ps1 mutualist strain	MCIJ00000000	This study
Ae168_Ps1	*Pseudonocardia* Ps1 mutualist strain	MCIK00000000	This study
Ae263_Ps1	*Pseudonocardia* Ps1 mutualist strain	MCIL00000000	This study
Ae356_Ps1	*Pseudonocardia* Ps1 mutualist strain	MCIN00000000	This study
Ae707_Ps1	*Pseudonocardia* Ps1 mutualist strain	MCIR00000000	This study
Ae331_Ps2	*Pseudonocardia* Ps2 mutualist strain	MCIM00000000	This study
Ae406_Ps2	*Pseudonocardia* Ps2 mutualist strain	MCIO00000000	This study
Ae505_Ps2	*Pseudonocardia* Ps2 mutualist strain	MCIP00000000	This study
Ae706_Ps2	*Pseudonocardia* Ps2 mutualist strain	MCIQ00000000	This study
Ae717_Ps2	*Pseudonocardia* Ps2 mutualist strain	MCIS00000000	This study
S4	*Streptomyces* S4	CADY00000000	Seipke et al., 2011a
KY5	*Streptomyces* KY5	N/S	Seipke et al., 2013
*Streptomyces lividans*	Soil-derived *Streptomyces*	N/S	John Innes Centre, Norwich, NR4 7UH, UK
*Streptomyces coelicolor* M145	Soil-derived *Streptomyces*	AL645882	John Innes Centre, Norwich, NR4 7UH, UK (Bentley et al., 2002)
*Streptomyces venezuelae* ATCC 10712	Soil-derived *Streptomyces*	NC_018750	John Innes Centre, Norwich, NR4 7UH, UK
KY8	KY8 *Kocuria* sp.	N/S	Seipke et al., 2013
KY12	KY12 *Pseudomonas*	N/S	Seipke et al., 2013
KY15	KY15 *Bacillus*	N/S	Seipke et al., 2013
KY17	KY17 *Serratia*	N/S	Seipke et al., 2013
KY20	KY20 *Staphylococcus*	N/S	Seipke et al., 2013

*The names of the 10 Panamanian strains are combinations of ant colony identifiers and *Pseudonocardia* phylotypes.*

### Ae707_Ps1 and Ae706_Ps2 Strains Have Antibacterial Activity

Culture-dependent and independent studies have shown that *Pseudonocardia* dominate the cuticle of *A. echinatior* ants, but closely related *Streptomyces albus* strains have also been isolated from *Acromyrmex* ants collected from different locations in South America (Haeder et al., 2009; Sen et al., 2009; Barke et al., 2010). It is still not clear if these are mutualist strains, but they have been shown to produce antibiotics on the surface of worker ants using MALDI-TOF, which means they are growing and metabolically active on the ant cuticle (Schoenian et al., 2011). Domination of the ant cuticle by *Pseudonocardia* is facilitated by vertical transmission and inoculation of worker ants within 24 h of eclosing (Marsh et al., 2014). Subsequently, the *Pseudonocardia* blooms over the entire surface of the ant before eventually shrinking back to the laterocervical plate when large workers are ca. 6 weeks old and start leaving the nest to become foragers (Poulsen et al., 2003). One way in which the *Pseudonocardia* mutualist might inhibit other bacteria from gaining a foothold on the cuticle is by making antibacterial compounds in addition to the antifungals used to suppress *Escovopsis* (Barke et al., 2011; Scheuring and Yu, 2012). To test this hypothesis, we devised resistance bioassays in which *Pseudonocardia* strains were grown on sterile, porous cellophane disks placed on top of agar plates, allowing secondary metabolites produced by *Pseudonocardia* to permeate the agar.

**FIGURE 2:**
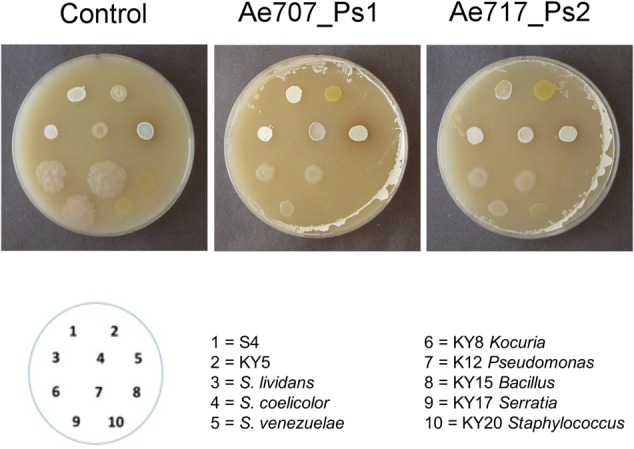
**Resistance assays using *Pseudonocardia* strains show that both phylotypes have antibacterial activity.** 1–5 are *Streptomyces* strains isolated originally from either soil or ant associated sources. 6–10 are unicellular bacteria isolated from ant associated sources. *Streptomyces* are undisturbed in growth on lawns of *Pseudonocardia* Ae707_Ps1 and Ae717_Ps2 relative to the blank control, but unicellular bacteria show suppression.

After 7 days, the cellophane and *Pseudonocardia* lawns were removed, the agar was replenished with nutrients by applying Lennox broth, and the surfaces of the agar plates were dried before inoculation with test strains of unicellular bacteria or filamentous *Streptomyces* species. Some of these were isolated previously from fungus-growing ant nests whereas some were soil-isolated *Streptomyces* strains (**Table 1**). The cellophane disks allowed exchange of nutrients and secondary metabolites, including antibiotics that might inhibit the growth of other bacteria. Results showed that both Ps1 and Ps2 strains could partially inhibit the growth of Gram-negative and Gram-positive bacteria, but the Ps1 strains appeared to be more potent, at least under the conditions used here. This suggests that both strains are producing antibacterial compounds, and since we know that typically ≤25% of BGCs are expressed *in vitro* it seemed likely that in nature they would exhibit more potent activity against other strains, particularly since the genomes of all 10 sequenced *Pseudonocardia* strains encode multiple potential bacteriocins. Consistent with our predictions (Barke et al., 2011), only the unicellular bacterial strains were inhibited, whereas growth of the *Streptomyces* strains was unaffected or even enhanced compared with the control plate (**Figure 2**). This is consistent with the fact that *Streptomyces* strains carry multiple antibiotic resistance genes and can invade the ant cuticular microbiome (Wright, 2007; Barke et al., 2011).

### Ps1 and Ps2 Strains are Phylogenetically Distinct

For all Ps1 and Ps2 strains, their draft genomes consisted of one large contig representing the majority of the chromosome (Supplementary Table S1). The additional, smaller contigs per strain could represent either unassembled parts of the chromosome or extrachromosomal plasmids. Alignment of full length 16S rRNA and *rpoB* and *rpsL* genes (Supplementary Material) showed that the Ps1 and Ps2 strains are more similar within (99.93–100%) than between strains (97.61–97.74%) for 16S rRNA (Supplementary Table S2). For *rpoB* (Supplementary Table S4), Ps1 conservation was 99.74–100.00%, Ps2 conservation was 99.91–100.00%, and between Ps1 and Ps2 there was 94.96–95.10% identity. For the relatively short *rpsL* gene (Supplementary Table S3), both Ps1 and Ps2 conservation were 100% similar within each lineage, whereas between Ps1 and Ps2, mean similarity was 92.8%. We therefore conclude that the Ps1 and Ps2 phylotypes represent two different species of *Pseudonocardia.* Since the nystatin P1 producer (Ps1 phylotype) was first characterized in detail from the *A. octospinosus* system, we suggest the name *Pseudonocardia octospinosus* for Ps1 and *Pseudonocardia echinatior* for Ps2.

### Ps1 and Ps2 Mutualists Can Be Grouped According to their Secondary Metabolite BGCs

**FIGURE 3:**
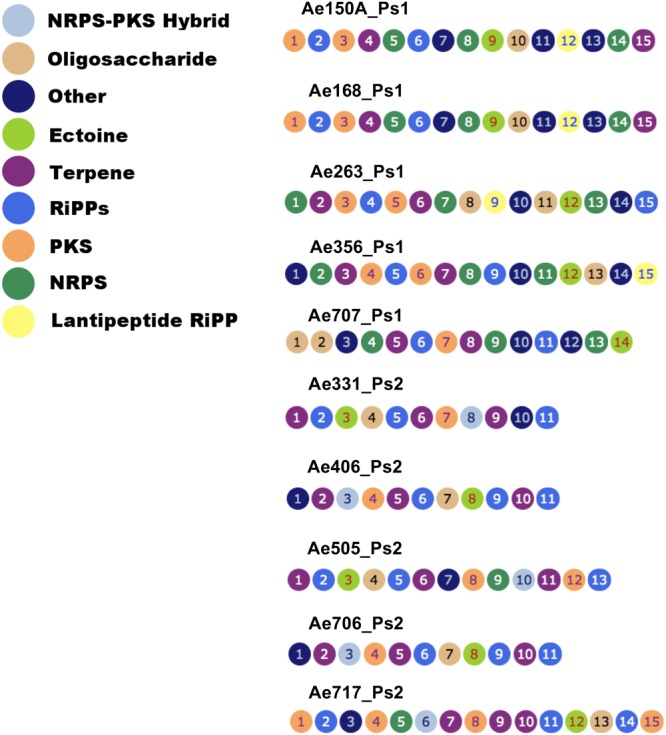
**Secondary metabolite biosynthetic gene clusters in the genomes of *Pseudonocardia* strains from the cuticle of *Acromyrmex echinatior* ants, as predicted by antismash.** Clusters appear as ordered by antismash of the DNA assemblies of *Pseudonocardia* genomes. NRPS, non-ribosomal peptide synthetase; PKS, polyketide synthases; RiPPs, ribosomally synthesized and post-translationally modified peptides.

**FIGURE 4:**
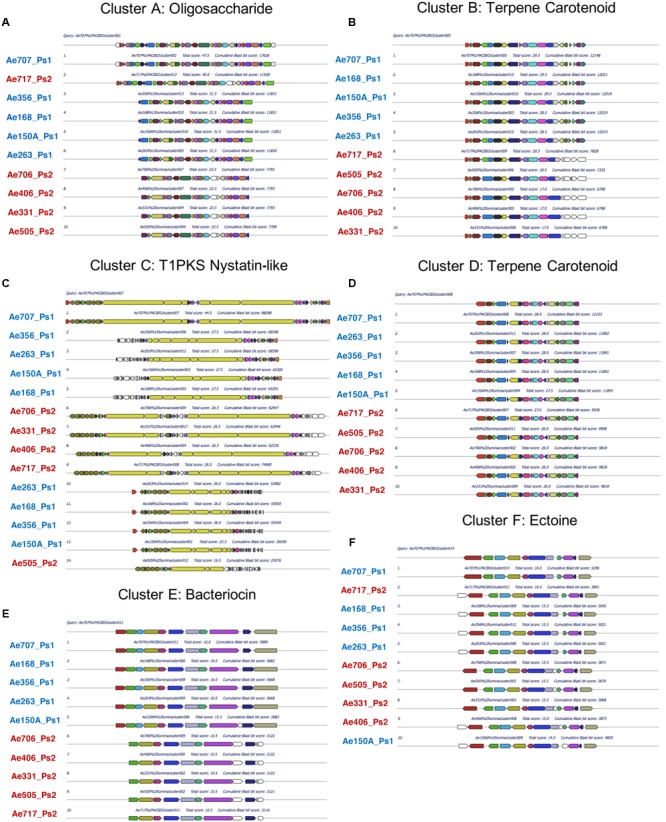
**Multigene blast outputs showing conservation of biosynthetic gene clusters shared between Ps1 and Ps2 phylotypes.** Shared clusters include; cluster A: oligosaccharide cluster **(A)**, cluster B: a terpene cluster with similarity to carotenoid BGCs **(B)**, cluster C: a nystatin-like T1PKS cluster **(C)**, cluster D: a second terpene cluster with similarity to carotenoid BGCs **(D)**, cluster E: a bacteriocin cluster **(E)**, and cluster F: a cluster predicted to make the osmoprotectant ectoine **(F)**.

**FIGURE 5:**
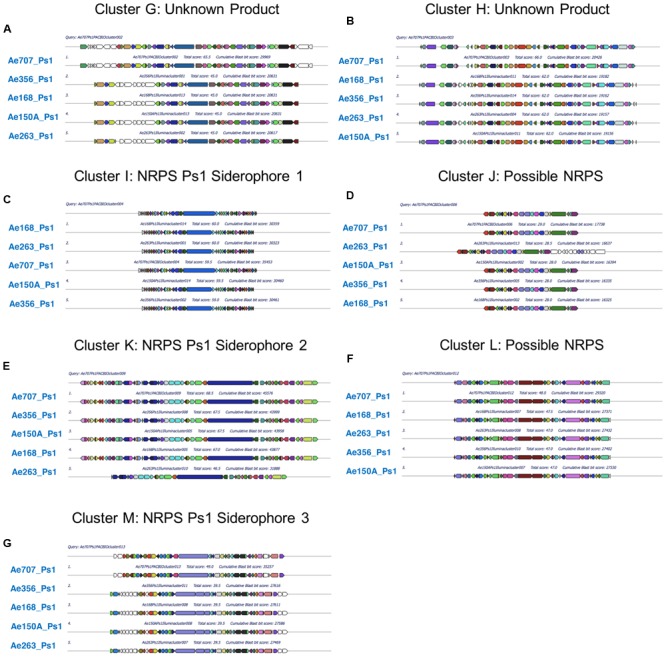
**Multigene blast outputs showing conservation of biosynthetic gene clusters shared between all Ps1 phylotypes.** Ps1 clusters include; clusters G and H: two clusters encoding unknown products **(A,B)**, cluster I: an NRPS cluster for Ps1 siderophore 1 **(C)**, cluster J: a bacteriocin cluster encoding a possible NRPS **(D)**, cluster K: an NRPS cluster for Ps1 siderophore 2 **(E)**, cluster L: a possible NRPS cluster encoding an unknown product **(F)** and cluster M: an NRPS cluster for Ps1 siderophore 3 **(G)**.

**FIGURE 6:**
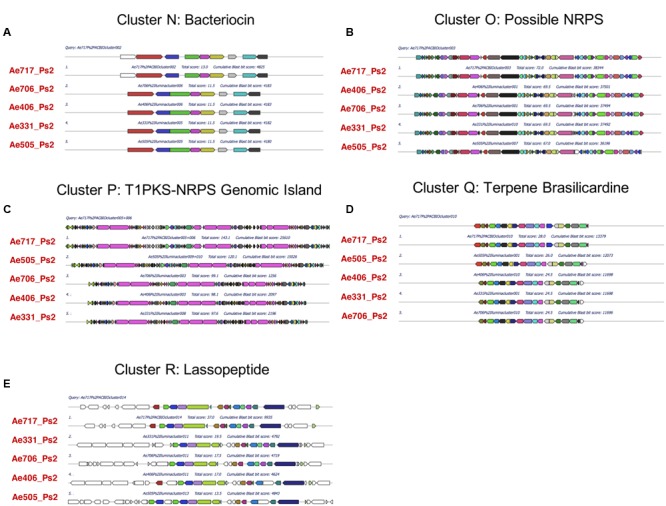
**Multigene blast outputs showing conservation of biosynthetic gene clusters shared between all Ps2 phylotypes.** Ps2 clusters include; cluster N: a bacteriocin cluster **(A)**, cluster O: a possible NRPS cluster encoding an unknown product **(B)**, cluster P: a genomic island with T1PKS and NRPS genes **(C)**, cluster Q: a terpene cluster with similarity to the brasilicardin cluster **(D)** and cluster R: a cluster for production of a possible lassopeptide **(E)**.

To determine which secondary metabolites are encoded by the *Pseudonocardia* mutualists of *A. echinatior*, we used antiSMASH 3.0 to identify the BGCs in all 10 sequenced genomes (**Figure 3**; Supplementary Figures S1–S10; Weber et al., 2015). Although, in some cases we know that antiSMASH has called the BGCs bigger than they really are, we have adopted a consistent approach of presenting all the data from the antiSMASH analysis because for most BGCs we have no idea where they start and end. AntiSMASH outputs indicated that the Ps1 strains encoded for the production of 14–15 secondary metabolites, while the Ps2 strains encoded 11–15 (**Figure 3**). In order to verify the similarity of the clusters between strains, we used MultiGeneBlast (**Figures 4–6**; Weber et al., 2015). Through shared homology and gene architecture, we deduced that six of the BGCs were shared between all 10 strains, seven are unique to Ps1, and five are unique to Ps2 (**Table 2**). The BGCs shared by all 10 *Pseudonocardia* strains (clusters A–F) include the *ectABCD* operon (**Figure 4F**, cluster F) which encodes biosynthesis of the osmoprotectants ectoine and 5′-hydroxyectoine which are common in both Gram-positive and Gram-negative bacteria and often found in actinomycetes (Galinski et al., 1985). There was a distinct bacteriocin BGC present in all Ps1 and Ps2 strains (**Figure 4E**, cluster E), and these proteinaceous molecules usually have antimicrobial activity, which can range from narrow to broad spectrum (Gross and Morell, 1971; Cotter et al., 2013). Encoded within the cluster is a peptide with a TIGR04222 domain predicted to be processed as a ribosomally encoded peptide, several nucleases and a sulfur transferase plus accessory protein that could modify the peptide backbone. Ps1 and Ps2 strains share two terpene encoding BGCs (**Figures 4B,D**, clusters B and D), hydrocarbons comprised of isoprene-derived units that have diverse bioactivities including antimicrobial activity (Gershenzon and Dudareva, 2007; Gallucci et al., 2009). One of these terpene BGCs (cluster B) has similarity to carotenoid BGCs, and encodes a polyprenyl synthetase and a phytoene synthase (Richter et al., 2015), while the other shared terpene BGC (cluster D) contains a terpene cyclase closely related to a lycopene cyclase found in carotenoid biosynthesis. This cluster also has similarity to a conserved cluster in *Rhodococcus* bacteria. The Ps1 and Ps2 strains also share an oligosaccharide cluster which encodes for enzymes associated with the biosynthesis of deoxysugars and glycosyltransferases, but for which we were unable to predict a product (**Figure 4A**, cluster A).

**Table 2:** Biosynthetic gene clusters (BGCs) encoded by the five Ps1 and five Ps2 strains sequenced in this study.

Cluster ID	Ae707Ps1 BGC no.	Ae717Ps2 BGC no.	Type	Predicted product and function
**BGCs shared by Ps1 and Ps2 strains**				
A	1	13	Oligosaccharide	Unknown
B	5	9	Terpene	Carotenoid
C	7	8	T1PKS	Nystatin-like polyene
D	8	7	Terpene	Carotenoid
E	11	11	Bacteriocin	Probably antibacterial
F	14	12	Ectoine	Osmoprotectant
**BGCs shared by Ps1 strains**				
G	2	-	Oligosaccharide	Unknown
H	3	-	Other	Unknown
I	4	-	NRPS	Siderophore
J	6		Bacteriocin	Possible NRPS
K	9	-	NRPS	Siderophore
L	12	-	Other	Possible NRPS
M	13	-	NRPS	Siderophore
**BGCs shared by Ps2 strains**				
N	-	2	Bacteriocin	Probably antibacterial
O	-	3	Other	Possible NRPS
P	-	5+6	T1PKS-NRPS	Genomic Island possibly encoding siderophores
Q	-	10	Terpene	Weak similarity to the terpene brasilicardin A
R	-	14	Lassopeptide	Unknown

**Cluster ID**	**Strain**	**Cluster no.**	**Type**	**Predicted product and function**

**Other BGCs**				
S	Ae707_Ps1	10	Other	Unknown
T	Ae717_Ps2	1	Iterative T1PKS	Similarity to galbonolides; antifungal macrolactones
U	Ae717_Ps2	4	T1PKS	Unknown
V	Ae717_Ps2	15	T2PKS	Unknown
W	Ae150A_Ps1	12	Lantipeptide	Unknown

All five Ps1 strains share three non-ribosomal peptide synthetase (NRPS) BGCs that are not present in the Ps2 strains (clusters I, K, and M). We propose that these BGCs synthesize metal binding siderophore molecules, likely to sequester ferrous iron or other metal ions, particularly since these BGCs include transporters and other siderophore associated genes. The cluster for Ps1 siderophore 1 (**Figure 5C**, cluster I) is predicted to have the initial peptide arrangement of 2,3-DHB-ser-ala-(ala)-orn (second ala missing from Ae150A, Ae168, Ae263, and Ae356). Due to the predicted incorporation of a catechol unit (2,3-dihydroxybenzoic acid; 2,3-DHBA), a feature commonly found in siderophores and amino acids likely to interact with metal ions such as serine and ornithine, we propose a role in iron-acquisition for the secondary metabolite related to this cluster. The cluster for Ps1 siderophore 2 (**Figure 5E**, cluster K) has similarity with the erythrochelin BGC which was first identified in *Saccharopolyspora erythraea* (Lazos et al., 2010; Robbel et al., 2010). The cluster for Ps1 siderophore 2 has an NRPS that we predict generates a peptide arrangement of orn-ser-orn-orn, similar to the sequence for erythrochelin. However, there are a further 2–3 additional adenylation domains encoded by additional genes. Finally, the cluster for Ps1 siderophore 3 (**Figure 5G**, cluster M) we predict makes a tripeptide, with arrangement 2,3-DHBA then a polar residue either ser or thr, and lastly orn or arg. The last two amino acids we predict are epimerized to the D-configuration. We presume that iron is limited on ant cuticles, so siderophores would potentially play an important role for resource acquisition in these mutualist strains; efficient scavenging of iron and other metals by these species may also function to inhibit the growth of other microbes and therefore contribute to an anti-microbial phenotype. All Ps1 strains shared a novel cluster that was not present in any of the sequenced Ps2 strains (**Figure 5A**, cluster G), which contains several glycosyl transferases and enzymes capable of activating carboxylic acids (adenylation-like domains), and makes an unknown product. Ps1 strains also had an additional BGC (**Figure 5B**, cluster H) that we are uncertain makes a secondary metabolite and may be an artifact of the antiSMASH search algorithm. All the Ps1 strains encoded a further two BGC clusters that encode possible NRPSs. One cluster (**Figure 5D**, cluster J) encodes two adenylating enzymes, one of these shows similarity to an NRPS. This cluster is classified by antiSMASH as a bacteriocin cluster on account of containing a peptide that has similarity to the bacteriocin lincocin M18 produced by *Brevibacterium linens* (Valdés-Stauber and Scherer, 1994). The other BGC (**Figure 5F**, cluster L) that was maintained across all Ps1 strains has NRPS-like genes and possibly encodes an adenylation domain, a carrier protein and a cyclase. We predict it catalyzes synthesis of a dipeptide.

All five Ps2 strains share five BGCs that are not present in Ps1 strains (**Table 2**; **Figure 6**), including one bacteriocin BGC (**Figure 6A**, cluster N) which also has similarity to the bacteriocin lincocin M18 but with only 74–78% similarity to the gene in the Ps1 strains (cluster L). This cluster does not have the same surrounding genes, including the adenylating enzymes. All Ps2 strains have a BGC (**Figure 6B**, cluster O) that has two adenylating proteins that could act as an NRPS as well as several oxidative proteins. All Ps2 strains also contained a genomic island containing BGCs with Type 1 PKS (T1PKS) and NRPS genes (**Figure 6C**, cluster P). In Ae717_Ps2 and Ae505_Ps2, this genomic island was called as two separate and adjacent BGCs by antiSMASH, but they were called as one TIPKS-NRPS hybrid cluster in Ae331_Ps2, Ae406_Ps2, and Ae706_Ps2. It is likely that these are in fact three separate BGCs. In Ae717_Ps2 cluster 5 (cluster P, part 1) makes a pentapeptide (predicted sequence; ala-thr-orn-ser/thr-orn). Being rich in threonine and ornithine residues suggests the resulting peptide is likely to bind metal. Consistent with this, proteins immediately adjacent include a siderophore interacting protein member involved in metal acquisition/utilization. Ae717_Ps2 cluster 5 (cluster P, left) is separated from cluster 6 (cluster P, center/right) by apparent primary metabolism genes. Ae717_Ps2 cluster 6 can be split into two subclusters (cluster P, center/right); subcluster 6A (cluster P, center) is an NRPS-PKS hybrid consisting of modules for assembly of a glycine starter unit followed by five PKS extension steps. The PKS appears to have an unprecedented hybrid in *cis* and in *trans* acyltransferase architecture. Ae717_Ps2 subcluster 6A also has a type 1 glycosyl transferase and other deoxysugar genes associated with it. Subcluster 6B (cluster P, right) encodes three co-linear NRPS genes that likely make a hepta/hexa-peptide including a 2,3-DHBA unit followed by cys-ala-ser/thr-orn-(D-orn) residues (the second orn is absent from Ae331_Ps2, A406_Ps2, and Ae706_Ps2). This arrangement strongly suggests a metal binding peptide likely to act as a siderophore. Ps2 strains also had a unique terpene BGC (**Figure 6D**, cluster Q) with some similarity to the brasilicardin BGC. Brasilicardin is a diterpenoid molecule possessing immunosuppressive activity produced by *Nocardia brasiliensis* (Shigemori et al., 1998; Hayashi et al., 2008). The Ps2 cluster could make a molecule with a cyclic core and additional elaboration. The last Ps2-specific BGC is predicted to encode biosynthesis of a lassopeptide (**Figure 6E**, cluster R), a ribosomally encoded peptide that is post-translationally modified. Lassopeptides form a distinct topology where the *N*-terminus is bound covalently to an aspartate or glutamate side chain further back in the peptide. The *C*-terminal end of the molecule is then threaded through the ring forming the “lasso” structure. The Ps2 BGC encodes an asparagine synthase that could perform the peptide cyclization, and there is a GCN5 *N*-acetyltransferase that could perform the proteolytic action of peptide processing. We also found a small open reading frame upstream of the asparagine synthase that could provide the starting peptide molecule. Lassopeptides belong to a class of natural product known as ribosomally-synthesized and post-translationally-modified peptides (RiPPs) and have been recognized as an important class of molecules because their intrinsic protease resistance gives them great potential as scaffolds for drug design (Hegemann et al., 2015; Piscotta et al., 2015). This BGC is encoded on a fragment of the genome sequence that we predict is a plasmid; this lassopeptide BGC could therefore be passed around a population of *Pseudonocardia* strains in a functionally dependent manner in response to environmental circumstances.

In addition to the BGCs that were found in either Ps1, Ps2 or both there were several BGCs specific to individual strains or subsets of strains (**Table 2**; Supplementary Figure S11). BGC 10 in strain Ae707_Ps1 is unique (Supplementary Figure S11A, cluster S), and antiSMASH did not find similarity with any known BGCs. Inspection of this cluster finds an AMP-dependent synthetase/ligase that may function as an NRPS. Strain Ae717_Ps2 encoded a unique BGC (Supplementary Figure S11B, cluster T) with similarity to the BGC cluster for production of the galbonolides from *Streptomyces galbus* (Karki et al., 2010). Galbonolides are anti-fungal macrolactones made by an iterative T1PKS. The Ae717_Ps2 BGC encodes an appropriate iterative T1PKS and other associated genes to make a related anti-fungal macrolactone. Strain Ae717_Ps2 also contained an additional BGC encoding a T1PKS (Supplementary Figure S11C, cluster U), and only Ps2 strain Ae505_Ps2 shared this cluster. We predict the product of this BGC will be a highly unsaturated pentaketide derived from a 3-amino-5-hydroxybenzoic acid (AHBA) starter unit and four extension sites using malonyl-CoA as the substrate. This is highly likely to be modified by additional post-PKS genes encoded in the cluster. Ae717_Ps2 contained a unique T2PKS BGC that is highly likely to encode for a glycosylated T2PKS (Supplementary Figure S11D, cluster V). The DNA fragment encoding this T2PKS contains similarity to plasmid sequences so could be a recent addition to the genome of this strain. A BGC predicted to make a lantipeptide (Supplementary Figure S11E, cluster W) was found in a subset of the Ps1 strains (Ae150A_Ps1, Ae168_Ps1, Ae263_Ps1, and Ae356_Ps1). Lantipeptides are RiPPs with post-translational modifications in which amino acid residues are cross-linked via thioether bridges. The Ps1 lantibiotic clusters contain lantibiotic biosynthesis enzymes including the dehydratase and cyclase activities that are required to form lanthionine bridges (Karakas Sen et al., 1999; Li et al., 2006).

### Ps1 and Ps2 Phylotypes Encode Different Polyene Antifungals

Perhaps the most intriguing feature of all 10 sequenced *Pseudonocardia* strains is the presence of nystatin-like biosynthesis gene clusters in their genome (**Figure 4C**). All Ps1 strains likely encode molecules similar to Nystatin P1 and NPP, previously reported as secondary metabolites from an *A. octospinosus* mutualist strain (Barke et al., 2010) and from *P. autotrophica* (Kim et al., 2009), respectively. As we obtained high quality PacBio sequenced genomes of Ae707_Ps1 and Ae717_Ps2, we used the nystatin-like clusters from each as templates for comparison in order to compare these BGCs across all 10 strains. The nystatin cluster from Ae707_Ps1, proves to be mostly identical to that encoded by the *A. octospinosus* mutualist strain we identified previously (Barke et al., 2010) (data not shown). However, for comparison we performed bioinformatics analysis (Supplementary Table S5) against the NPP cluster from *P. autotrophica* (Kim et al., 2009) and demonstrate a high level of similarity between the amino acid sequences. In contrast, the nystatin-like cluster from Ae717_Ps2 showed less overall conservation with the *P. autotropica* NPP cluster (Supplementary Table S5). This prompted us to perform a detailed analysis and comparison of the polyene BGC for both phylotypes.

**FIGURE 7:**
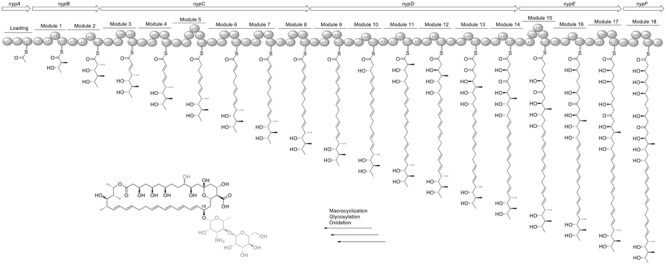
**Organization of the nystatin P1 biosynthesis gene cluster in Ae707_Ps1 and model for its biosynthesis deduced from the PKS assembly line.** ACP, acyl carrier protein; AT, acyl transferase; DH, dehydratase; ER, enoylreductase; KR, ketoreductase, KS, ketosynthase; KSS, *nys*-like loading KS; TE, thioesterase. ^∗^Domains labeled with an asterisk indicate inactive domains. Parts of the proposed structures which are pictured in gray are due to post-PKS tailoring steps.

We have analyzed the PKS architecture and predicted the assembled polyketide product of the nystatin cluster from Ae707_Ps1 (**Figure 7**). The polyene cluster has a total of 19 PKS modules and contains one ketoreductase domain (module 13) and two dehydratases (modules 17 and 18) which are predicted to be inactive due to a lack of the catalytic tyrosine (ketoreductase) and histidine (dehydrogenase) motifs, respectively. This is in conjunction with the biosynthetic cluster for NPP found in *P. autotrophica* (Kim et al., 2009). Analysis of the KR sequence motifs (Keatinge-Clay, 2007) revealed all stereocentres being consistent with the configuration in nystatin (Brautaset et al., 2000). The KR responsible for reduction leading to the hydroxyl group at position 19 shows an LDD motif (compared to the catalytically inactive LDA motif at this position in the original nystatin producer *Streptomyces noursei*). Due to its location outside of the LDD loop we consider it non-functional and therefore propose an *R*-configuration at this position in accordance with the reported configuration of nystatin. We previously documented the presence of a second glycosyl transferase (NypY) for addition of a second hexose unit to nystatin P1 (Barke et al., 2010). The NPP cluster encodes an additional glycosyl transferase (NppY) predicted to add *N*-acetyl-glucosamine (Kim et al., 2015). Accordingly, Ae707_Ps1 and all the Ps1 strains encode an additional glycosyl transferase. Heterologous expression of *nypY* in the amphotericin producer *Streptomyces nodosus* led to a new amphotericin derivative with D-mannose attached to the D-mycosamine via a β-1,4-linkage (Walmsley et al., 2016). We therefore predict the same modification leading to the product shown in **Figure 7**, and that this is identical to nystatin P1.

**FIGURE 8:**
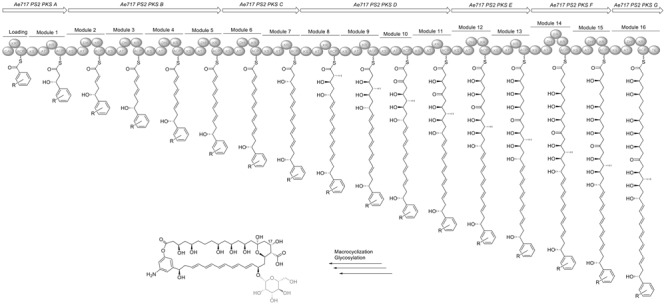
**Organization of the nystatin-like biosynthesis gene cluster in Ae717_Ps12 and model for its biosynthesis deduced from the PKS assembly line.** CAL, CoA ligase; ACP, acyl carrier protein; AT, acyl transferase; DH, dehydratase; ER, enoylreductase; KR, ketoreductase; KS, ketosynthase; TE, thioesterase. ^∗^Domains labeled with an asterisk indicate inactive domains. Parts of the proposed structures which are pictured in gray are due to post-PKS tailoring steps.

In contrast to Ps1, Ps2 strains encode a nystatin-like biosynthetic pathway that differs considerably from nystatin A1, NPP and nystatin P1 (**Figure 8**). First, the cluster seems to lack several modules compared to the BGC in Ae707_Ps1, including the loading module, the first two elongation modules, and a module responsible for the incorporation of a fully reduced acetate unit. This presumably results in a considerable structural re-arrangement of the polyketide scaffold and in a conjugated tetraene moiety in the southern part of the molecule. Instead of the loading KS^S^, there is a CoA ligase (CAL) domain encoded in the cluster, potentially responsible for providing the starter unit. Involvement of CoA ligases in PKS assembly lines is rare and usually associated with the recruitment of aromatic starter units. The CAL domain is predicted to accept AHBA as its substrate, and consistent with this a gene belonging to the 3-amino-5-hydroxybenzoic acid synthase (AHBAS) family was found downstream of the PKS coding sequence. However, we cannot completely rule out the incorporation of a distinct but structurally related aromatic moiety into the polyene. Likewise, there appears to be a considerable re-arrangement in the later module architecture with the incorporation of an additional, fully reduced acetate unit (module 14) and the substitution of a fully reduced acetate unit with an ethylene moiety (module 12). These changes presumably lead to an altered cyclization pattern for the final product. Moreover, careful analysis of all KR sequence motifs suggests a significantly altered configuration at most of the predicted stereocenters compared to nystatin. Even though the KR in module 9 contains a VDD motif instead of LDD we suggest that catalytic activity remains after the replacement of leucine by valine, and propose the assembly of an *S*-configured hydroxyl group at position 17. We suggest a potential macrolactonization with the phenol group of AHBA for cleaving off the final polyketide chain but cannot rule out the formation of the alternative amide bond and macrolactamization instead. Another intriguing feature is that all polyene clusters found in the PS2 strains contain an additional methyltransferase and an *O*-methyltransferase, a feature rather uncommon for polyene antifungals. In contrast to the cluster in all Ps1 strains, the Ps2 polyene clusters do not encode a second glycosyltransferase and therefore potentially produce a mono-glycosylated product. Based on this analysis it is highly likely that Ps2 encodes a structurally distinct polyene macrolide with altered biological properties. Unfortunately, we have not been able to induce the expression of this silent BGC in any of the Ps2 strains so far.

To compare BGCs of the *Pseudonocardia* strains sequenced by Illumina we mapped the PKS amino acid sequences of either Ae707_Ps1 or Ae717_Ps2 to genome sequences of the other Ps strains using blastx (Supplementary Table S6) and closely inspected antismash outputs and multigene blast (**Figure 4C**). Despite all the *Pseudonocardia* strains containing genes for the biosynthesis of nystatin-like molecules, there is a disparity between the genomic arrangements of the nystatin-like clusters in the *A. echinatior* associated strains that we analyzed. An analagous evolutionary example has been observed with the variation in genomic arrangement of gene fragments of the hybrid NRPS/PKS BGCs for the gerumycins. Gerumycins are produced by *Pseudonocardia* mutualists of *Apterostigma* and *Trachymyrmex* ants, where the clusters are spatially separated, in some cases on plasmids (Sit et al., 2015). We suspect this represents that *Pseudonocardia* secondary metabolite gene clusters are under constant selective pressure to evolve new compounds. Ae707_Ps1 is the only Ps1 strain to contain all the PKS genes in a single BGC. The other Ps1 strains have clusters split into two different locations on the chromosome and are also missing two PKS modules (Supplementary Table S6) suggesting they make molecules with a smaller macrolide ring size. In contrast the Ps2 phylotype strains Ae331_Ps2, Ae406_Ps2, Ae706_Ps2 and Ae717_Ps2 all contain full intact clusters at a single locus on the chromosome. However, the Ae505_Ps2 BGC contains only half of the genes expected for the nystatin-like cluster and may encode a shorter polyketide molecule or may be inactive. The polyene PKS architectures were essentially identical except for the aforementioned Ae505_Ps2 example.

## Discussion

The Ps1/Ps2 symbiosis with Panamanian *Acromyrmex* species is one of the best-studied mutualisms involving actinomycete bacteria. The two *Pseudonocardia* phylotypes were first recognized as distinct more than 10 years ago and were subsequently found to be shared by sympatric populations of *A. echinatior* and *A. octospinosus* in Panama (Poulsen et al., 2005; Andersen et al., 2015) and *A. volcanus* (Sapountzis et al., 2015). Not only are the two phylotypes shared between congener ant species, they are also consistently found in ca. 50/50 frequencies across colonies (Andersen et al., 2015), and this is reflected in our genome sequencing of strains for the present study. This is a remarkable natural distribution in light of the findings we present here on how different the two strains are at the genome level. There is some quantitative evidence (Currie et al., 2003) that growth patterns on the cuticles of large workers respond to *Escovopsis* infections, but whether there are differences between the Ps1 and Ps2 strain in the efficiency of their defenses has not been studied, although recent cross fostering experiments suggests such differences might exist (Andersen et al., 2015).

Here, we have defined the Ps1 and Ps2 phylotypes by genome sequencing five representatives from each and demonstrated that these strains are sufficiently distantly related to be classified as separate species (see Supporting Information). We have thus named them *P. octospinosus* (Ps1) and *P. echinatior* (Ps2), and shown that they are remarkably different in the type of secondary metabolites that they can produce. Seven BGCs are found only in *P. octospinosus* strains whereas five BGCs are only found in *P. echinatior* strains suggesting that ∼50% of the secondary metabolites are unique to each species. Only six BGCs are shared between the two *Pseudonocardia* species and some of these, such as ectoine, are likely part of the core genome. Our results suggest that there are two major ways in which to maintain a defensive cuticular microbiome and that both – as the field data also suggests – appear to create colonies of comparable health and reproductive fitness that coexist in the same populations. It is interesting that Ps1 and Ps2 make different siderophore/iron-binding molecules rather than conserving the same set. As cross-inoculation is feasible and produces only subtle changes in ant behavior (Andersen et al., 2015), it might be that both strains sequester iron with about equal efficiency and that the glands whose ducts end in the cuticular crypts where *Pseudonocardia* grows, secrete different mineral proportions including iron to maintain the different types of *Pseudonocardia* biofilms on large workers of *Acromyrmex* leafcutter ants. Clarifying these enigmatic issues remains a challenge to be resolved in future research.

In terms of antifungal metabolites, we obtained a detailed assessment of all the BGCs encoded by *P. octospinosus* and *P. echinatior*. Strains of *P. octospinosus* all encode forms of nystatin P1 that we first identified in a *Pseudonocardia* strain associated with *A. octospinosus* workers collected in Trinidad (Barke et al., 2010). We also compared our 10 genome sequences with those for *Pseudonocardia* strains isolated from *Apterostigma* and *Trachymyrmex* ants, which make cyclic depsipeptide antifungals called gerumycins (Oh et al., 2009; Sit et al., 2015). We were unable to identify the hybrid NRPS/PKS BGC responsible for the biosynthesis of gerumycin compounds in either *P. echinatior* or *P. octospinosus* strains. Symbionts of *Apterostigma* ants have recently been shown to encode nystatin-like antifungals called selvamicins (Van Arnam et al., 2016) and strain AL041005-10 isolated from *Trachymyrmex cornetzi* also encodes a BGC resembling the Nystatin P1 BGC. *Apterostigma* are lower attines and operate a less evolutionarily advanced form of fungiculture than *Trachymyrmex* and *Acromyrmex*, both of which farm truly domesticated crop fungi so it is intriguing that they all use polyenes. Resistance to polyene antifungals is rare and this might make them useful molecules in the fight against co-evolving *Escovopsis* parasites.

Evolution of secondary metabolite production in the fungus farming ants appears to be an interesting area, with *Pseudonocardia* genome analysis providing abundant insights. As we have found here structural arrangements of BGCs may be under constant selective pressure, and rearrangements of nystatin polyene BGCs either to spatially separated positions or by adaption of module arrangement and makeup is of significant note. We also identified several BGCs appearing on DNA with similarity to plasmids, suggesting that *Pseudonocardia* associated with fungus farming ants may be inclined to pass around BGCs via horizontal gene transfer. Genome sequencing recently revealed that *Escovopsis weberi* has a reduced genome, most likely due to its role as a parasite on the fungal garden of attine ants (de Man et al., 2016). Despite loss of many genes, however, *E. weberi* has maintained a number of genes involved in secondary metabolite biosynthesis, suggesting that colony-life in fungus-growing ants are characterized by ongoing evolutionary arms races between microbial symbionts. It cannot be ruled out that compounds produced directly by attine ants or the fungal garden strain *Leucoagaricus gongylophorus* are included in this chemical warfare as the fungal symbiont and the cuticular *Pseudonoardia* are vertically co-transmitted by default. It will be interesting in the future to examine the compounds produced by *E. weberi* and *Pseudonocardia* mutualists grown in competition on agar plates and on fungus gardens or ant cuticles using advanced imaging mass spectrometry techniques.

## Author Contributions

Experiments planned by NH, TI, MAB, DY, JM, MS, BW, JB, and MH. Experimental work carried out by NH, TI, MAB, SW, EP, and MS. Data analysis by NH, DH, FT, BW, and MH. Manuscript written by NH, TI, DH, DY, JM, MS, BW, JB, and MH.

## Conflict of Interest Statement

The authors declare that the research was conducted in the absence of any commercial or financial relationships that could be construed as a potential conflict of interest.
