# Supplementary material for: Genome Analysis of Two Pseudonocardia Phylotypes Associated with Acromyrmex Leafcutter Ants Reveals Their Biosynthetic Potential
Source: Front Microbiol. 2016 Dec 26;7:2073. doi: 10.3389/fmicb.2016.02073 (PMC5183585; doi:10.3389/fmicb.2016.02073)
Supplement: Supplementary file 1 [file Data_Sheet_1.pdf]

Genome analysis of two *Pseudonocardia* phylotypes associated with *Acromyrmex* leafcutter ants reveals their biosynthetic potential

Neil A. Holmes[1], Tabitha M. Innocent[2], Daniel Heine[3], Mahmoud Al Bassam[1], Sarah F. Worsley[1], Felix Trottmann[3], Elaine H. Patrick[1], Douglas W. Yu[1,4], J. Colin Murrell[5], Morten Schiøtt[2], Barrie Wilkinson[3], Jacobus J. Boomsma[2], Matthew I Hutchings[1]

[1]School of Biological Sciences, University of East Anglia, Norwich Research Park, Norwich, Norfolk, United Kingdom.

[2]Centre for Social Evolution, University of Copenhagen, Copenhagen, Denmark.

[3]Department of Molecular Microbiology, John Innes Centre, Norwich Research Park, Norwich, Norfolk, United Kingdom.

[4]State Key Laboratory of Genetic Resources and Evolution, Kunming Institute of Zoology, Kunming, Yunnan, China.

[5]School of Environmental Sciences, University of East Anglia, Norwich Research Park, Norwich, Norfolk, United Kingdom.

Supplementary Information

Supplementary Table 1. Summary of draft genome sequences.

Sequencing Project	No. Unitigs	Largest Unitigs (bps)	Smallest Unitigs (bps)	No. of Preliminary Clusters (Antismash)
Ae150A_Ps1 Illumina	8	6135547	2072	15
Ae168_Ps1 Illumina	14	5473146	2073	15
Ae263_Ps1 Illumina	14	6157670	2073	15
Ae356_Ps1 Illumina	14	6162815	2206	15
Ae707_Ps1 PacBio	4	6361983	2594	14
Ae331_Ps2 Illumina	23	6075932	2005	11
Ae406_Ps2 Illumina	27	6080519	2001	11
Ae505_Ps2 Illumina	13	6031156	2260	13
Ae706_Ps2 Illumina	21	6111893	2111	11
Ae717_Ps2 PacBio	26	6288042	5131	15

Assembled genomes were submitted to antismash for secondary metabolite cluster prediction, outputs displayed below (Weber *et al.,* (2015).

Overview 1 2 3 4 5 6 7 8 9 10 11 12 13 14 15

Identified secondary metabolite clusters

Cluster	Type	From	To	Most similar known cluster
The following clusters are from record c00001_MH_Ae15:				
Cluster 1	T1pks	25302	96128	Nystatin-like_Pseudonocardia_polyene_biosynthetic_gene_cluster (73% of genes show similarity)
Cluster 2	Bacteriocin	279830	310643	Endophenazines_biosynthetic_gene_cluster (13% of genes show similarity)
Cluster 3	T1pks	691938	784056	Pimaricin_biosynthetic_gene_cluster (47% of genes show similarity)
Cluster 4	Terpene	949311	970576	SF2575_biosynthetic_gene_cluster (4% of genes show similarity)
Cluster 5	Nrps	1174990	1247881	Erythrochelin_biosynthetic_gene_cluster (71% of genes show similarity)
Cluster 6	Bacteriocin	2487311	2498171	-
Cluster 7	Other	2867409	2914879	Stenothricin_biosynthetic_gene_cluster (13% of genes show similarity)
Cluster 8	Nrps	2953650	3007679	Mirubactin_biosynthetic_gene_cluster (42% of genes show similarity)
Cluster 9	Ectoine	3090854	3101249	Ectoine_biosynthetic_gene_cluster (75% of genes show similarity)
Cluster 10	Oligosaccharide	3667360	3688043	-
Cluster 11	Other	3836118	3876684	Thiocoraline_biosynthetic_gene_cluster (5% of genes show similarity)
Cluster 12	Lantipeptide	4073882	4094031	-
Cluster 13	Other	5013321	5057700	Kinamycin_biosynthetic_gene_cluster (8% of genes show similarity)
Cluster 14	Nrps	5353829	5407642	Gobichelin_biosynthetic_gene_cluster (16% of genes show similarity)
Cluster 15	Terpene	5720239	5741237	Sioxanthin_biosynthetic_gene_cluster (60% of genes show similarity)

Supplementary Figure 1. Screen shot of the antismash output for *Pseudonocardia* Ae150A_Ps1.

Select Gene Cluster:
Overview 1 2 3 4 5 6 7 8 9 10 11 12 13 14 15

Identified secondary metabolite clusters

Cluster	Type	From	To	Most similar known cluster
The following clusters are from record c00001_MH_Ae16:				
Cluster 1	T1pks	29495	100321	Nystatin-like_Pseudonocardia_polyene_biosynthetic_gene_cluster (73% of genes show similarity)
Cluster 2	Bacteriocin	279174	309885	Endophenazines_biosynthetic_gene_cluster (13% of genes show similarity)
Cluster 3	T1pks	691404	783522	Pimaricin_biosynthetic_gene_cluster (47% of genes show similarity)
Cluster 4	Terpene	948624	969889	SF2575_biosynthetic_gene_cluster (4% of genes show similarity)
Cluster 5	Nrps	1174392	1247283	Erythrochelin_biosynthetic_gene_cluster (71% of genes show similarity)
Cluster 6	Bacteriocin	2485921	2496781	-
Cluster 7	Other	2866434	2913904	Stenothricin_biosynthetic_gene_cluster (13% of genes show similarity)
Cluster 8	Nrps	2952675	3006704	Mirubactin_biosynthetic_gene_cluster (42% of genes show similarity)
Cluster 9	Ectoine	3089879	3100274	Ectoine_biosynthetic_gene_cluster (75% of genes show similarity)
Cluster 10	Oligosaccharide	3670406	3691089	-
Cluster 11	Other	3839161	3879727	Thiocoraline_biosynthetic_gene_cluster (5% of genes show similarity)
Cluster 12	Lantipeptide	4076947	4097096	Kanamycin_biosynthetic_gene_cluster (1% of genes show similarity)
Cluster 13	Other	5026769	5071148	Kinamycin_biosynthetic_gene_cluster (8% of genes show similarity)
Cluster 14	Nrps	5367141	5420954	Gobichelin_biosynthetic_gene_cluster (16% of genes show similarity)
The following clusters are from record c00002_MH_Ae16:				
Cluster 15	Terpene	394356	415354	Sioxanthin_biosynthetic_gene_cluster (60% of genes show similarity)

Supplementary Figure 2. Screen shot of the antismash output for *Pseudonocardia* Ae168_Ps1.

Overview 1 2 3 4 5 6 7 8 9 10 11 12 13 14 15

Identified secondary metabolite clusters

Cluster	Type	From	To	Most similar known cluster
The following clusters are from record c00001_MH_Ae26:				
Cluster 1	Nrps	50728	104541	Gobichelin_biosynthetic_gene_cluster (22% of genes show similarity)
Cluster 2	Other	400667	445046	Kinamycin_biosynthetic_gene_cluster (8% of genes show similarity)
Cluster 3	Lantipeptide	1374731	1394880	-
Cluster 4	Other	1592018	1632584	Thiocoraline_biosynthetic_gene_cluster (5% of genes show similarity)
Cluster 5	Oligosaccharide	1780683	1801366	-
Cluster 6	Ectoine	2367786	2378181	Ectoine_biosynthetic_gene_cluster (75% of genes show similarity)
Cluster 7	Nrps	2465085	2519122	Mirubactin_biosynthetic_gene_cluster (42% of genes show similarity)
Cluster 8	Other	2557893	2605363	Stenothricin_biosynthetic_gene_cluster (13% of genes show similarity)
Cluster 9	Bacteriocin	2974454	2985314	-
Cluster 10	Nrps	4231090	4284904	Erythrochelin_biosynthetic_gene_cluster (71% of genes show similarity)
Cluster 11	Terpene	4508474	4529739	SF2575_biosynthetic_gene_cluster (6% of genes show similarity)
Cluster 12	T1pks	4694995	4783183	Nystatin-like_Pseudonocardia_polyene_biosynthetic_gene_cluster (43% of genes show similarity)
Cluster 13	Bacteriocin	5155393	5201206	Endophenazines_biosynthetic_gene_cluster (13% of genes show similarity)
Cluster 14	T1pks	5380061	5450887	Nystatin-like_Pseudonocardia_polyene_biosynthetic_gene_cluster (73% of genes show similarity)
Cluster 15	Terpene	5882622	5903620	Sioxanthin_biosynthetic_gene_cluster (60% of genes show similarity)

Supplementary Figure 3. Screen shot of the antismash output for *Pseudonocardia* Ae263_Ps1.

Select Gene Cluster:
Overview 1 2 3 4 5 6 7 8 9 10 11 12 13 14 15

Identified secondary metabolite clusters

Cluster	Type	From	To	Most similar known cluster
The following clusters are from record c00001_MH_Ae16:				
Cluster 1	T1pks	29495	100321	Nystatin-like_Pseudonocardia_polyene_biosynthetic_gene_cluster (73% of genes show similarity)
Cluster 2	Bacteriocin	279174	309885	Endophenazines_biosynthetic_gene_cluster (13% of genes show similarity)
Cluster 3	T1pks	691404	783522	Pimaricin_biosynthetic_gene_cluster (47% of genes show similarity)
Cluster 4	Terpene	948624	969889	SF2575_biosynthetic_gene_cluster (4% of genes show similarity)
Cluster 5	Nrps	1174392	1247283	Erythrochelin_biosynthetic_gene_cluster (71% of genes show similarity)
Cluster 6	Bacteriocin	2485921	2496781	-
Cluster 7	Other	2866434	2913904	Stenothricin_biosynthetic_gene_cluster (13% of genes show similarity)
Cluster 8	Nrps	2952675	3006704	Mirubactin_biosynthetic_gene_cluster (42% of genes show similarity)
Cluster 9	Ectoine	3089879	3100274	Ectoine_biosynthetic_gene_cluster (75% of genes show similarity)
Cluster 10	Oligosaccharide	3670406	3691089	-
Cluster 11	Other	3839161	3879727	Thiocoraline_biosynthetic_gene_cluster (5% of genes show similarity)
Cluster 12	Lantipeptide	4076947	4097096	Kanamycin_biosynthetic_gene_cluster (1% of genes show similarity)
Cluster 13	Other	5026769	5071148	Kinamycin_biosynthetic_gene_cluster (8% of genes show similarity)
Cluster 14	Nrps	5367141	5420954	Gobichelin_biosynthetic_gene_cluster (16% of genes show similarity)
The following clusters are from record c00002_MH_Ae16:				
Cluster 15	Terpene	394356	415354	Sioxanthin_biosynthetic_gene_cluster (60% of genes show similarity)

Supplementary Figure 4. Screen shot of the antismash output for *Pseudonocardia* Ae356_Ps1.

Select Gene Cluster:
Overview 1 2 3 4 5 6 7 8 9 10 11 12 13 14

Identified secondary metabolite clusters

Cluster	Type	From	To	Most similar known cluster
The following clusters are from record unitig_0quiver:				
Cluster 1	Oligosaccharide	56032	85438	-
Cluster 2	Oligosaccharide	188517	240149	Tomaymycin_biosynthetic_gene_cluster (11% of genes show similarity)
Cluster 3	Other	414511	455164	Thiocoraline_biosynthetic_gene_cluster (5% of genes show similarity)
Cluster 4	Nrps	1719125	1776380	Gobichelin_biosynthetic_gene_cluster (16% of genes show similarity)
Cluster 5	Terpene	2177040	2198059	Sioxanthin_biosynthetic_gene_cluster (60% of genes show similarity)
Cluster 6	Bacteriocin	2911608	2942494	Endophenazines_biosynthetic_gene_cluster (13% of genes show similarity)
Cluster 7	T1pks	3345783	3488407	Nystatin-like_Pseudonocardia_polyene_biosynthetic_gene_cluster (95% of genes show similarity)
Cluster 8	Terpene	3638728	3659975	SF2575_biosynthetic_gene_cluster (4% of genes show similarity)
Cluster 9	Nrps	3866837	3939727	Erythrochelin_biosynthetic_gene_cluster (71% of genes show similarity)
Cluster 10	Other	4710606	4754841	-
Cluster 11	Bacteriocin	5327392	5338333	-
Cluster 12	Other	5676793	5724917	Stenothricin_biosynthetic_gene_cluster (13% of genes show similarity)
Cluster 13	Nrps	5753305	5806262	Mirubactin_biosynthetic_gene_cluster (50% of genes show similarity)
Cluster 14	Ectoine	5865458	5875904	Ectoine_biosynthetic_gene_cluster (75% of genes show similarity)

Supplementary Figure 5. Screen shot of the antismash output for *Pseudonocardia* Ae707_Ps1.

Select Gene Cluster:
Overview 1 2 3 4 5 6 7 8 9 10 11

Identified secondary metabolite clusters

Cluster	Type	From	To	Most similar known cluster
The following clusters are from record c00001_MH_Ae33:				
Cluster 1	Terpene	781011	802687	Brasilicardin_A_biosynthetic_gene_cluster (45% of genes show similarity)
Cluster 2	Bacteriocin	1174490	1185383	-
Cluster 3	Ectoine	1666066	1676461	Ectoine_biosynthetic_gene_cluster (75% of genes show similarity)
Cluster 4	Oligosaccharide	2231888	2252565	-
Cluster 5	Bacteriocin	3612149	3622970	-
Cluster 6	Terpene	4081263	4102255	Carotenoid_biosynthetic_gene_cluster (27% of genes show similarity)
Cluster 7	T1pks	4380747	4519777	Nystatin-like_Pseudonocardia_polyene_biosynthetic_gene_cluster (73% of genes show similarity)
Cluster 8	Nrps-T1pks	4721587	4863642	Amychelin_biosynthetic_gene_cluster (31% of genes show similarity)
Cluster 9	Terpene	5326209	5347564	SF2575_biosynthetic_gene_cluster (4% of genes show similarity)
Cluster 10	Other	6006054	6070086	UK-68,597_biosynthetic_gene_cluster (4% of genes show similarity)
The following clusters are from record c00002_MH_Ae33:				
Cluster 11	Lassopeptide	89260	111169	-

Supplementary Figure 6. Screen shot of the antismash output for *Pseudonocardia* Ae331_Ps2.

Select Gene Cluster:
Overview 1 2 3 4 5 6 7 8 9 10 11

Identified secondary metabolite clusters

Cluster	Type	From	To	Most similar known cluster
The following clusters are from record c00001_MH_Ae40:				
Cluster 1	Other	5853	69885	UK-68,597_biosynthetic_gene_cluster (4% of genes show similarity)
Cluster 2	Terpene	730605	751954	SF2575_biosynthetic_gene_cluster (6% of genes show similarity)
Cluster 3	Nrps-T1pks	1213868	1354303	Amychelin_biosynthetic_gene_cluster (31% of genes show similarity)
Cluster 4	T1pks	1554210	1688401	Nystatin-like_Pseudonocardia_polyene_biosynthetic_gene_cluster (73% of genes show similarity)
Cluster 5	Terpene	1982209	2003201	Carotenoid_biosynthetic_gene_cluster (27% of genes show similarity)
Cluster 6	Bacteriocin	2457460	2468281	-
Cluster 7	Oligosaccharide	3825163	3845840	-
Cluster 8	Ectoine	4409093	4419488	Ectoine_biosynthetic_gene_cluster (100% of genes show similarity)
Cluster 9	Bacteriocin	4897074	4907967	-
Cluster 10	Terpene	5281504	5303180	Brasilicardin_A_biosynthetic_gene_cluster (54% of genes show similarity)
The following clusters are from record c00002_MH_Ae40:				
Cluster 11	Lassopeptide	92023	113932	-

Supplementary Figure 7. Screen shot of the antismash output for *Pseudonocardia* Ae406_Ps2.

Select Gene Cluster:
Overview 1 2 3 4 5 6 7 8 9 10 11 12 13

Identified secondary metabolite clusters

Cluster	Type	From	To	Most similar known cluster
The following clusters are from record c00001_MH_Ae50:				
Cluster 1	Terpene	153971	175647	Brasilicardin_A_biosynthetic_gene_cluster (45% of genes show similarity)
Cluster 2	Bacteriocin	545698	556591	-
Cluster 3	Ectoine	1027041	1037436	Ectoine_biosynthetic_gene_cluster (75% of genes show similarity)
Cluster 4	Oligosaccharide	1605297	1625992	-
Cluster 5	Bacteriocin	3018924	3029745	-
Cluster 6	Terpene	3264901	3285893	Isorenieratene_biosynthetic_gene_cluster (28% of genes show similarity)
Cluster 7	Other	3697921	3761893	UK-68,597_biosynthetic_gene_cluster (4% of genes show similarity)
Cluster 8	T1pks	4197361	4258545	Macbecin_biosynthetic_gene_cluster (39% of genes show similarity)
Cluster 9	Nrps	4302433	4362588	Kiamycin_biosynthetic_gene_cluster (10% of genes show similarity)
Cluster 10	T1pks-Nrps	4356144	4461507	Amychelin_biosynthetic_gene_cluster (31% of genes show similarity)
Cluster 11	Terpene	4926042	4947361	SF2575_biosynthetic_gene_cluster (4% of genes show similarity)
Cluster 12	T1pks	5232700	5300963	Nystatin-like_Pseudonocardia_polyene_biosynthetic_gene_cluster (47% of genes show similarity)
The following clusters are from record c00002_MH_Ae50:				
Cluster 13	Lassopeptide	101953	123862	-

Supplementary Figure 8. Screen shot of the antismash output for *Pseudonocardia* Ae505_Ps2.

Select Gene Cluster:
Overview 1 2 3 4 5 6 7 8 9 10 11

Identified secondary metabolite clusters

Cluster	Type	From	To	Most similar known cluster
The following clusters are from record c00001_MH_Ae70:				
Cluster 1	Other	5853	69885	UK-68,597_biosynthetic_gene_cluster (4% of genes show similarity)
Cluster 2	Terpene	740562	761911	SF2575_biosynthetic_gene_cluster (6% of genes show similarity)
Cluster 3	Nrps-T1pks	1224015	1363220	Amychelin_biosynthetic_gene_cluster (31% of genes show similarity)
Cluster 4	T1pks	1563134	1702164	Nystatin-like_Pseudonocardia_polyene_biosynthetic_gene_cluster (73% of genes show similarity)
Cluster 5	Terpene	1992113	2013105	Carotenoid_biosynthetic_gene_cluster (27% of genes show similarity)
Cluster 6	Bacteriocin	2468953	2479774	-
Cluster 7	Oligosaccharide	3835997	3856674	-
Cluster 8	Ectoine	4424378	4434773	Ectoine_biosynthetic_gene_cluster (100% of genes show similarity)
Cluster 9	Bacteriocin	4915836	4926729	-
Cluster 10	Terpene	5302849	5324525	Brasilicardin_A_biosynthetic_gene_cluster (54% of genes show similarity)
The following clusters are from record c00002_MH_Ae70:				
Cluster 11	Lassopeptide	111865	133774	-

Supplementary Figure 9. Screen shot of the antismash output for *Pseudonocardia* Ae706_Ps2.

Select Gene Cluster:
Overview 1 2 3 4 5 6 7 8 9 10 11 12 13 14 15

Identified secondary metabolite clusters

Cluster	Type	From	To	Most similar known cluster
The following clusters are from record unitig_0quiver:				
Cluster 1	Otherks	766234	807433	Galbonolides_biosynthetic_gene_cluster (16% of genes show similarity)
Cluster 2	Bacteriocin	1131133	1142233	-
Cluster 3	Other	1654291	1718271	UK-68,597_biosynthetic_gene_cluster (4% of genes show similarity)
Cluster 4	T1pks	2222655	2283874	Macbecin_biosynthetic_gene_cluster (47% of genes show similarity)
Cluster 5	Nrps	2326835	2386990	Kiamycin_biosynthetic_gene_cluster (10% of genes show similarity)
Cluster 6	T1pks-Nrps	2387695	2494012	Amychelin_biosynthetic_gene_cluster (31% of genes show similarity)
Cluster 7	Terpene	2956317	2977720	SF2575_biosynthetic_gene_cluster (4% of genes show similarity)
Cluster 8	T1pks	3259748	3397832	Nystatin-like_Pseudonocardia_polyene_biosynthetic_gene_cluster (73% of genes show similarity)
Cluster 9	Terpene	3688660	3709652	Isorenieratene_biosynthetic_gene_cluster (28% of genes show similarity)
Cluster 10	Terpene	4500688	4522868	Brasilicardin_A_biosynthetic_gene_cluster (45% of genes show similarity)
Cluster 11	Bacteriocin	4901917	4912810	-
Cluster 12	Ectoine	5387849	5398496	Ectoine_biosynthetic_gene_cluster (75% of genes show similarity)
Cluster 13	Oligosaccharide	5947675	5977172	-
The following clusters are from record unitig_6quiver:				
Cluster 14	Lassopeptide	60885	83439	-
The following clusters are from record unitig_17quiver:				
Cluster 15	T2pks	32985	76304	Arimetamycin_biosynthetic_gene_cluster (21% of genes show similarity)

Supplementary Figure 10. Screen shot of the antismash output for *Pseudonocardia* Ae717_Ps2.

Clustal 2.1 (Larkin *et al.,* 2007) was used to align the 16S rRNA gene, the *rpsL* gene and the *rpoB* gene from different *Pseudonocardia* strains.

Pseudonocardia 16S rRNA gene alignment;

```
Ae706Ps2_16S        AGAGTTTGATCCTGGCTCAGGACGAACGCTGGCGGCGTGCTTAACACATGCAAGTCGAGC
Ae717Ps2_16S        AGAGTTTGATCCTGGCTCAGGACGAACGCTGGCGGCGTGCTTAACACATGCAAGTCGAGC
Ae505Ps2_16S        AGAGTTTGATCCTGGCTCAGGACGAACGCTGGCGGCGTGCTTAACACATGCAAGTCGAGC
Ae406Ps2_16S        AGAGTTTGATCCTGGCTCAGGACGAACGCTGGCGGCGTGCTTAACACATGCAAGTCGAGC
Ae331Ps2_16S        AGAGTTTGATCCTGGCTCAGGACGAACGCTGGCGGCGTGCTTAACACATGCAAGTCGAGC
Ae150APs1_16S       AGAGTTTGATCCTGGCTCAGGACGAACGCTGGCGGCGTGCTTAACACATGCAAGTCGAGC
Ae707Ps1_16S        AGAGTTTGATCCTGGCTCAGGACGAACGCTGGCGGCGTGCTTAACACATGCAAGTCGAGC
Ae168Ps1_16S        AGAGTTTGATCCTGGCTCAGGACGAACGCTGGCGGCGTGCTTAACACATGCAAGTCGAGC
```

```
Ae263Ps1_16S      AGAGTTTGATCCTGGCTCAGGACGAACGCTGGCGGCGTGCTTAACACATGCAAGTCGAGC
Ae356Ps1_16S      AGAGTTTGATCCTGGCTCAGGACGAACGCTGGCGGCGTGCTTAACACATGCAAGTCGAGC
                  ************************************************************

Ae706Ps2_16S      GGTAAGGCCCTTTCGGGGGTACACGAGCGGCGAACGGGTGAGTAACACGTGGGTGACCTG
Ae717Ps2_16S      GGTAAGGCCCTTTCGGGGGTACACGAGCGGCGAACGGGTGAGTAACACGTGGGTGACCTG
Ae505Ps2_16S      GGTAAGGCCCTTTCGGGGGTACACGAGCGGCGAACGGGTGAGTAACACGTGGGTGACCTG
Ae406Ps2_16S      GGTAAGGCCCTTTCGGGGGTACACGAGCGGCGAACGGGTGAGTAACACGTGGGTGACCTG
Ae331Ps2_16S      GGTAAGGCCCTTTCGGGGGTACACGAGCGGCGAACGGGTGAGTAACACGTGGGTGACCTG
Ae150APs1_16S     GGTAAGGCCCTTTCGGGGGTACACGAGCGGCGAACGGGTGAGTAACACGTGGGTGACCTG
Ae707Ps1_16S      GGTAAGGCCCTTTCGGGGGTACACGAGCGGCGAACGGGTGAGTAACACGTGGGTGACCTG
Ae168Ps1_16S      GGTAAGGCCCTTTCGGGGGTACACGAGCGGCGAACGGGTGAGTAACACGTGGGTGACCTG
Ae263Ps1_16S      GGTAAGGCCCTTTCGGGGGTACACGAGCGGCGAACGGGTGAGTAACACGTGGGTGACCTG
Ae356Ps1_16S      GGTAAGGCCCTTTCGGGGGTACACGAGCGGCGAACGGGTGAGTAACACGTGGGTGACCTG
                  ************************************************************

Ae706Ps2_16S      CCCTCCACTCTGGGATAAGCCTGGGAAACTGGGTCTAATACCGGATAGGACCACTGGCTG
Ae717Ps2_16S      CCCTCCACTCTGGGATAAGCCTGGGAAACTGGGTCTAATACCGGATAGGACCACTGGCTG
Ae505Ps2_16S      CCCTCCACTCTGGGATAAGCCTGGGAAACTGGGTCTAATACCGGATAGGACCACTGGCTG
Ae406Ps2_16S      CCCTCCACTCTGGGATAAGCCTGGGAAACTGGGTCTAATACCGGATAGGACCACTGGCTG
Ae331Ps2_16S      CCCTCCACTCTGGGATAAGCCTGGGAAACTGGGTCTAATACCGGATAGGACCACTGGCTG
Ae150APs1_16S     CCCTCCACTCTGGGATAAGCCCGGGAAACTGGGTCTAATACCGGATAGGACCTCTCAACG
Ae707Ps1_16S      CCCTCCACTCTGGGATAAGCCCGGGAAACTGGGTCTAATACCGGATAGGACCTCTCAACG
Ae168Ps1_16S      CCCTCCACTCTGGGATAAGCCCGGGAAACTGGGTCTAATACCGGATAGGACCTCTCAACG
Ae263Ps1_16S      CCCTCCACTCTGGGATAAGCCCGGGAAACTGGGTCTAATACCGGATAGGACCTCTCAACG
Ae356Ps1_16S      CCCTCCACTCTGGGATAAGCCCGGGAAACTGGGTCTAATACCGGATAGGACCTCTCAACG
                  ********************* ****************************** **    *

Ae706Ps2_16S      CATGGTTGGTGGTGGAAAGTTTTTT-CGGTGGGGGATGGGCCCGCGGCCTATCAGCTTGT
Ae717Ps2_16S      CATGGTTGGTGGTGGAAAGTTTTTTTCGGTGGGGGATGGGCCCGCGGCCTATCAGCTTGT
Ae505Ps2_16S      CATGGTTGGTGGTGGAAAGTTTTTT-CGGTGGGGGATGGGCCCGCGGCCTATCAGCTTGT
Ae406Ps2_16S      CATGGTTGGTGGTGGAAAGTTTTTT-CGGTGGGGGATGGGCCCGCGGCCTATCAGCTTGT
Ae331Ps2_16S      CATGGTTGGTGGTGGAAAGTTTTTT-CGGTGGGGGATGGGCCCGCGGCCTATCAGCTTGT
Ae150APs1_16S     CATGTTGGGTGGTGGAAAGTTTTTT-CGGTGGGGGATGGGCCCGCGGCCTATCAGCTTGT
Ae707Ps1_16S      CATGTTGGGTGGTGGAAAGTTTTTT-CGGTGGGGGATGGGCCCGCGGCCTATCAGCTTGT
Ae168Ps1_16S      CATGTTGGGTGGTGGAAAGTTTTTT-CGGTGGGGGATGGGCCCGCGGCCTATCAGCTTGT
Ae263Ps1_16S      CATGTTGGGTGGTGGAAAGTTTTTT-CGGTGGGGGATGGGCCCGCGGCCTATCAGCTTGT
Ae356Ps1_16S      CATGTTGGGTGGTGGAAAGTTTTTT-CGGTGGGGGATGGGCCCGCGGCCTATCAGCTTGT
                  **** * ****************** **********************************

Ae706Ps2_16S      TGGTGGGGTGATGGCCTACCAAGGCGGTGACGGGTAGCCGGCCTGAGAGGGCGACCGGCC
Ae717Ps2_16S      TGGTGGGGTGATGGCCTACCAAGGCGGTGACGGGTAGCCGGCCTGAGAGGGCGACCGGCC
Ae505Ps2_16S      TGGTGGGGTGATGGCCTACCAAGGCGGTGACGGGTAGCCGGCCTGAGAGGGCGACCGGCC
Ae406Ps2_16S      TGGTGGGGTGATGGCCTACCAAGGCGGTGACGGGTAGCCGGCCTGAGAGGGCGACCGGCC
Ae331Ps2_16S      TGGTGGGGTGATGGCCTACCAAGGCGGTGACGGGTAGCCGGCCTGAGAGGGCGACCGGCC
Ae150APs1_16S     TGGTGGGGTGATGGCCTACCAAGGCGGTGACGGGTAGCCGGCCTGAGAGGGCGACCGGCC
Ae707Ps1_16S      TGGTGGGGTGATGGCCTACCAAGGCGGTGACGGGTAGCCGGCCTGAGAGGGCGACCGGCC
Ae168Ps1_16S      TGGTGGGGTGATGGCCTACCAAGGCGGTGACGGGTAGCCGGCCTGAGAGGGCGACCGGCC
Ae263Ps1_16S      TGGTGGGGTGATGGCCTACCAAGGCGGTGACGGGTAGCCGGCCTGAGAGGGCGACCGGCC
Ae356Ps1_16S      TGGTGGGGTGATGGCCTACCAAGGCGGTGACGGGTAGCCGGCCTGAGAGGGCGACCGGCC
                  ************************************************************

Ae706Ps2_16S      ACACTGGGACTGAGACACGGCCCAGACTCCTACGGGAGGCAGCAGTGGGGAATATTGCGC
Ae717Ps2_16S      ACACTGGGACTGAGACACGGCCCAGACTCCTACGGGAGGCAGCAGTGGGGAATATTGCGC
Ae505Ps2_16S      ACACTGGGACTGAGACACGGCCCAGACTCCTACGGGAGGCAGCAGTGGGGAATATTGCGC
Ae406Ps2_16S      ACACTGGGACTGAGACACGGCCCAGACTCCTACGGGAGGCAGCAGTGGGGAATATTGCGC
Ae331Ps2_16S      ACACTGGGACTGAGACACGGCCCAGACTCCTACGGGAGGCAGCAGTGGGGAATATTGCGC
Ae150APs1_16S     ACACTGGGACTGAGACACGGCCCAGACTCCTACGGGAGGCAGCAGTGGGGAATATTGCGC
Ae707Ps1_16S      ACACTGGGACTGAGACACGGCCCAGACTCCTACGGGAGGCAGCAGTGGGGAATATTGCGC
Ae168Ps1_16S      ACACTGGGACTGAGACACGGCCCAGACTCCTACGGGAGGCAGCAGTGGGGAATATTGCGC
Ae263Ps1_16S      ACACTGGGACTGAGACACGGCCCAGACTCCTACGGGAGGCAGCAGTGGGGAATATTGCGC
Ae356Ps1_16S      ACACTGGGACTGAGACACGGCCCAGACTCCTACGGGAGGCAGCAGTGGGGAATATTGCGC
                  ************************************************************

Ae706Ps2_16S      AATGGGCGGAAGCCTGACGCAGCGACGCCGCGTGGGGGATGACGGCCTTCGGGTTGTAAA
Ae717Ps2_16S      AATGGGCGGAAGCCTGACGCAGCGACGCCGCGTGGGGGATGACGGCCTTCGGGTTGTAAA
Ae505Ps2_16S      AATGGGCGGAAGCCTGACGCAGCGACGCCGCGTGGGGGATGACGGCCTTCGGGTTGTAAA
Ae406Ps2_16S      AATGGGCGGAAGCCTGACGCAGCGACGCCGCGTGGGGGATGACGGCCTTCGGGTTGTAAA
Ae331Ps2_16S      AATGGGCGGAAGCCTGACGCAGCGACGCCGCGTGGGGGATGACGGCCTTCGGGTTGTAAA
Ae150APs1_16S     AATGGGCGGAAGCCTGACGCAGCGACGCCGCGTGGGGGATGACGGCCTTCGGGTTGTAAA
Ae707Ps1_16S      AATGGGCGGAAGCCTGACGCAGCGACGCCGCGTGGGGGATGACGGCCTTCGGGTTGTAAA
Ae168Ps1_16S      AATGGGCGGAAGCCTGACGCAGCGACGCCGCGTGGGGGATGACGGCCTTCGGGTTGTAAA
Ae263Ps1_16S      AATGGGCGGAAGCCTGACGCAGCGACGCCGCGTGGGGGATGACGGCCTTCGGGTTGTAAA
Ae356Ps1_16S      AATGGGCGGAAGCCTGACGCAGCGACGCCGCGTGGGGGATGACGGCCTTCGGGTTGTAAA
                  ************************************************************

Ae706Ps2_16S      CCTCTTTCGCCAGGGACGAAGAGTGATTGACGGTACCTGGAGAAGAAGCACCGGCCAACT
```

```
Ae717Ps2_16S       CCTCTTTCGCCAGGGACGAAGAGTGATTGACGGTACCTGGAGAAGAAGCACCGGCCAACT
Ae505Ps2_16S       CCTCTTTCGCCAGGGACGAAGAGTGATTGACGGTACCTGGAGAAGAAGCACCGGCCAACT
Ae406Ps2_16S       CCTCTTTCGCCAGGGACGAAGAGTGATTGACGGTACCTGGAGAAGAAGCACCGGCCAACT
Ae331Ps2_16S       CCTCTTTCGCCAGGGACGAAGAGTGATTGACGGTACCTGGAGAAGAAGCACCGGCCAACT
Ae150APs1_16S      CCTCTTTCGCCAGGGACGAAGCTTTTGTGACGGTACCTGGAGAAGAAGCACCGGCCAACT
Ae707Ps1_16S       CCTCTTTCGCCAGGGACGAAGCTTTTGTGACGGTACCTGGAGAAGAAGCACCGGCCAACT
Ae168Ps1_16S       CCTCTTTCGCCAGGGACGAAGCTTTTGTGACGGTACCTGGAGAAGAAGCACCGGCCAACT
Ae263Ps1_16S       CCTCTTTCGCCAGGGACGAAGCTTTTGTGACGGTACCTGGAGAAGAAGCACCGGCCAACT
Ae356Ps1_16S       CCTCTTTCGCCAGGGACGAAGCTTTTGTGACGGTACCTGGAGAAGAAGCACCGGCCAACT
                   ********************   *   *********************************

Ae706Ps2_16S       ACGTGCCAGCAGCCGCGGTAACACGTAGGGTGCGAGCGTTGTCCGGAATTATTGGGCGTA
Ae717Ps2_16S       ACGTGCCAGCAGCCGCGGTAACACGTAGGGTGCGAGCGTTGTCCGGAATTATTGGGCGTA
Ae505Ps2_16S       ACGTGCCAGCAGCCGCGGTAACACGTAGGGTGCGAGCGTTGTCCGGAATTATTGGGCGTA
Ae406Ps2_16S       ACGTGCCAGCAGCCGCGGTAACACGTAGGGTGCGAGCGTTGTCCGGAATTATTGGGCGTA
Ae331Ps2_16S       ACGTGCCAGCAGCCGCGGTAACACGTAGGGTGCGAGCGTTGTCCGGAATTATTGGGCGTA
Ae150APs1_16S      ACGTGCCAGCAGCCGCGGTAACACGTAGGGTGCGAGCGTTGTCCGGAATTATTGGGCGTA
Ae707Ps1_16S       ACGTGCCAGCAGCCGCGGTAACACGTAGGGTGCGAGCGTTGTCCGGAATTATTGGGCGTA
Ae168Ps1_16S       ACGTGCCAGCAGCCGCGGTAACACGTAGGGTGCGAGCGTTGTCCGGAATTATTGGGCGTA
Ae263Ps1_16S       ACGTGCCAGCAGCCGCGGTAACACGTAGGGTGCGAGCGTTGTCCGGAATTATTGGGCGTA
Ae356Ps1_16S       ACGTGCCAGCAGCCGCGGTAACACGTAGGGTGCGAGCGTTGTCCGGAATTATTGGGCGTA
                   ************************************************************

Ae706Ps2_16S       AAGAGCTCGTAGGCGGTGTGTCGCGTCGGCCGTGAAAACTTGGGGCTTAACTCTGAGCGT
Ae717Ps2_16S       AAGAGCTCGTAGGCGGTGTGTCGCGTCGGCCGTGAAAACTTGGGGCTTAACTCTGAGCGT
Ae505Ps2_16S       AAGAGCTCGTAGGCGGTGTGTCGCGTCGGCCGTGAAAACTTGGGGCTTAACTCTGAGCGT
Ae406Ps2_16S       AAGAGCTCGTAGGCGGTGTGTCGCGTCGGCCGTGAAAACTTGGGGCTTAACTCTGAGCGT
Ae331Ps2_16S       AAGAGCTCGTAGGCGGTGTGTCGCGTCGGCCGTGAAAACTTGGGGCTTAACTCTGAGCGT
Ae150APs1_16S      AAGAGCTCGTAGGCGGTGTGTCGCGTCGGCCGTGAAAACTTGGGGCTTAACTCTGAGCGT
Ae707Ps1_16S       AAGAGCTCGTAGGCGGTGTGTCGCGTCGGCCGTGAAAACTTGGGGCTTAACTCTGAGCGT
Ae168Ps1_16S       AAGAGCTCGTAGGCGGTGTGTCGCGTCGGCCGTGAAAACTTGGGGCTTAACTCTGAGCGT
Ae263Ps1_16S       AAGAGCTCGTAGGCGGTGTGTCGCGTCGGCCGTGAAAACTTGGGGCTTAACTCTGAGCGT
Ae356Ps1_16S       AAGAGCTCGTAGGCGGTGTGTCGCGTCGGCCGTGAAAACTTGGGGCTTAACTCTGAGCGT
                   ************************************************************

Ae706Ps2_16S       GCGGTCGATACGGGCATCACTTGAGTTCGGCAGGGGAGACTGGAATTCCTGGTGTAGCGG
Ae717Ps2_16S       GCGGTCGATACGGGCATCACTTGAGTTCGGCAGGGGAGACTGGAATTCCTGGTGTAGCGG
Ae505Ps2_16S       GCGGTCGATACGGGCATCACTTGAGTTCGGCAGGGGAGACTGGAATTCCTGGTGTAGCGG
Ae406Ps2_16S       GCGGTCGATACGGGCATCACTTGAGTTCGGCAGGGGAGACTGGAATTCCTGGTGTAGCGG
Ae331Ps2_16S       GCGGTCGATACGGGCATCACTTGAGTTCGGCAGGGGAGACTGGAATTCCTGGTGTAGCGG
Ae150APs1_16S      GCGGTCGATACGGGCATCACTTGAGTTCGGCAGGGGAGACTGGAATTCCTGGTGTAGCGG
Ae707Ps1_16S       GCGGTCGATACGGGCATCACTTGAGTTCGGCAGGGGAGACTGGAATTCCTGGTGTAGCGG
Ae168Ps1_16S       GCGGTCGATACGGGCATCACTTGAGTTCGGCAGGGGAGACTGGAATTCCTGGTGTAGCGG
Ae263Ps1_16S       GCGGTCGATACGGGCATCACTTGAGTTCGGCAGGGGAGACTGGAATTCCTGGTGTAGCGG
Ae356Ps1_16S       GCGGTCGATACGGGCATCACTTGAGTTCGGCAGGGGAGACTGGAATTCCTGGTGTAGCGG
                   ************************************************************

Ae706Ps2_16S       TGAAATGCGCAGATATCAGGAGGAACACCGGTGGCGAAGGCGGGTCTCTGGGCCGATACT
Ae717Ps2_16S       TGAAATGCGCAGATATCAGGAGGAACACCGGTGGCGAAGGCGGGTCTCTGGGCCGATACT
Ae505Ps2_16S       TGAAATGCGCAGATATCAGGAGGAACACCGGTGGCGAAGGCGGGTCTCTGGGCCGATACT
Ae406Ps2_16S       TGAAATGCGCAGATATCAGGAGGAACACCGGTGGCGAAGGCGGGTCTCTGGGCCGATACT
Ae331Ps2_16S       TGAAATGCGCAGATATCAGGAGGAACACCGGTGGCGAAGGCGGGTCTCTGGGCCGATACT
Ae150APs1_16S      TGAAATGCGCAGATATCAGGAGGAACACCGGTGGCGAAGGCGGGTCTCTGGGCCGATACT
Ae707Ps1_16S       TGAAATGCGCAGATATCAGGAGGAACACCGGTGGCGAAGGCGGGTCTCTGGGCCGATACT
Ae168Ps1_16S       TGAAATGCGCAGATATCAGGAGGAACACCGGTGGCGAAGGCGGGTCTCTGGGCCGATACT
Ae263Ps1_16S       TGAAATGCGCAGATATCAGGAGGAACACCGGTGGCGAAGGCGGGTCTCTGGGCCGATACT
Ae356Ps1_16S       TGAAATGCGCAGATATCAGGAGGAACACCGGTGGCGAAGGCGGGTCTCTGGGCCGATACT
                   ************************************************************

Ae706Ps2_16S       GACGCTGAGGAGCGAAAGCGTGGGGAGCGAACAGGATTAGATACCCTGGTAGTCCACGCC
Ae717Ps2_16S       GACGCTGAGGAGCGAAAGCGTGGGGAGCGAACAGGATTAGATACCCTGGTAGTCCACGCC
Ae505Ps2_16S       GACGCTGAGGAGCGAAAGCGTGGGGAGCGAACAGGATTAGATACCCTGGTAGTCCACGCC
Ae406Ps2_16S       GACGCTGAGGAGCGAAAGCGTGGGGAGCGAACAGGATTAGATACCCTGGTAGTCCACGCC
Ae331Ps2_16S       GACGCTGAGGAGCGAAAGCGTGGGGAGCGAACAGGATTAGATACCCTGGTAGTCCACGCC
Ae150APs1_16S      GACGCTGAGGAGCGAAAGCGTGGGGAGCGAACAGGATTAGATACCCTGGTAGTCCACGCC
Ae707Ps1_16S       GACGCTGAGGAGCGAAAGCGTGGGGAGCGAACAGGATTAGATACCCTGGTAGTCCACGCC
Ae168Ps1_16S       GACGCTGAGGAGCGAAAGCGTGGGGAGCGAACAGGATTAGATACCCTGGTAGTCCACGCC
Ae263Ps1_16S       GACGCTGAGGAGCGAAAGCGTGGGGAGCGAACAGGATTAGATACCCTGGTAGTCCACGCC
Ae356Ps1_16S       GACGCTGAGGAGCGAAAGCGTGGGGAGCGAACAGGATTAGATACCCTGGTAGTCCACGCC
                   ************************************************************

Ae706Ps2_16S       GTAAACGTTGGGCGCTAGGTGTGGGGACCATTCCACGGTTTCTGTGCCGCAGCTAACGCA
Ae717Ps2_16S       GTAAACGTTGGGCGCTAGGTGTGGGGACCATTCCACGGTTTCTGTGCCGCAGCTAACGCA
Ae505Ps2_16S       GTAAACGTTGGGCGCTAGGTGTGGGGACCATTCCACGGTTTCTGTGCCGCAGCTAACGCA
Ae406Ps2_16S       GTAAACGTTGGGCGCTAGGTGTGGGGACCATTCCACGGTTTCTGTGCCGCAGCTAACGCA
Ae331Ps2_16S       GTAAACGTTGGGCGCTAGGTGTGGGGACCATTCCACGGTTTCTGTGCCGCAGCTAACGCA
Ae150APs1_16S      GTAAACGTTGGGCGCTAGGTGTGGGGACCATTCCACGGTTTCTGCGCCGCAGCTAACGCA
```

```
Ae707Ps1_16S       GTAAACGTTGGGCGCTAGGTGTGGGGACCATTCCACGGTTTCTGCGCCGCAGCTAACGCA
Ae168Ps1_16S       GTAAACGTTGGGCGCTAGGTGTGGGGACCATTCCACGGTTTCTGCGCCGCAGCTAACGCA
Ae263Ps1_16S       GTAAACGTTGGGCGCTAGGTGTGGGGACCATTCCACGGTTTCTGCGCCGCAGCTAACGCA
Ae356Ps1_16S       GTAAACGTTGGGCGCTAGGTGTGGGGACCATTCCACGGTTTCTGCGCCGCAGCTAACGCA
                   ******************************************** ***************

Ae706Ps2_16S       TTAAGCGCCCCGCCTGGGGAGTACGGCCGCAAGGCTAAAACTCAAAGGAATTGACGGGGG
Ae717Ps2_16S       TTAAGCGCCCCGCCTGGGGAGTACGGCCGCAAGGCTAAAACTCAAAGGAATTGACGGGGG
Ae505Ps2_16S       TTAAGCGCCCCGCCTGGGGAGTACGGCCGCAAGGCTAAAACTCAAAGGAATTGACGGGGG
Ae406Ps2_16S       TTAAGCGCCCCGCCTGGGGAGTACGGCCGCAAGGCTAAAACTCAAAGGAATTGACGGGGG
Ae331Ps2_16S       TTAAGCGCCCCGCCTGGGGAGTACGGCCGCAAGGCTAAAACTCAAAGGAATTGACGGGGG
Ae150APs1_16S      TTAAGCGCCCCGCCTGGGGAGTACGGCCGCAAGGCTAAAACTCAAAGGAATTGACGGGGG
Ae707Ps1_16S       TTAAGCGCCCCGCCTGGGGAGTACGGCCGCAAGGCTAAAACTCAAAGGAATTGACGGGGG
Ae168Ps1_16S       TTAAGCGCCCCGCCTGGGGAGTACGGCCGCAAGGCTAAAACTCAAAGGAATTGACGGGGG
Ae263Ps1_16S       TTAAGCGCCCCGCCTGGGGAGTACGGCCGCAAGGCTAAAACTCAAAGGAATTGACGGGGG
Ae356Ps1_16S       TTAAGCGCCCCGCCTGGGGAGTACGGCCGCAAGGCTAAAACTCAAAGGAATTGACGGGGG
                   ************************************************************

Ae706Ps2_16S       CCCGCACAAGCGGCGGAGCATGTGGATTAATTCGATGCAACGCGAAGAACCTTACCTGGG
Ae717Ps2_16S       CCCGCACAAGCGGCGGAGCATGTGGATTAATTCGATGCAACGCGAAGAACCTTACCTGGG
Ae505Ps2_16S       CCCGCACAAGCGGCGGAGCATGTGGATTAATTCGATGCAACGCGAAGAACCTTACCTGGG
Ae406Ps2_16S       CCCGCACAAGCGGCGGAGCATGTGGATTAATTCGATGCAACGCGAAGAACCTTACCTGGG
Ae331Ps2_16S       CCCGCACAAGCGGCGGAGCATGTGGATTAATTCGATGCAACGCGAAGAACCTTACCTGGG
Ae150APs1_16S      CCCGCACAAGCGGCGGAGCATGTGGATTAATTCGATGCAACGCGAAGAACCTTACCTGGG
Ae707Ps1_16S       CCCGCACAAGCGGCGGAGCATGTGGATTAATTCGATGCAACGCGAAGAACCTTACCTGGG
Ae168Ps1_16S       CCCGCACAAGCGGCGGAGCATGTGGATTAATTCGATGCAACGCGAAGAACCTTACCTGGG
Ae263Ps1_16S       CCCGCACAAGCGGCGGAGCATGTGGATTAATTCGATGCAACGCGAAGAACCTTACCTGGG
Ae356Ps1_16S       CCCGCACAAGCGGCGGAGCATGTGGATTAATTCGATGCAACGCGAAGAACCTTACCTGGG
                   ************************************************************

Ae706Ps2_16S       TTTGACATGCACCAGACATCCCTAGAGATAGGGCTTCCCTTGTGGTTGGTGTGCAGGTGG
Ae717Ps2_16S       TTTGACATGCACCAGACATCCCTAGAGATAGGGCTTCCCTTGTGGTTGGTGTGCAGGTGG
Ae505Ps2_16S       TTTGACATGCACCAGACATCCCTAGAGATAGGGCTTCCCTTGTGGTTGGTGTGCAGGTGG
Ae406Ps2_16S       TTTGACATGCACCAGACATCCCTAGAGATAGGGCTTCCCTTGTGGTTGGTGTGCAGGTGG
Ae331Ps2_16S       TTTGACATGCACCAGACATCCCTAGAGATAGGGCTTCCCTTGTGGTTGGTGTGCAGGTGG
Ae150APs1_16S      TTTGACATGCACAGGATCGCGGCAGAGATGTCGTTTCCCTTGTGGCCTGTGTGCAGGTGG
Ae707Ps1_16S       TTTGACATGCACAGGATCGCGGCAGAGATGTCGTTTCCCTTGTGGCCTGTGTGCAGGTGG
Ae168Ps1_16S       TTTGACATGCACAGGATCGCGGCAGAGATGTCGTTTCCCTTGTGGCCTGTGTGCAGGTGG
Ae263Ps1_16S       TTTGACATGCACAGGATCGCGGCAGAGATGTCGTTTCCCTTGTGGCCTGTGTGCAGGTGG
Ae356Ps1_16S       TTTGACATGCACAGGATCGCGGCAGAGATGTCGTTTCCCTTGTGGCCTGTGTGCAGGTGG
                   ************  **   *   ******   * ***********   ************

Ae706Ps2_16S       TGCATGGCTGTCGTCAGCTCGTGTCGTGAGATGTTGGGTTAAGTCCCGCAACGAGCGCAA
Ae717Ps2_16S       TGCATGGCTGTCGTCAGCTCGTGTCGTGAGATGTTGGGTTAAGTCCCGCAACGAGCGCAA
Ae505Ps2_16S       TGCATGGCTGTCGTCAGCTCGTGTCGTGAGATGTTGGGTTAAGTCCCGCAACGAGCGCAA
Ae406Ps2_16S       TGCATGGCTGTCGTCAGCTCGTGTCGTGAGATGTTGGGTTAAGTCCCGCAACGAGCGCAA
Ae331Ps2_16S       TGCATGGCTGTCGTCAGCTCGTGTCGTGAGATGTTGGGTTAAGTCCCGCAACGAGCGCAA
Ae150APs1_16S      TGCATGGCTGTCGTCAGCTCGTGTCGTGAGATGTTGGGTTAAGTCCCGCAACGAGCGCAA
Ae707Ps1_16S       TGCATGGCTGTCGTCAGCTCGTGTCGTGAGATGTTGGGTTAAGTCCCGCAACGAGCGCAA
Ae168Ps1_16S       TGCATGGCTGTCGTCAGCTCGTGTCGTGAGATGTTGGGTTAAGTCCCGCAACGAGCGCAA
Ae263Ps1_16S       TGCATGGCTGTCGTCAGCTCGTGTCGTGAGATGTTGGGTTAAGTCCCGCAACGAGCGCAA
Ae356Ps1_16S       TGCATGGCTGTCGTCAGCTCGTGTCGTGAGATGTTGGGTTAAGTCCCGCAACGAGCGCAA
                   ************************************************************

Ae706Ps2_16S       CCCTTGTTCCATGTTGCCAGCACGTTATGGTGGGGACTCATGGGAGACTGCCGGGGTCAA
Ae717Ps2_16S       CCCTTGTTCCATGTTGCCAGCACGTTATGGTGGGGACTCATGGGAGACTGCCGGGGTCAA
Ae505Ps2_16S       CCCTTGTTCCATGTTGCCAGCACGTTATGGTGGGGACTCATGGGAGACTGCCGGGGTCAA
Ae406Ps2_16S       CCCTTGTTCCATGTTGCCAGCACGTTATGGTGGGGACTCATGGGAGACTGCCGGGGTCAA
Ae331Ps2_16S       CCCTTGTTCCATGTTGCCAGCACGTTATGGTGGGGACTCATGGGAGACTGCCGGGGTCAA
Ae150APs1_16S      CCCTTATTCCATGTTGCCAGCACGTAGTGGTGGGGACTCATGGGAGACTGCCGGGGTCAA
Ae707Ps1_16S       CCCTTATTCCATGTTGCCAGCACGTAGTGGTGGGGACTCATGGGAGACTGCCGGGGTCAA
Ae168Ps1_16S       CCCTTATTCCATGTTGCCAGCACGTAGTGGTGGGGACTCATGGGAGACTGCCGGGGTCAA
Ae263Ps1_16S       CCCTTATTCCATGTTGCCAGCACGTAGTGGTGGGGACTCATGGGAGACTGCCGGGGTCAA
Ae356Ps1_16S       CCCTTATTCCATGTTGCCAGCACGTAGTGGTGGGGACTCATGGGAGACTGCCGGGGTCAA
                   ***** *******************  *********************************

Ae706Ps2_16S       CTCGGAGGAAGGTGGGGATGACGTCAAGTCATCATGCCCCTTATGTCCAGGGCTTCACAC
Ae717Ps2_16S       CTCGGAGGAAGGTGGGGATGACGTCAAGTCATCATGCCCCTTATGTCCAGGGCTTCACAC
Ae505Ps2_16S       CTCGGAGGAAGGTGGGGATGACGTCAAGTCATCATGCCCCTTATGTCCAGGGCTTCACAC
Ae406Ps2_16S       CTCGGAGGAAGGTGGGGATGACGTCAAGTCATCATGCCCCTTATGTCCAGGGCTTCACAC
Ae331Ps2_16S       CTCGGAGGAAGGTGGGGATGACGTCAAGTCATCATGCCCCTTATGTCCAGGGCTTCACAC
Ae150APs1_16S      CTCGGAGGAAGGTGGGGATGACGTCAAGTCATCATGCCCCTTATGTCCAGGGCTTCACAC
Ae707Ps1_16S       CTCGGAGGAAGGTGGGGATGACGTCAAGTCATCATGCCCCTTATGTCCAGGGCTTCACAC
Ae168Ps1_16S       CTCGGAGGAAGGTGGGGATGACGTCAAGTCATCATGCCCCTTATGTCCAGGGCTTCACAC
Ae263Ps1_16S       CTCGGAGGAAGGTGGGGATGACGTCAAGTCATCATGCCCCTTATGTCCAGGGCTTCACAC
Ae356Ps1_16S       CTCGGAGGAAGGTGGGGATGACGTCAAGTCATCATGCCCCTTATGTCCAGGGCTTCACAC
                   ************************************************************
```

```
Ae706Ps2_16S        ATGCTACAATGGCTCATACAGAGGGCTGCGAGACCGTGAGGTGGAGCGAATCCCTTAAAG
Ae717Ps2_16S        ATGCTACAATGGCTCATACAGAGGGCTGCGAGACCGTGAGGTGGAGCGAATCCCTTAAAG
Ae505Ps2_16S        ATGCTACAATGGCTCATACAGAGGGCTGCGAGACCGTGAGGTGGAGCGAATCCCTTAAAG
Ae406Ps2_16S        ATGCTACAATGGCTCATACAGAGGGCTGCGAGACCGTGAGGTGGAGCGAATCCCTTAAAG
Ae331Ps2_16S        ATGCTACAATGGCTCATACAGAGGGCTGCGAGACCGTGAGGTGGAGCGAATCCCTTAAAG
Ae150APs1_16S       ATGCTACAATGGCTCATACAGAGGGCTGCGAGACCGTGAGGTGGAGCGAATCCCTTAAAG
Ae707Ps1_16S        ATGCTACAATGGCTCATACAGAGGGCTGCGAGACTGTGAGGTGGAGCGAATCCCTTAAAG
Ae168Ps1_16S        ATGCTACAATGGCTCATACAGAGGGCTGCGAGACCGTGAGGTGGAGCGAATCCCTTAAAG
Ae263Ps1_16S        ATGCTACAATGGCTCATACAGAGGGCTGCGAGACCGTGAGGTGGAGCGAATCCCTTAAAG
Ae356Ps1_16S        ATGCTACAATGGCTCATACAGAGGGCTGCGAGACCGTGAGGTGGAGCGAATCCCTTAAAG
                    ********************************** *************************

Ae706Ps2_16S        TGAGTCTCAGTTCGGATCGGGGTCTGCAACTCGACCCCGTGAAGTTGGAGTCGCTAGTAA
Ae717Ps2_16S        TGAGTCTCAGTTCGGATCGGGGTCTGCAACTCGACCCCGTGAAGTTGGAGTCGCTAGTAA
Ae505Ps2_16S        TGAGTCTCAGTTCGGATCGGGGTCTGCAACTCGACCCCGTGAAGTTGGAGTCGCTAGTAA
Ae406Ps2_16S        TGAGTCTCAGTTCGGATCGGGGTCTGCAACTCGACCCCGTGAAGTTGGAGTCGCTAGTAA
Ae331Ps2_16S        TGAGTCTCAGTTCGGATCGGGGTCTGCAACTCGACCCCGTGAAGTTGGAGTCGCTAGTAA
Ae150APs1_16S       TGAGTCTCAGTTCGGATCGGGGTCTGCAACTCGACCCCGTGAAGTTGGAGTCGCTAGTAA
Ae707Ps1_16S        TGAGTCTCAGTTCGGATCGGGGTCTGCAACTCGACCCCGTGAAGTTGGAGTCGCTAGTAA
Ae168Ps1_16S        TGAGTCTCAGTTCGGATCGGGGTCTGCAACTCGACCCCGTGAAGTTGGAGTCGCTAGTAA
Ae263Ps1_16S        TGAGTCTCAGTTCGGATCGGGGTCTGCAACTCGACCCCGTGAAGTTGGAGTCGCTAGTAA
Ae356Ps1_16S        TGAGTCTCAGTTCGGATCGGGGTCTGCAACTCGACCCCGTGAAGTTGGAGTCGCTAGTAA
                    ************************************************************

Ae706Ps2_16S        TCGCAGATCAGCAACGCTGCGGTGAATACGTTCCCGGGCCTTGTACACACCGCCCGTCAC
Ae717Ps2_16S        TCGCAGATCAGCAACGCTGCGGTGAATACGTTCCCGGGCCTTGTACACACCGCCCGTCAC
Ae505Ps2_16S        TCGCAGATCAGCAACGCTGCGGTGAATACGTTCCCGGGCCTTGTACACACCGCCCGTCAC
Ae406Ps2_16S        TCGCAGATCAGCAACGCTGCGGTGAATACGTTCCCGGGCCTTGTACACACCGCCCGTCAC
Ae331Ps2_16S        TCGCAGATCAGCAACGCTGCGGTGAATACGTTCCCGGGCCTTGTACACACCGCCCGTCAC
Ae150APs1_16S       TCGCAGATCAGCAACGCTGCGGTGAATACGTTCCCGGGCCTTGTACACACCGCCCGTCAC
Ae707Ps1_16S        TCGCAGATCAGCAACGCTGCGGTGAATACGTTCCCGGGCCTTGTACACACCGCCCGTCAC
Ae168Ps1_16S        TCGCAGATCAGCAACGCTGCGGTGAATACGTTCCCGGGCCTTGTACACACCGCCCGTCAC
Ae263Ps1_16S        TCGCAGATCAGCAACGCTGCGGTGAATACGTTCCCGGGCCTTGTACACACCGCCCGTCAC
Ae356Ps1_16S        TCGCAGATCAGCAACGCTGCGGTGAATACGTTCCCGGGCCTTGTACACACCGCCCGTCAC
                    ************************************************************

Ae706Ps2_16S        GTCACGAAAGTTGGTAACACCCGAAGCCGGCGGCCCAACCCTTGTGGAGGGAGCTGTCGA
Ae717Ps2_16S        GTCACGAAAGTTGGTAACACCCGAAGCTGGCGGCCCAACCCTTGTGGAGGGAGCTGTCGA
Ae505Ps2_16S        GTCACGAAAGTTGGTAACACCCGAAGCCGGCGGCCCAACCCTTGTGGAGGGAGCTGTCGA
Ae406Ps2_16S        GTCACGAAAGTTGGTAACACCCGAAGCCGGCGGCCCAACCCTTGTGGAGGGAGCTGTCGA
Ae331Ps2_16S        GTCACGAAAGTTGGTAACACCCGAAGCCGGCGGCCCAACCCTTGTGGAGGGAGCTGTCGA
Ae150APs1_16S       GTCACGAAAGTTGGTAACACCCGAAGCCGGCGGCCCAACCCTTGTGGAGGGAGTCGTCGA
Ae707Ps1_16S        GTCACGAAAGTTGGTAACACCCGAAGCCGGCGGCCCAACCCTTGTGGAGGGAGTCGTCGA
Ae168Ps1_16S        GTCACGAAAGTTGGTAACACCCGAAGCCGGCGGCCCAACCCTTGTGGAGGGAGTCGTCGA
Ae263Ps1_16S        GTCACGAAAGTTGGTAACACCCGAAGCCGGCGGCCCAACCCTTGTGGAGGGAGTCGTCGA
Ae356Ps1_16S        GTCACGAAAGTTGGTAACACCCGAAGCCGGCGGCCCAACCCTTGTGGAGGGAGTCGTCGA
                    ************************** **************************  *****

Ae706Ps2_16S        AGGTGGGACTGGCGATTGGGACGAAGTCGTAACAAGGTAGCCGTACCGGAAGGTGCGGCT
Ae717Ps2_16S        AGGTGGGACTGGCGATTGGGACGAAGTCGTAACAAGGTAGCCGTACCGGAAGGTGCGGCT
Ae505Ps2_16S        AGGTGGGACTGGCGATTGGGACGAAGTCGTAACAAGGTAGCCGTACCGGAAGGTGCGGCT
Ae406Ps2_16S        AGGTGGGACTGGCGATTGGGACGAAGTCGTAACAAGGTAGCCGTACCGGAAGGTGCGGCT
Ae331Ps2_16S        AGGTGGGACTGGCGATTGGGACGAAGTCGTAACAAGGTAGCCGTACCGGAAGGTGCGGCT
Ae150APs1_16S       AGGTGGGACTGGCGATTGGGACGAAGTCGTAACAAGGTAGCCGTACCGGAAGGTGCGGCT
Ae707Ps1_16S        AGGTGGGACTGGCGATTGGGACGAAGTCGTAACAAGGTAGCCGTACCGGAAGGTGCGGCT
Ae168Ps1_16S        AGGTGGGACTGGCGATTGGGACGAAGTCGTAACAAGGTAGCCGTACCGGAAGGTGCGGCT
Ae263Ps1_16S        AGGTGGGACTGGCGATTGGGACGAAGTCGTAACAAGGTAGCCGTACCGGAAGGTGCGGCT
Ae356Ps1_16S        AGGTGGGACTGGCGATTGGGACGAAGTCGTAACAAGGTAGCCGTACCGGAAGGTGCGGCT
                    ************************************************************

Ae706Ps2_16S        GGATCACCT
Ae717Ps2_16S        GGATCACCT
Ae505Ps2_16S        GGATCACCT
Ae406Ps2_16S        GGATCACCT
Ae331Ps2_16S        GGATCACCT
Ae150APs1_16S       GGATCACCT
Ae707Ps1_16S        GGATCACCT
Ae168Ps1_16S        GGATCACCT
Ae263Ps1_16S        GGATCACCT
Ae356Ps1_16S        GGATCACCT
                    *********
```

Supplementary Table 2. 16S rRNA gene percentage identity across *Pseudonocardia* strains.

	**Ae150A_Ps1_16S**	**Ae168_Ps1_16S**	**Ae263_Ps1_16S**	**Ae356_Ps1_16S**	**Ae707_Ps1_16S**	**Ae331_Ps2_16S**	**Ae406_Ps2_16S**	**Ae505_Ps2_16S**	**Ae706_Ps2_16S**	**Ae717_Ps2_16S**
**Ae150A_Ps1_16S**	100	100	100	100	99.93	97.74	97.74	97.74	97.74	97.68
**Ae168_Ps1_16S**	100	100	100	100	99.93	97.74	97.74	97.74	97.74	97.68
**Ae263_Ps1_16S**	100	100	100	100	99.93	97.74	97.74	97.74	97.74	97.68
**Ae356_Ps1_16S**	100	100	100	100	99.93	97.74	97.74	97.74	97.74	97.68
**Ae707_Ps1_16S**	99.93	99.93	99.93	99.93	100	97.68	97.68	97.68	97.68	97.61
**Ae331_Ps2_16S**	97.74	97.74	97.74	97.74	97.68	100	100	100	100	99.93
**Ae406_Ps2_16S**	97.74	97.74	97.74	97.74	97.68	100	100	100	100	99.93
**Ae505_Ps2_16S**	97.74	97.74	97.74	97.74	97.68	100	100	100	100	99.93
**Ae706_Ps2_16S**	97.74	97.74	97.74	97.74	97.68	100	100	100	100	99.93
**Ae717_Ps2_16S**	97.68	97.68	97.68	97.68	97.61	99.93	99.93	99.93	99.93	100

Pseudonocardia rpsL gene alignment;

```
Ae706Ps2_rpsL        ATGCCCACGATCCAGCAGCTGGTCCGCAAGGGCCGCGAGGACAAGGTCTCCAAGACCAAG
Ae717Ps2_rpsL        ATGCCCACGATCCAGCAGCTGGTCCGCAAGGGCCGCGAGGACAAGGTCTCCAAGACCAAG
Ae505Ps2_rpsL        ATGCCCACGATCCAGCAGCTGGTCCGCAAGGGCCGCGAGGACAAGGTCTCCAAGACCAAG
Ae406Ps2_rpsL        ATGCCCACGATCCAGCAGCTGGTCCGCAAGGGCCGCGAGGACAAGGTCTCCAAGACCAAG
Ae331Ps2_rpsL        ATGCCCACGATCCAGCAGCTGGTCCGCAAGGGCCGCGAGGACAAGGTCTCCAAGACCAAG
Ae150APs1_rpsL       ATGCCCACGATCCAGCAGCTGGTCCGCAAGGGCCGCGAGGACAAGGTCGCGAAGACCAAG
Ae168APs1_rpsL       ATGCCCACGATCCAGCAGCTGGTCCGCAAGGGCCGCGAGGACAAGGTCGCGAAGACCAAG
Ae263APs1_rpsL       ATGCCCACGATCCAGCAGCTGGTCCGCAAGGGCCGCGAGGACAAGGTCGCGAAGACCAAG
Ae356Ps1_rpsL        ATGCCCACGATCCAGCAGCTGGTCCGCAAGGGCCGCGAGGACAAGGTCGCGAAGACCAAG
Ae707Ps1_rpsL        ATGCCCACGATCCAGCAGCTGGTCCGCAAGGGCCGCGAGGACAAGGTCGCGAAGACCAAG
                     ************************************************ * *********

Ae706Ps2_rpsL        ACCGCCGCGCTGAAGGGAAGCCCCCAGCGCCGCGGGGTCTGCACCCGCGTGTACACCACC
Ae717Ps2_rpsL        ACCGCCGCGCTGAAGGGAAGCCCCCAGCGCCGCGGGGTCTGCACCCGCGTGTACACCACC
Ae505Ps2_rpsL        ACCGCCGCGCTGAAGGGAAGCCCCCAGCGCCGCGGGGTCTGCACCCGCGTGTACACCACC
Ae406Ps2_rpsL        ACCGCCGCGCTGAAGGGAAGCCCCCAGCGCCGCGGGGTCTGCACCCGCGTGTACACCACC
Ae331Ps2_rpsL        ACCGCCGCGCTGAAGGGAAGCCCCCAGCGCCGCGGGGTCTGCACCCGCGTGTACACCACC
Ae150APs1_rpsL       ACCGCCGCGCTCAAGGGGAGCCCCCAGCGCCGCGGGGTCTGCACCCGCGTGTACACCACC
Ae168APs1_rpsL       ACCGCCGCGCTCAAGGGGAGCCCCCAGCGCCGCGGGGTCTGCACCCGCGTGTACACCACC
Ae263APs1_rpsL       ACCGCCGCGCTCAAGGGGAGCCCCCAGCGCCGCGGGGTCTGCACCCGCGTGTACACCACC
Ae356Ps1_rpsL        ACCGCCGCGCTCAAGGGGAGCCCCCAGCGCCGCGGGGTCTGCACCCGCGTGTACACCACC
Ae707Ps1_rpsL        ACCGCCGCGCTCAAGGGGAGCCCCCAGCGCCGCGGGGTCTGCACCCGCGTGTACACCACC
                     *********** ***** ******************************************
```

```
Ae706Ps2_rpsL       ACGCCCAAGAAGCCGAACTCGGCCCTGCGCAAGGTCGCCCGTGTGCGCCTGTCCAGCGGC
Ae717Ps2_rpsL       ACGCCCAAGAAGCCGAACTCGGCCCTGCGCAAGGTCGCCCGTGTGCGCCTGTCCAGCGGC
Ae505Ps2_rpsL       ACGCCCAAGAAGCCGAACTCGGCCCTGCGCAAGGTCGCCCGTGTGCGCCTGTCCAGCGGC
Ae406Ps2_rpsL       ACGCCCAAGAAGCCGAACTCGGCCCTGCGCAAGGTCGCCCGTGTGCGCCTGTCCAGCGGC
Ae331Ps2_rpsL       ACGCCCAAGAAGCCGAACTCGGCCCTGCGCAAGGTCGCCCGTGTGCGCCTGTCCAGCGGC
Ae150APs1_rpsL      ACCCCGAAGAAGCCGAACTCCGCGCTTCGCAAGGTCGCCCGTGTGCGGCTCTCGAGCGGC
Ae168APs1_rpsL      ACCCCGAAGAAGCCGAACTCCGCGCTTCGCAAGGTCGCCCGTGTGCGGCTCTCGAGCGGC
Ae263APs1_rpsL      ACCCCGAAGAAGCCGAACTCCGCGCTTCGCAAGGTCGCCCGTGTGCGGCTCTCGAGCGGC
Ae356Ps1_rpsL       ACCCCGAAGAAGCCGAACTCCGCGCTTCGCAAGGTCGCCCGTGTGCGGCTCTCGAGCGGC
Ae707Ps1_rpsL       ACCCCGAAGAAGCCGAACTCCGCGCTTCGCAAGGTCGCCCGTGTGCGGCTCTCGAGCGGC
                    ** ** ************** ** ** ******************** ** ** ******

Ae706Ps2_rpsL       ATCGAGGTCACCGCGTACATCCCCGGTGAGGGCCACAACCTGCAGGAGCACAGCATCGTG
Ae717Ps2_rpsL       ATCGAGGTCACCGCGTACATCCCCGGTGAGGGCCACAACCTGCAGGAGCACAGCATCGTG
Ae505Ps2_rpsL       ATCGAGGTCACCGCGTACATCCCCGGTGAGGGCCACAACCTGCAGGAGCACAGCATCGTG
Ae406Ps2_rpsL       ATCGAGGTCACCGCGTACATCCCCGGTGAGGGCCACAACCTGCAGGAGCACAGCATCGTG
Ae331Ps2_rpsL       ATCGAGGTCACCGCGTACATCCCCGGTGAGGGCCACAACCTGCAGGAGCACAGCATCGTG
Ae150APs1_rpsL      ATCGAGGTCACCGCCTACATCCCCGGTGAGGGTCACAACCTGCAGGAGCACTCCATCGTG
Ae168APs1_rpsL      ATCGAGGTCACCGCCTACATCCCCGGTGAGGGTCACAACCTGCAGGAGCACTCCATCGTG
Ae263APs1_rpsL      ATCGAGGTCACCGCCTACATCCCCGGTGAGGGTCACAACCTGCAGGAGCACTCCATCGTG
Ae356Ps1_rpsL       ATCGAGGTCACCGCCTACATCCCCGGTGAGGGTCACAACCTGCAGGAGCACTCCATCGTG
Ae707Ps1_rpsL       ATCGAGGTCACCGCCTACATCCCCGGTGAGGGTCACAACCTGCAGGAGCACTCCATCGTG
                    ************** ***************** ******************  *******

Ae706Ps2_rpsL       CTCGTGCGTGGTGGTCGTGTGAAGGACCTCCCCGGCGTGCGCTACAAGGTCATCCGGGGG
Ae717Ps2_rpsL       CTCGTGCGTGGTGGTCGTGTGAAGGACCTCCCCGGCGTGCGCTACAAGGTCATCCGGGGG
Ae505Ps2_rpsL       CTCGTGCGTGGTGGTCGTGTGAAGGACCTCCCCGGCGTGCGCTACAAGGTCATCCGGGGG
Ae406Ps2_rpsL       CTCGTGCGTGGTGGTCGTGTGAAGGACCTCCCCGGCGTGCGCTACAAGGTCATCCGGGGG
Ae331Ps2_rpsL       CTCGTGCGTGGTGGTCGTGTGAAGGACCTCCCCGGCGTGCGCTACAAGGTCATCCGGGGG
Ae150APs1_rpsL      CTGGTGCGCGGTGGCCGAGTGAAGGACCTGCCGGGCGTGCGCTACAAGGTGATCCGCGGC
Ae168APs1_rpsL      CTGGTGCGCGGTGGCCGAGTGAAGGACCTGCCGGGCGTGCGCTACAAGGTGATCCGCGGC
Ae263APs1_rpsL      CTGGTGCGCGGTGGCCGAGTGAAGGACCTGCCGGGCGTGCGCTACAAGGTGATCCGCGGC
Ae356Ps1_rpsL       CTGGTGCGCGGTGGCCGAGTGAAGGACCTGCCGGGCGTGCGCTACAAGGTGATCCGCGGC
Ae707Ps1_rpsL       CTGGTGCGCGGTGGCCGAGTGAAGGACCTGCCGGGCGTGCGCTACAAGGTGATCCGCGGC
                    ** ***** ***** ** *********** ** ***************** ***** ** 

Ae706Ps2_rpsL       TCGCTGGACACCCAGGGCGTGAAGAACCGCAAGCAGTCCCGCAGCCGCTACGGCGCGAAG
Ae717Ps2_rpsL       TCGCTGGACACCCAGGGCGTGAAGAACCGCAAGCAGTCCCGCAGCCGCTACGGCGCGAAG
Ae505Ps2_rpsL       TCGCTGGACACCCAGGGCGTGAAGAACCGCAAGCAGTCCCGCAGCCGCTACGGCGCGAAG
Ae406Ps2_rpsL       TCGCTGGACACCCAGGGCGTGAAGAACCGCAAGCAGTCCCGCAGCCGCTACGGCGCGAAG
Ae331Ps2_rpsL       TCGCTGGACACCCAGGGCGTGAAGAACCGCAAGCAGTCCCGCAGCCGCTACGGCGCGAAG
Ae150APs1_rpsL      TCGCTGGACACCCAGGGCGTCAAGAACCGCAAGCAGTCCCGCAGCCGGTACGGCGCGAAG
Ae168APs1_rpsL      TCGCTGGACACCCAGGGCGTCAAGAACCGCAAGCAGTCCCGCAGCCGGTACGGCGCGAAG
Ae263APs1_rpsL      TCGCTGGACACCCAGGGCGTCAAGAACCGCAAGCAGTCCCGCAGCCGGTACGGCGCGAAG
Ae356Ps1_rpsL       TCGCTGGACACCCAGGGCGTCAAGAACCGCAAGCAGTCCCGCAGCCGGTACGGCGCGAAG
Ae707Ps1_rpsL       TCGCTGGACACCCAGGGCGTCAAGAACCGCAAGCAGTCCCGCAGCCGGTACGGCGCGAAG
                    ******************** ************************** ************

Ae706Ps2_rpsL       AAGGAGAAGAGCTGA
Ae717Ps2_rpsL       AAGGAGAAGAGCTGA
Ae505Ps2_rpsL       AAGGAGAAGAGCTGA
Ae406Ps2_rpsL       AAGGAGAAGAGCTGA
Ae331Ps2_rpsL       AAGGAGAAGAGCTGA
Ae150APs1_rpsL      AAGGAGAAGAGCTGA
Ae168APs1_rpsL      AAGGAGAAGAGCTGA
Ae263APs1_rpsL      AAGGAGAAGAGCTGA
Ae356Ps1_rpsL       AAGGAGAAGAGCTGA
Ae707Ps1_rpsL       AAGGAGAAGAGCTGA
                    ***************
```

Supplementary Table 3. *rpsL* gene percentage identity across *Pseudonocardia* strains.

	**Ae150A_Ps1_*rpsL***	**Ae168A_Ps1_*rpsL***	**Ae263A_Ps1_*rpsL***	**Ae356_Ps1_*rpsL***	**Ae707_Ps1_*rpsL***	**Ae331_Ps2_*rpsL***	**Ae406_Ps2_*rpsL***	**Ae505_Ps2_*rpsL***	**Ae706_Ps2_*rpsL***	**Ae717_Ps2_*rpsL***
**Ae150A_Ps1_*rpsL***	100	100	100	100	100	92.8	92.8	92.8	92.8	92.8
**Ae168A_Ps1_*rpsL***	100	100	100	100	100	92.8	92.8	92.8	92.8	92.8
**Ae263A_Ps1_*rpsL***	100	100	100	100	100	92.8	92.8	92.8	92.8	92.8
**Ae356Ps1_*rpsL***	100	100	100	100	100	92.8	92.8	92.8	92.8	92.8
**Ae707_Ps1_*rpsL***	100	100	100	100	100	92.8	92.8	92.8	92.8	92.8
**Ae331_Ps2_*rpsL***	92.8	92.8	92.8	92.8	92.8	100	100	100	100	100
**Ae406_Ps2_*rpsL***	92.8	92.8	92.8	92.8	92.8	100	100	100	100	100
**Ae505_Ps2_*rpsL***	92.8	92.8	92.8	92.8	92.8	100	100	100	100	100
**Ae706_Ps2_*rpsL***	92.8	92.8	92.8	92.8	92.8	100	100	100	100	100
**Ae717_Ps2_*rpsL***	92.8	92.8	92.8	92.8	92.8	100	100	100	100	100

Pseudonocardia rpoB gene alignment;

```
Ae331Ps2_rpoB        TTGGCTGTCTCCCGCGCCGCCGCGACCACCTCGACCCTCTCCGCTTCGGCGAACCCGAAC
Ae505Ps2_rpoB        TTGGCTGTCTCCCGCGCCGCCGCGACCACCTCGACCCTCTCCGCTTCGGCGAACCCGAAC
Ae406Ps2_rpoB        TTGGCTGTCTCCCGCGCCGCCGCGACCACCTCGACCCTCTCCGCTTCGGCGAACCCGAAC
Ae717Ps2_rpoB        TTGGCTGTCTCCCGCGCCGCCGCGACCACCTCGACCCTCTCCGCTTCGGCGAACCCGAAC
Ae706Ps2_rpoB        TTGGCTGTCTCCCGCGCCGCCGCGACCACCTCGACCCTCTCCGCTTCGGCGAACCCGAAC
Ae150APs1_rpoB       TTGGCTGTCTCCCGCGCCGCCGCGACCACCTCGACCCTCTCCGCTTCGGCGAACCCGAAC
Ae168Ps1_rpoB        TTGGCTGTCTCCCGCGCCGCCGCGACCACCTCGACCCTCTCCGCTTCGGCGAACCCGAAC
Ae263Ps1_rpoB        TTGGCTGTCTCCCGCGCCGCCGCGACCACCTCGACCCTCTCCGCTTCGGCGAACCCGAAC
Ae356Ps1_rpoB        TTGGCTGTCTCCCGCGCCGCCGCGACCACCTCGACCCTCTCCGCTTCGGCGAACCCGAAC
Ae707Ps1_rpoB        TTGGCTGTCTCCCGCGCCGCCGCGACCACCTCGACCCTCTCCGCTTCGGCGAACCCGAAC
                     ************************************************************

Ae331Ps2_rpoB        TACCCCGGCGCACCCACCCGGGTCACCTTCGCGAAGATCGCCCAGCCGTTGGAGGTCCCC
Ae505Ps2_rpoB        TGCCCCGGCGCACCCACCCGGGTCACCTTCGCGAAGATCGCCCAGCCGTTGGAGGTCCCC
Ae406Ps2_rpoB        TACCCCGGCGCACCCACCCGGGTCACCTTCGCGAAGATCGCCCAGCCGTTGGAGGTCCCC
Ae717Ps2_rpoB        TACCCCGGCGCACCCACCCGGGTCACCTTCGCGAAGATCGCCCAGCCGTTGGAGGTCCCC
Ae706Ps2_rpoB        TACCCCGGCGCACCCACCCGGGTCACCTTCGCGAAGATCGCCCAGCCGTTGGAGGTCCCC
Ae150APs1_rpoB       TACCCCGGTGCACCCACTCGGGTCACCTTCGCGAAGATCTCCGAGCCGCTGGAGGTCCCG
Ae168Ps1_rpoB        TACCCCGGTGCACCCACTCGGGTCACCTTCGCGAAGATCTCCGAGCCGCTGGAGGTCCCG
Ae263Ps1_rpoB        TACCCCGGTGCACCCACTCGGGTCACCTTCGCGAAGATCTCCGAGCCGCTGGAGGTCCCG
Ae356Ps1_rpoB        TACCCCGGTGCACCCACTCGGGTCACCTTCGCGAAGATCTCCGAGCCGCTGGAGGTCCCG
Ae707Ps1_rpoB        TACCCCGGTGCACCCACTCGGGTCACCTTCGCGAAGATCTCCGAGCCGCTGGAGGTCCCG
                     * ****** ******** ********************* ** ***** ********** 
```

```
Ae331Ps2_rpoB       GACCTTCTCGACCTGCAGATCCAGTCCTACGAGTGGCTGGTCGGCAGCGAGGCCTGGTTC
Ae505Ps2_rpoB       GACCTTCTCGACCTGCAGATCCAGTCCTACGAGTGGCTGGTCGGCAGCGAGGCCTGGTTC
Ae406Ps2_rpoB       GACCTTCTCGACCTGCAGATCCAGTCCTACGAGTGGCTGGTCGGCAGCGAGGCCTGGTTC
Ae717Ps2_rpoB       GACCTTCTCGACCTGCAGATCCAGTCCTACGAGTGGCTGGTCGGCAGCGAGGCCTGGTTC
Ae706Ps2_rpoB       GACCTTCTCGACCTGCAGATCCAGTCCTACGAGTGGCTGGTCGGCAGCGAGGCCTGGTTC
Ae150APs1_rpoB      GACCTCCTCGACCTGCAGATCCAGTCCTACGAGTGGCTCGTCGGCAGCGAGGCCTGGTTC
Ae168Ps1_rpoB       GACCTCCTCGACCTGCAGATCCAGTCCTACGAGTGGCTCGTCGGCAGCGAGGCCTGGTTC
Ae263Ps1_rpoB       GACCTCCTCGACCTGCAGATCCAGTCCTACGAGTGGCTCGTCGGCAGCGAGGCCTGGTTC
Ae356Ps1_rpoB       GACCTCCTCGACCTGCAGATCCAGTCCTACGAGTGGCTCGTCGGCAGCGAGGCCTGGTTC
Ae707Ps1_rpoB       GACCTCCTCGACCTGCAGATCCAGTCCTACGAGTGGCTCGTCGGCAGCGAGGCCTGGTTC
                    ***** ******************************** *********************

Ae331Ps2_rpoB       CAGCGCCGGATCGAGGCCGGTGACGAGCTTCCGGTGAGCGGTCTGGAGGAGATCCTCACC
Ae505Ps2_rpoB       CAGCGCCGGATCGAGGCCGGTGACGAGCTTCCGGTGAGCGGTCTGGAGGAGATCCTCACC
Ae406Ps2_rpoB       CAGCGCCGGATCGAGGCCGGTGACGAGCTTCCGGTGAGCGGTCTGGAGGAGATCCTCACC
Ae717Ps2_rpoB       CAGCGCCGGATCGAGGCCGGTGACGAGCTTCCGGTGAGCGGTCTGGAGGAGATCCTCACC
Ae706Ps2_rpoB       CAGCGCCGGATCGAGGCCGGTGACGAGCTTCCGGTGAGCGGTCTGGAGGAGATCCTCACC
Ae150APs1_rpoB      CAGCGCCGGATCGAGGCCGGCGACGACCTCCCCGTGAGCGGTCTCGAGGAGATCCTCACC
Ae168Ps1_rpoB       CAGCGCCGGATCGAGGCCGGCGACGACCTCCCCGTGAGCGGTCTCGAGGAGATCCTCACC
Ae263Ps1_rpoB       CAGCGCCGGATCGAGGCCGGCGACGACCTCCCCGTGAGCGGTCTCGAGGAGATCCTCACC
Ae356Ps1_rpoB       CAGCGCCGGATCGAGGCCGGCGACGACCTCCCCGTGAGCGGTCTCGAGGAGATCCTCACC
Ae707Ps1_rpoB       CAGCGCCGGATCGAGGCCGGCGACGACCTCCCCGTGAGCGGTCTCGAGGAGATCCTCACC
                    ******************** ***** ** ** *********** ***************

Ae331Ps2_rpoB       GAGATCTCGCCGATCGAGGACTTCTCCGGGTCGATGTCGCTGTCCTTCTCCGACCCTCGC
Ae505Ps2_rpoB       GAGATCTCGCCGATCGAGGACTTCTCCGGGTCGATGTCGCTGTCCTTCTCCGACCCTCGC
Ae406Ps2_rpoB       GAGATCTCGCCGATCGAGGACTTCTCCGGGTCGATGTCGCTGTCCTTCTCCGACCCTCGC
Ae717Ps2_rpoB       GAGATCTCGCCGATCGAGGACTTCTCCGGGTCGATGTCGCTGTCCTTCTCCGACCCTCGC
Ae706Ps2_rpoB       GAGATCTCGCCGATCGAGGACTTCTCCGGGTCGATGTCGCTGTCCTTCTCCGACCCTCGC
Ae150APs1_rpoB      GAGATCTCCCCGATCGAGGACTTCTCCGGGTCGATGTCGCTGTCCTTCTCCGACCCCCGC
Ae168Ps1_rpoB       GAGATCTCCCCGATCGAGGACTTCTCCGGGTCGATGTCGCTGTCCTTCTCCGACCCCCGC
Ae263Ps1_rpoB       GAGATCTCCCCGATCGAGGACTTCTCCGGGTCGATGTCGCTGTCCTTCTCCGACCCCCGC
Ae356Ps1_rpoB       GAGATCTCCCCGATCGAGGACTTCTCCGGGTCGATGTCGCTGTCCTTCTCCGACCCCCGC
Ae707Ps1_rpoB       GAGATCTCCCCGATCGAGGACTTCTCCGGGTCGATGTCGCTGTCCTTCTCCGACCCCCGC
                    ******** *********************************************** ***

Ae331Ps2_rpoB       TTCGACGAGGTCAAGGCCTCCGTCGAGGAGTGCCGGGACAAGGACATGACCTACGGTGCC
Ae505Ps2_rpoB       TTCGACGAGGTCAAGGCCTCCGTCGAGGAGTGCCGGGACAAGGACATGACCTACGGTGCC
Ae406Ps2_rpoB       TTCGACGAGGTCAAGGCCTCCGTCGAGGAGTGCCGGGACAAGGACATGACCTACGGTGCC
Ae717Ps2_rpoB       TTCGACGAGGTCAAGGCCTCCGTCGAGGAGTGCCGGGACAAGGACATGACCTACGGTGCC
Ae706Ps2_rpoB       TTCGACGAGGTCAAGGCCTCCGTCGAGGAGTGCCGGGACAAGGACATGACCTACGGTGCC
Ae150APs1_rpoB      TTCGACGAGGTCAAGGCCTCCGTCGAGGAGTGCCGGGACAAAGACATGACCTACGGGGCC
Ae168Ps1_rpoB       TTCGACGAGGTCAAGGCCTCCGTCGAGGAGTGCCGGGACAAAGACATGACCTACGGGGCC
Ae263Ps1_rpoB       TTCGACGAGGTCAAGGCCTCCGTCGAGGAGTGCCGGGACAAAGACATGACCTACGGGGCC
Ae356Ps1_rpoB       TTCGACGAGGTCAAGGCCTCCGTCGAGGAGTGCCGGGACAAAGACATGACCTACGGGGCC
Ae707Ps1_rpoB       TTCGACGAGGTCAAGGCCTCCGTCGAGGAGTGCCGGGACAAGGACATGACCTACGGGGCC
                    ***************************************** ************** ***

Ae331Ps2_rpoB       CCGCTGTTCGTGACGGCGGAGTTCACCAACAACACCACCGGTGAGATCAAGTCCCAGACG
Ae505Ps2_rpoB       CCGCTGTTCGTGACGGCGGAGTTCACCAACAACACCACCGGTGAGATCAAGTCCCAGACG
Ae406Ps2_rpoB       CCGCTGTTCGTGACGGCGGAGTTCACCAACAACACCACCGGTGAGATCAAGTCCCAGACG
Ae717Ps2_rpoB       CCGCTGTTCGTGACGGCGGAGTTCACCAACAACACCACCGGTGAGATCAAGTCCCAGACG
Ae706Ps2_rpoB       CCGCTGTTCGTGACGGCGGAGTTCACCAACAACACCACCGGTGAGATCAAGTCCCAGACG
Ae150APs1_rpoB      CCGCTGTTCGTCACGGCGGAGTTCACGAACAACACCACCGGTGAGATCAAGTCCCAGACC
Ae168Ps1_rpoB       CCGCTGTTCGTCACGGCGGAGTTCACGAACAACACCACCGGTGAGATCAAGTCCCAGACC
Ae263Ps1_rpoB       CCGCTGTTCGTCACGGCGGAGTTCACGAACAACACCACCGGTGAGATCAAGTCCCAGACC
Ae356Ps1_rpoB       CCGCTGTTCGTCACGGCGGAGTTCACGAACAACACCACCGGTGAGATCAAGTCCCAGACC
Ae707Ps1_rpoB       CCGCTGTTCGTCACGGCGGAGTTCACGAACAACACCACCGGTGAGATCAAGTCCCAGACC
                    *********** ************** ******************************** 

Ae331Ps2_rpoB       GTCTTCATGGGCGAGTTCCCCGTGATGACCGACAAGGGCACGTTCATCATCAACGGCACC
Ae505Ps2_rpoB       GTCTTCATGGGCGAGTTCCCCGTGATGACCGACAAGGGCACGTTCATCATCAACGGCACC
Ae406Ps2_rpoB       GTCTTCATGGGCGAGTTCCCCGTGATGACCGACAAGGGCACGTTCATCATCAACGGCACC
Ae717Ps2_rpoB       GTCTTCATGGGCGAGTTCCCCGTGATGACCGACAAGGGCACGTTCATCATCAACGGCACC
Ae706Ps2_rpoB       GTCTTCATGGGCGAGTTCCCCGTGATGACCGACAAGGGCACGTTCATCATCAACGGCACC
Ae150APs1_rpoB      GTGTTCATGGGTGAGTTCCCCGTGATGACGGACAAGGGCACCTTCATCATCAACGGCACC
Ae168Ps1_rpoB       GTGTTCATGGGTGAGTTCCCCGTGATGACGGACAAGGGCACCTTCATCATCAACGGCACC
Ae263Ps1_rpoB       GTGTTCATGGGTGAGTTCCCCGTGATGACGGACAAGGGCACCTTCATCATCAACGGCACC
Ae356Ps1_rpoB       GTGTTCATGGGTGAGTTCCCCGTGATGACGGACAAGGGCACCTTCATCATCAACGGCACC
Ae707Ps1_rpoB       GTGTTCATGGGTGAGTTCCCCGTGATGACGGACAAGGGCACCTTCATCATCAACGGCACC
                    ** ******** ***************** *********** ******************

Ae331Ps2_rpoB       GAGCGCGTCGTCGTGTCGCAGCTCGTCCGTTCGCCGGGTGTCTACTTCGACCACGCGATC
Ae505Ps2_rpoB       GAGCGCGTCGTCGTGTCGCAGCTCGTCCGTTCGCCGGGTGTCTACTTCGACCACGCGATC
Ae406Ps2_rpoB       GAGCGCGTCGTCGTGTCGCAGCTCGTCCGTTCGCCGGGTGTCTACTTCGACCACGCGATC
Ae717Ps2_rpoB       GAGCGCGTCGTCGTGTCGCAGCTCGTCCGTTCGCCGGGTGTCTACTTCGACCACGCGATC
Ae706Ps2_rpoB       GAGCGCGTCGTCGTGTCGCAGCTCGTCCGTTCGCCGGGTGTCTACTTCGACCACGCGATC
```

```
Ae150APs1_rpoB      GAGCGCGTCGTCGTGTCGCAGCTCGTCCGGTCGCCGGGTGTGTACTTCGACCACTCGATC
Ae168Ps1_rpoB       GAGCGCGTCGTCGTGTCGCAGCTCGTCCGGTCGCCGGGTGTGTACTTCGACCACTCGATC
Ae263Ps1_rpoB       GAGCGCGTCGTCGTGTCGCAGCTCGTCCGGTCGCCGGGTGTGTACTTCGACCACTCGATC
Ae356Ps1_rpoB       GAGCGCGTCGTCGTGTCGCAGCTCGTCCGGTCGCCGGGTGTGTACTTCGACCACTCGATC
Ae707Ps1_rpoB       GAGCGCGTCGTCGTGTCGCAGCTCGTCCGGTCGCCGGGTGTGTACTTCGACCACTCGATC
                    ***************************** *********** ************ *****

Ae331Ps2_rpoB       GACAAGACGACCGAGAAGGACGTCTACTCGGTCAAGATCATCCCGAGCCGGGGCGCGTGG
Ae505Ps2_rpoB       GACAAGACGACCGAGAAGGACGTCTACTCGGTCAAGATCATCCCGAGCCGGGGCGCGTGG
Ae406Ps2_rpoB       GACAAGACGACCGAGAAGGACGTCTACTCGGTCAAGATCATCCCGAGCCGGGGCGCGTGG
Ae717Ps2_rpoB       GACAAGACGACCGAGAAGGACGTCTACTCGGTCAAGATCATCCCGAGCCGGGGCGCGTGG
Ae706Ps2_rpoB       GACAAGACGACCGAGAAGGACGTCTACTCGGTCAAGATCATCCCGAGCCGGGGCGCGTGG
Ae150APs1_rpoB      GACAAGACGACCGAGAAGGACGTCTACTCGGTCAAGATCATCCCGAGCCGGGGTGCGTGG
Ae168Ps1_rpoB       GACAAGACGACCGAGAAGGACGTCTACTCGGTCAAGATCATCCCGAGCCGGGGTGCGTGG
Ae263Ps1_rpoB       GACAAGACGACCGAGAAGGACGTCTACTCGGTCAAGATCATCCCGAGCCGGGGTGCGTGG
Ae356Ps1_rpoB       GACAAGACGACCGAGAAGGACGTCTACTCGGTCAAGATCATCCCGAGCCGGGGTGCGTGG
Ae707Ps1_rpoB       GACAAGACGACCGAGAAGGACGTCTACTCGGTCAAGATCATCCCGAGCCGGGGTGCATGG
                    ***************************************************** ** ***

Ae331Ps2_rpoB       CTGGAGTTCGACGTCGACAAGCGCGACACCGTCGGTGTCCGCATCGACCGCAAGCGCCGC
Ae505Ps2_rpoB       CTGGAGTTCGACGTCGACAAGCGCGACACCGTCGGTGTCCGCATCGACCGCAAGCGCCGC
Ae406Ps2_rpoB       CTGGAGTTCGACGTCGACAAGCGCGACACCGTCGGTGTCCGCATCGACCGCAAGCGCCGC
Ae717Ps2_rpoB       CTGGAGTTCGACGTCGACAAGCGCGACACCGTCGGTGTCCGCATCGACCGCAAGCGCCGC
Ae706Ps2_rpoB       CTGGAGTTCGACGTCGACAAGCGCGACACCGTCGGTGTCCGCATCGACCGCAAGCGCCGC
Ae150APs1_rpoB      CTGGAGTTCGACGTCGACAAGCGCGACACCGTCGGTGTCCGCATCGACCGCAAGCGCCGC
Ae168Ps1_rpoB       CTGGAGTTCGACGTCGACAAGCGCGACACCGTCGGTGTCCGCATCGACCGCAAGCGCCGC
Ae263Ps1_rpoB       CTGGAGTTCGACGTCGACAAGCGCGACACCGTCGGTGTCCGCATCGACCGCAAGCGCCGC
Ae356Ps1_rpoB       CTGGAGTTCGACGTCGACAAGCGCGACACCGTCGGTGTCCGCATCGACCGCAAGCGCCGC
Ae707Ps1_rpoB       CTGGAGTTCGACGTCGACAAGCGCGACACCGTCGGTGTCCGCATCGACCGCAAGCGCCGC
                    ************************************************************

Ae331Ps2_rpoB       CAGCCGGTCACCGTGCTGCTGAAGGCCCTGGGCTGGACCGCCGAGCAGATCGGTGAGCGT
Ae505Ps2_rpoB       CAGCCGGTCACCGTGCTGCTGAAGGCCCTGGGCTGGACCGCCGAGCAGATCGGTGAGCGT
Ae406Ps2_rpoB       CAGCCGGTCACCGTGCTGCTGAAGGCCCTGGGCTGGACCGCCGAGCAGATCGGTGAGCGT
Ae717Ps2_rpoB       CAGCCGGTCACCGTGCTGCTGAAGGCCCTGGGCTGGACCGCCGAGCAGATCGGTGAGCGT
Ae706Ps2_rpoB       CAGCCGGTCACCGTGCTGCTGAAGGCCCTGGGCTGGACCGCCGAGCAGATCGGTGAGCGT
Ae150APs1_rpoB      CAGCCGGTCACCGTGCTGCTGAAGGCGCTGGGCTGGACGGCCGAGCAGATCGGCGAGCGT
Ae168Ps1_rpoB       CAGCCGGTCACCGTGCTGCTGAAGGCGCTGGGCTGGACGGCCGAGCAGATCGGCGAGCGT
Ae263Ps1_rpoB       CAGCCGGTCACCGTGCTGCTGAAGGCGCTGGGCTGGACGGCCGAGCAGATCGGCGAGCGT
Ae356Ps1_rpoB       CAGCCGGTCACCGTGCTGCTGAAGGCGCTGGGCTGGACGGCCGAGCAGATCGGCGAGCGT
Ae707Ps1_rpoB       CAGCCGGTCACCGTGCTGCTGAAGGCGCTGGGCTGGACGGCCGAGCAGATCGGCGAGCGT
                    ************************** *********** ************** ******

Ae331Ps2_rpoB       TTCGGGTTCTCCGAGACGATCATGACGACGCTCGAGAAGGACAACACCGCGGGCCAGGAC
Ae505Ps2_rpoB       TTCGGGTTCTCCGAGACGATCATGACGACGCTCGAGAAGGACAACACCGCGGGCCAGGAC
Ae406Ps2_rpoB       TTCGGGTTCTCCGAGACGATCATGACGACGCTCGAGAAGGACAACACCGCGGGCCAGGAC
Ae717Ps2_rpoB       TTCGGGTTCTCCGAGACGATCATGACGACGCTCGAGAAGGACAACACCGCGGGCCAGGAC
Ae706Ps2_rpoB       TTCGGGTTCTCCGAGACGATCATGACGACGCTCGAGAAGGACAACACCGCGGGCCAGGAC
Ae150APs1_rpoB      TTCGGGTTCTCCGAGACGATCATGACCACGCTGGAGAAGGACAACACCGCGGGTCAGGAC
Ae168Ps1_rpoB       TTCGGGTTCTCCGAGACGATCATGACCACGCTGGAGAAGGACAACACCGCGGGTCAGGAC
Ae263Ps1_rpoB       TTCGGGTTCTCCGAGACGATCATGACCACGCTGGAGAAGGACAACACCGCGGGTCAGGAC
Ae356Ps1_rpoB       TTCGGGTTCTCCGAGACGATCATGACCACGCTGGAGAAGGACAACACCGCGGGTCAGGAC
Ae707Ps1_rpoB       TTCGGGTTCTCCGAGACGATCATGACCACGCTGGAGAAGGACAACACCGCGGGTCAGGAC
                    ************************** ***** ******************** ******

Ae331Ps2_rpoB       GAGGCGCTGCTCGACATCTACCGCAAGCTGCGCCCCGGCGAGCCGCCGACGCGCGAGTCG
Ae505Ps2_rpoB       GAGGCGCTGCTCGACATCTACCGCAAGCTGCGCCCCGGCGAGCCGCCGACGCGCGAGTCG
Ae406Ps2_rpoB       GAGGCGCTGCTCGACATCTACCGCAAGCTGCGCCCCGGCGAGCCGCCGACGCGCGAGTCG
Ae717Ps2_rpoB       GAGGCGCTGCTCGACATCTACCGCAAGCTGCGCCCCGGCGAGCCGCCGACGCGCGAGTCG
Ae706Ps2_rpoB       GAGGCGCTGCTCGACATCTACCGCAAGCTGCGCCCCGGCGAGCCGCCGACGCGCGAGTCG
Ae150APs1_rpoB      GAGGCGCTGCTCGACATCTACCGCAAGCTGCGCCCCGGTGAGCCGCCGACGCGCGAGAGC
Ae168Ps1_rpoB       GAGGCGCTGCTCGACATCTACCGCAAGCTGCGCCCCGGTGAGCCGCCGACGCGCGAGAGC
Ae263Ps1_rpoB       GAGGCGCTGCTCGACATCTACCGCAAGCTGCGCCCCGGTGAGCCGCCGACGCGCGAGAGC
Ae356Ps1_rpoB       GAGGCGCTGCTCGACATCTACCGCAAGCTGCGCCCCGGTGAGCCGCCGACGCGCGAGAGC
Ae707Ps1_rpoB       GAGGCGCTGCTCGACATCTACCGCAAGCTGCGCCCCGGTGAGCCGCCGACGCGCGAGAGC
                    ************************************** ******************

Ae331Ps2_rpoB       GCCCAGGCGCTCCTGGAGAACCTGTTCTTCAAGGACAAGCGCTACGACATGGCCAAGGTC
Ae505Ps2_rpoB       GCCCAGGCGCTCCTGGAGAACCTGTTCTTCAAGGACAAGCGCTACGACATGGCCAAGGTC
Ae406Ps2_rpoB       GCCCAGGCGCTCCTGGAGAACCTGTTCTTCAAGGACAAGCGCTACGACATGGCCAAGGTC
Ae717Ps2_rpoB       GCCCAGGCGCTCCTGGAGAACCTGTTCTTCAAGGACAAGCGCTACGACATGGCCAAGGTC
Ae706Ps2_rpoB       GCCCAGGCGCTCCTGGAGAACCTGTTCTTCAAGGACAAGCGCTACGACATGGCCAAGGTC
Ae150APs1_rpoB      GCCCAGGCCCTCCTGGAGAACCTGTTCTTCAAGGACAAGCGCTACGACATGGCCAAGGTC
Ae168Ps1_rpoB       GCCCAGGCCCTCCTGGAGAACCTGTTCTTCAAGGACAAGCGCTACGACATGGCCAAGGTC
Ae263Ps1_rpoB       GCCCAGGCCCTCCTGGAGAACCTGTTCTTCAAGGACAAGCGCTACGACATGGCCAAGGTC
Ae356Ps1_rpoB       GCCCAGGCCCTCCTGGAGAACCTGTTCTTCAAGGACAAGCGCTACGACATGGCCAAGGTC
Ae707Ps1_rpoB       GCCCAGGCCCTCCTGGAGAACCTGTTCTTCAAGGACAAGCGCTACGACATGGCCAAGGTC
```

```
                     ******** ***************************************************

Ae331Ps2_rpoB        GGTCGCTACAAGGCCAACAAGAAGCTCGGTCTCGACATCGACAACTCGGTCGGCACGCTG
Ae505Ps2_rpoB        GGTCGCTACAAGGCCAACAAGAAGCTCGGTCTCGACATCGACAACTCGGTCGGCACGCTG
Ae406Ps2_rpoB        GGTCGCTACAAGGCCAACAAGAAGCTCGGTCTCGACATCGACAACTCGGTCGGCACGCTG
Ae717Ps2_rpoB        GGTCGCTACAAGGCCAACAAGAAGCTCGGTCTCGACATCGACAACTCGGTCGGCACGCTG
Ae706Ps2_rpoB        GGTCGCTACAAGGCCAACAAGAAGCTCGGTCTCGACATCGACAACTCGGTCGGCACGCTG
Ae150APs1_rpoB       GGGCGCTACAAGGCCAACAAGAAGCTCGGTCTGGACATCGAGAACTCGGTCGGCACGCTG
Ae168Ps1_rpoB        GGGCGCTACAAGGCCAACAAGAAGCTCGGTCTGGACATCGAGAACTCGGTCGGCACGCTG
Ae263Ps1_rpoB        GGGCGCTACAAGGCCAACAAGAAGCTCGGTCTGGACATCGAGAACTCGGTCGGCACGCTG
Ae356Ps1_rpoB        GGGCGCTACAAGGCCAACAAGAAGCTCGGTCTGGACATCGAGAACTCGGTCGGCACGCTG
Ae707Ps1_rpoB        GGGCGCTACAAGGCCAACAAGAAGCTCGGTCTGGACATCGAGAACTCGGTCGGCACGCTG
                     ** ***************************** ******** ******************

Ae331Ps2_rpoB        ACCGAGGAGGATGTCGCCACCACCATCGAGTACCTCGTCCGGCTGCACGCCGGCGAGACC
Ae505Ps2_rpoB        ACCGAGGAGGATGTCGCCACCACCATCGAGTACCTCGTCCGGCTGCACGCCGGCGAGACC
Ae406Ps2_rpoB        ACCGAGGAGGATGTCGCCACCACCATCGAGTACCTCGTCCGGCTGCACGCCGGCGAGACC
Ae717Ps2_rpoB        ACCGAGGAGGATGTCGCCACCACCATCGAGTACCTCGTCCGGCTGCACGCCGGCGAGACC
Ae706Ps2_rpoB        ACCGAGGAGGATGTCGCCACCACCATCGAGTACCTCGTCCGGCTGCACGCCGGCGAGACC
Ae150APs1_rpoB       ACCGAGGAGGACGTCGCCACGACCATCGAGTACCTCGTCCGGCTGCACGCCGGCGAGACC
Ae168Ps1_rpoB        ACCGAGGAGGACGTCGCCACGACCATCGAGTACCTCGTCCGGCTGCACGCCGGCGAGACC
Ae263Ps1_rpoB        ACCGAGGAGGACGTCGCCACGACCATCGAGTACCTCGTCCGGCTGCACGCCGGCGAGACC
Ae356Ps1_rpoB        ACCGAGGAGGACGTCGCCACGACCATCGAGTACCTCGTCCGGCTGCACGCCGGCGAGACC
Ae707Ps1_rpoB        ACCGAGGAGGACGTCGCCACGACCATCGAGTACCTCGTCCGGCTGCACGCCGGCGAGACC
                     *********** ******** ***************************************

Ae331Ps2_rpoB        ACGATGACCGTGGGTTCCGGTGACTCCGCGCGGGAGATCCCGGTCGAGACCGACGACATC
Ae505Ps2_rpoB        ACGATGACCGTGGGTTCCGGTGACTCCGCGCGGGAGATCCCGGTCGAGACCGACGACATC
Ae406Ps2_rpoB        ACGATGACCGTGGGTTCCGGTGACTCCGCGCGGGAGATCCCGGTCGAGACCGACGACATC
Ae717Ps2_rpoB        ACGATGACCGTGGGTTCCGGTGACTCCGCGCGGGAGATCCCGGTCGAGACCGACGACATC
Ae706Ps2_rpoB        ACGATGACCGTGGGTTCCGGTGACTCCGCGCGGGAGATCCCGGTCGAGACCGACGACATC
Ae150APs1_rpoB       ACGATGTCGGTGGGCTCCGGCGACTCCGCCCGGGAGATCCCGGTCGAGACCGACGACATC
Ae168Ps1_rpoB        ACGATGTCGGTGGGCTCCGGCGACTCCGCCCGGGAGATCCCGGTCGAGACCGACGACATC
Ae263Ps1_rpoB        ACGATGTCGGTGGGCTCCGGCGACTCCGCCCGGGAGATCCCGGTCGAGACCGACGACATC
Ae356Ps1_rpoB        ACGATGTCGGTGGGCTCCGGCGACTCCGCCCGGGAGATCCCGGTCGAGACCGACGACATC
Ae707Ps1_rpoB        ACGATGTCGGTGGGCTCCGGCGACTCCGCCCGGGAGATCCCGGTCGAGACCGACGACATC
                     ****** * ***** ***** ******** ******************************

Ae331Ps2_rpoB        GACCACTTCGGCAACCGGCGCCTGCGCACCGTCGGCGAGCTGATCCAGAACCAGGTCCGG
Ae505Ps2_rpoB        GACCACTTCGGCAACCGGCGCCTGCGCACCGTCGGCGAGCTGATCCAGAACCAGGTCCGG
Ae406Ps2_rpoB        GACCACTTCGGCAACCGGCGCCTGCGCACCGTCGGCGAGCTGATCCAGAACCAGGTCCGG
Ae717Ps2_rpoB        GACCACTTCGGCAACCGGCGCCTGCGCACCGTCGGCGAGCTGATCCAGAACCAGGTCCGG
Ae706Ps2_rpoB        GACCACTTCGGCAACCGGCGCCTGCGCACCGTCGGCGAGCTGATCCAGAACCAGGTCCGG
Ae150APs1_rpoB       GACCACTTCGGCAACCGGCGTCTGCGTACCGTCGGCGAGCTGATCCAGAACCAGGTGCGG
Ae168Ps1_rpoB        GACCACTTCGGCAACCGGCGTCTGCGTACCGTCGGCGAGCTGATCCAGAACCAGGTGCGG
Ae263Ps1_rpoB        GACCACTTCGGCAACCGGCGTCTGCGTACCGTCGGCGAGCTGATCCAGAACCAGGTGCGG
Ae356Ps1_rpoB        GACCACTTCGGCAACCGGCGTCTGCGTACCGTCGGCGAGCTGATCCAGAACCAGGTGCGG
Ae707Ps1_rpoB        GACCACTTCGGCAACCGGCGTCTGCGTACCGTCGGCGAGCTGATCCAGAACCAGGTGCGG
                     ******************** ***** ***************************** ***

Ae331Ps2_rpoB        GTCGGTCTGTCCCGCACCGAGCGGGTCGTCCGCGAGCGGATGACCACCCAGGACGTCGAG
Ae505Ps2_rpoB        GTCGGTCTGTCCCGCACCGAGCGGGTCGTCCGCGAGCGGATGACCACCCAGGACGTCGAG
Ae406Ps2_rpoB        GTCGGTCTGTCCCGCACCGAGCGGGTCGTCCGCGAGCGGATGACCACCCAGGACGTCGAG
Ae717Ps2_rpoB        GTCGGTCTGTCCCGCACCGAGCGGGTCGTCCGCGAGCGGATGACCACCCAGGACGTCGAG
Ae706Ps2_rpoB        GTCGGTCTGTCCCGCACCGAGCGGGTCGTCCGCGAGCGGATGACCACCCAGGACGTCGAG
Ae150APs1_rpoB       GTCGGTCTGTCGCGCACCGAGCGGGTCGTCCGCGAGCGGATGACCACGCAGGACGTCGAG
Ae168Ps1_rpoB        GTCGGTCTGTCGCGCACCGAGCGGGTCGTCCGCGAGCGGATGACCACGCAGGACGTCGAG
Ae263Ps1_rpoB        GTCGGTCTGTCGCGCACCGAGCGGGTCGTCCGCGAGCGGATGACCACGCAGGACGTCGAG
Ae356Ps1_rpoB        GTCGGTCTGTCGCGCACCGAGCGGGTCGTCCGCGAGCGGATGACCACGCAGGACGTCGAG
Ae707Ps1_rpoB        GTCGGTCTGTCGCGCACCGAGCGGGTCGTCCGCGAGCGGATGACCACGCAGGACGTCGAG
                     *********** *********************************** ************

Ae331Ps2_rpoB        GCCATCACGCCGCAGACCCTGATCAACACCCGGCCGATCACGGCCGCGATCCGGGAGTTC
Ae505Ps2_rpoB        GCCATCACGCCGCAGACCCTGATCAACACCCGGCCGATCACGGCCGCGATCCGGGAGTTC
Ae406Ps2_rpoB        GCCATCACGCCGCAGACCCTGATCAACACCCGGCCGATCACGGCCGCGATCCGGGAGTTC
Ae717Ps2_rpoB        GCCATCACGCCGCAGACCCTGATCAACACCCGGCCGATCACGGCCGCGATCCGGGAGTTC
Ae706Ps2_rpoB        GCCATCACGCCGCAGACCCTGATCAACACCCGGCCGATCACGGCCGCGATCCGGGAGTTC
Ae150APs1_rpoB       GCGATCACGCCGCAGACCCTGATCAACACCCGGCCGATCACGGCCGCGATCCGGGAGTTC
Ae168Ps1_rpoB        GCGATCACGCCGCAGACCCTGATCAACACCCGGCCGATCACGGCCGCGATCCGGGAGTTC
Ae263Ps1_rpoB        GCGATCACGCCGCAGACCCTGATCAACACCCGGCCGATCACGGCCGCGATCCGGGAGTTC
Ae356Ps1_rpoB        GCGATCACGCCGCAGACCCTGATCAACACCCGGCCGATCACGGCCGCGATCCGGGAGTTC
Ae707Ps1_rpoB        GCGATCACGCCGCAGACCCTGATCAACACCCGGCCGATCACGGCCGCGATCCGGGAGTTC
                     ** *********************************************************

Ae331Ps2_rpoB        TTCGGCACCTCGCAGCTGTCGCAGTTCATGGACCAGCACAACCCGCTGGCCGGACTGACC
Ae505Ps2_rpoB        TTCGGCACCTCGCAGCTGTCGCAGTTCATGGACCAGCACAACCCGCTGGCCGGACTGACC
Ae406Ps2_rpoB        TTCGGCACCTCGCAGCTGTCGCAGTTCATGGACCAGCACAACCCGCTGGCCGGACTGACC
```

```
Ae717Ps2_rpoB        TTCGGCACCTCGCAGCTGTCGCAGTTCATGGACCAGCACAACCCGCTGGCCGGACTGACC
Ae706Ps2_rpoB        TTCGGCACCTCGCAGCTGTCGCAGTTCATGGACCAGCACAACCCGCTGGCCGGACTGACC
Ae150APs1_rpoB       TTCGGCACCTCGCAGCTGTCCCAGTTCATGGACCAGCACAACCCCCTCGCGGGCCTGACC
Ae168Ps1_rpoB        TTCGGCACCTCGCAGCTGTCCCAGTTCATGGACCAGCACAACCCCCTCGCGGGCCTGACC
Ae263Ps1_rpoB        TTCGGCACCTCGCAGCTGTCCCAGTTCATGGACCAGCACAACCCCCTCGCGGGCCTGACC
Ae356Ps1_rpoB        TTCGGCACCTCGCAGCTGTCCCAGTTCATGGACCAGCACAACCCCCTCGCGGGCCTGACC
Ae707Ps1_rpoB        TTCGGCACCTCGCAGCTGTCCCAGTTCATGGACCAGCACAACCCCCTCGCGGGCCTGACC
                     ******************** *********************** ** ** ** ******

Ae331Ps2_rpoB        CACAAGCGTCGTCTGTCGGCGCTCGGCCCGGGCGGTCTGTCCCGTGAGCGCGCCGGCATG
Ae505Ps2_rpoB        CACAAGCGTCGTCTGTCGGCGCTCGGCCCGGGCGGTCTGTCCCGTGAGCGCGCCGGCATG
Ae406Ps2_rpoB        CACAAGCGTCGTCTGTCGGCGCTCGGCCCGGGCGGTCTGTCCCGTGAGCGCGCCGGCATG
Ae717Ps2_rpoB        CACAAGCGTCGTCTGTCGGCGCTCGGCCCGGGCGGTCTGTCCCGTGAGCGCGCCGGCATG
Ae706Ps2_rpoB        CACAAGCGTCGTCTGTCGGCGCTCGGCCCGGGCGGTCTGTCCCGTGAGCGCGCCGGCATG
Ae150APs1_rpoB       CACAAGCGCCGCCTGTCGGCGCTCGGCCCGGGTGGTCTGTCGCGTGAGCGTGCGGGCATG
Ae168Ps1_rpoB        CACAAGCGCCGCCTGTCGGCGCTCGGCCCGGGTGGTCTGTCGCGTGAGCGTGCGGGCATG
Ae263Ps1_rpoB        CACAAGCGCCGCCTGTCGGCGCTCGGCCCGGGTGGTCTGTCGCGTGAGCGTGCGGGCATG
Ae356Ps1_rpoB        CACAAGCGCCGCCTGTCGGCGCTCGGCCCGGGTGGTCTGTCGCGTGAGCGTGCGGGCATG
Ae707Ps1_rpoB        CACAAGCGCCGCCTGTCGGCGCTCGGCCCGGGTGGTCTGTCGCGTGAGCGTGCGGGCATG
                     ******** ** ******************** ******** ******** ** ******

Ae331Ps2_rpoB        GAGGTCCGCGACGTCCACCCGTCGCACTACGGCCGCATGTGCCCGATCGAGACCCCCGAG
Ae505Ps2_rpoB        GAGGTCCGCGACGTCCACCCGTCGCACTACGGCCGCATGTGCCCGATCGAGACCCCCGAG
Ae406Ps2_rpoB        GAGGTCCGCGACGTCCACCCGTCGCACTACGGCCGCATGTGCCCGATCGAGACCCCCGAG
Ae717Ps2_rpoB        GAGGTCCGCGACGTCCACCCGTCGCACTACGGCCGCATGTGCCCGATCGAGACCCCCGAG
Ae706Ps2_rpoB        GAGGTCCGCGACGTCCACCCGTCGCACTACGGCCGCATGTGCCCGATCGAGACCCCCGAG
Ae150APs1_rpoB       GAGGTCCGTGACGTCCACCCGTCGCACTACGGCCGGATGTGCCCGATCGAGACCCCGGAG
Ae168Ps1_rpoB        GAGGTCCGTGACGTCCACCCGTCGCACTACGGCCGGATGTGCCCGATCGAGACCCCGGAG
Ae263Ps1_rpoB        GAGGTCCGTGACGTCCACCCGTCGCACTACGGCCGGATGTGCCCGATCGAGACCCCGGAG
Ae356Ps1_rpoB        GAGGTCCGTGACGTCCACCCGTCGCACTACGGCCGGATGTGCCCGATCGAGACCCCGGAG
Ae707Ps1_rpoB        GAGGTCCGTGACGTCCACCCGTCGCACTACGGCCGGATGTGCCCGATCGAGACCCCGGAG
                     ******** ************************** ******************** ***

Ae331Ps2_rpoB        GGTCCGAACATCGGTCTGATCGGCTCGCTGGCCTCTTTCGCGCGGGTCAACCCGTTCGGC
Ae505Ps2_rpoB        GGTCCGAACATCGGTCTGATCGGCTCGCTGGCCTCTTTCGCGCGGGTCAACCCGTTCGGC
Ae406Ps2_rpoB        GGTCCGAACATCGGTCTGATCGGCTCGCTGGCCTCTTTCGCGCGGGTCAACCCGTTCGGC
Ae717Ps2_rpoB        GGTCCGAACATCGGTCTGATCGGCTCGCTGGCCTCTTTCGCGCGGGTCAACCCGTTCGGC
Ae706Ps2_rpoB        GGTCCGAACATCGGTCTGATCGGCTCGCTGGCCTCTTTCGCGCGGGTCAACCCGTTCGGC
Ae150APs1_rpoB       GGGCCGAACATCGGTCTGATCGGGTCGCTCGCCTCCTATGCGCGGGTCAACCCGTTCGGC
Ae168Ps1_rpoB        GGGCCGAACATCGGTCTGATCGGGTCGCTCGCCTCCTATGCGCGGGTCAACCCGTTCGGC
Ae263Ps1_rpoB        GGGCCGAACATCGGTCTGATCGGGTCGCTCGCCTCCTATGCGCGGGTCAACCCGTTCGGC
Ae356Ps1_rpoB        GGGCCGAACATCGGTCTGATCGGGTCGCTCGCCTCCTATGCGCGGGTCAACCCGTTCGGC
Ae707Ps1_rpoB        GGGCCGAACATCGGTCTGATCGGGTCGCTCGCCTCCTACGCGCGGGTCAACCCGTTCGGC
                     ** ******************** ***** ***** *  *********************

Ae331Ps2_rpoB        TTCATCGAGACGCCGTACCGCAAGGTCGTCGACGGTGTCGTCACCTCGCAGATCGACTAC
Ae505Ps2_rpoB        TTCATCGAGACGCCGTACCGCAAGGTCGTCGACGGTGTCGTCACCTCGCAGATCGACTAC
Ae406Ps2_rpoB        TTCATCGAGACGCCGTACCGCAAGGTCGTCGACGGTGTCGTCACCTCGCAGATCGACTAC
Ae717Ps2_rpoB        TTCATCGAGACGCCGTACCGCAAGGTCGTCGACGGTGTCGTCACCTCGCAGATCGACTAC
Ae706Ps2_rpoB        TTCATCGAGACGCCGTACCGCAAGGTCGTCGACGGTGTCGTCACCTCGCAGATCGACTAC
Ae150APs1_rpoB       TTCATCGAGACCCCGTACCGCAAGGTCACCGAGGGTGTCGTCACCGAGCAGATCGACTAC
Ae168Ps1_rpoB        TTCATCGAGACCCCGTACCGCAAGGTCACCGAGGGTGTCGTCACCGAGCAGATCGACTAC
Ae263Ps1_rpoB        TTCATCGAGACCCCGTACCGCAAGGTCACCGAGGGTGTCGTCACCGAGCAGATCGACTAC
Ae356Ps1_rpoB        TTCATCGAGACCCCGTACCGCAAGGTCACCGAGGGTGTCGTCACCGAGCAGATCGACTAC
Ae707Ps1_rpoB        TTCATCGAGACCCCGTACCGCAAGGTCACCGAGGGTGTCGTCACCGAGCAGATCGACTAC
                     *********** ***************  *** ************  *************

Ae331Ps2_rpoB        CTGACCGCCGACGAGGAGGACCGCTTCGTCGTCGCCCAGGCGAACGCCCAGCTCAACGAG
Ae505Ps2_rpoB        CTGACCGCCGACGAGGAGGACCGCTTCGTCGTAGCCCAGGCGAACGCCCAGCTCAACGAG
Ae406Ps2_rpoB        CTGACCGCCGACGAGGAGGACCGCTTCGTCGTCGCCCAGGCGAACGCCCAGCTCAACGAG
Ae717Ps2_rpoB        CTGACCGCCGACGAGGAGGACCGCTTCGTCGTCGCCCAGGCGAACGCCCAGCTCAACGAG
Ae706Ps2_rpoB        CTGACCGCCGACGAGGAGGACCGCTTCGTCGTCGCCCAGGCGAACGCCCAGCTCAACGAG
Ae150APs1_rpoB       CTGACCGCCGACGAGGAGGACCGCTTCGTCGTCGCCCAGGCGAACGCCCGGCTGAACGAG
Ae168Ps1_rpoB        CTGACCGCCGACGAGGAGGACCGCTTCGTCGTCGCCCAGGCGAACGCCCGGCTGAACGAG
Ae263Ps1_rpoB        CTGACCGCCGACGAGGAGGACCGCTTCGTCGTCGCCCAGGCGAACGCCCGGCTGAACGAG
Ae356Ps1_rpoB        CTGACCGCCGACGAGGAGGACCGCTTCGTCGTCGCCCAGGCGAACGCCCGGCTGAACGAG
Ae707Ps1_rpoB        CTGACCGCCGACGAGGAGGACCGCTTCGTCGTCGCCCAGGCGAACGCCCGGCTGAACGAG
                     ******************************** **************** *** ******

Ae331Ps2_rpoB        GACGGTTCCTTCGCCGACGAGCGCGTGCTGGTCCGCCGCAAGGGCGGCGAGGTCGACCTG
Ae505Ps2_rpoB        GACGGTTCCTTCGCCGACGAGCGCGTGCTGGTCCGCCGCAAGGGCGGCGAGGTCGACCTG
Ae406Ps2_rpoB        GACGGTTCCTTCGCCGACGAGCGCGTGCTGGTCCGCCGCAAGGGCGGCGAGGTCGACCTG
Ae717Ps2_rpoB        GACGGTTCCTTCGCCGACGAGCGCGTGCTGGTCCGCCGCAAGGGCGGCGAGGTCGACCTG
Ae706Ps2_rpoB        GACGGTTCCTTCGCCGACGAGCGCGTGCTGGTCCGCCGCAAGGGCGGCGAGGTCGACCTG
Ae150APs1_rpoB       GACGGCTCGTTCGCCGAGGACCGGGTCCTGGTCCGCCGCAAGGGCGGCGAGGTCGACCTG
Ae168Ps1_rpoB        GACGGCTCGTTCGCCGAGGACCGGGTCCTGGTCCGCCGCAAGGGCGGCGAGGTCGACCTG
Ae263Ps1_rpoB        GACGGCTCGTTCGCCGAGGACCGGGTCCTGGTCCGCCGCAAGGGCGGCGAGGTCGACCTG
```

```
Ae356Ps1_rpoB      GACGGCTCGTTCGCCGAGGACCGGGTCCTGGTCCGCCGCAAGGGCGGCGAGGTCGACCTG
Ae707Ps1_rpoB      GACGGCTCGTTCGCCGAGGACCGGGTCCTGGTCCGCCGCAAGGGCGGCGAGGTCGACCTG
                   ***** ** ******** ** ** ** *********************************

Ae331Ps2_rpoB      ATCTCGCCCTCGGGCGTGGAGTACATCGACGTCTCGCCGCGCCAGATGGTGTCGGTCGCG
Ae505Ps2_rpoB      ATCTCGCCCTCGGGCGTGGAGTACATCGACGTCTCGCCGCGCCAGATGGTGTCGGTCGCG
Ae406Ps2_rpoB      ATCTCGCCCTCGGGCGTGGAGTACATCGACGTCTCGCCGCGCCAGATGGTGTCGGTCGCG
Ae717Ps2_rpoB      ATCTCGCCCTCGGGCGTGGAGTACATCGACGTCTCGCCGCGCCAGATGGTGTCGGTCGCG
Ae706Ps2_rpoB      ATCTCGCCCTCGGGCGTGGAGTACATCGACGTCTCGCCGCGCCAGATGGTGTCGGTCGCG
Ae150APs1_rpoB     ATCTCGCCCACCGGCGTCGAGTACATCGACGTCTCGCCGCGCCAGATGGTGTCGGTCGCG
Ae168Ps1_rpoB      ATCTCGCCCACCGGCGTCGAGTACATCGACGTCTCGCCGCGCCAGATGGTGTCGGTCGCG
Ae263Ps1_rpoB      ATCTCGCCCACCGGCGTCGAGTACATCGACGTCTCGCCGCGCCAGATGGTGTCGGTCGCG
Ae356Ps1_rpoB      ATCTCGCCCACCGGCGTCGAGTACATCGACGTCTCGCCGCGCCAGATGGTGTCGGTCGCG
Ae707Ps1_rpoB      ATCTCGCCCACCGGCGTCGAGTACATCGACGTCTCGCCGCGCCAGATGGTGTCGGTCGCG
                   ********* * ***** ******************************************

Ae331Ps2_rpoB      ACGGCGATGATCCCGTTCCTCGAGCACGACGACGCGAACCGCGCGCTGATGGGCGCGAAC
Ae505Ps2_rpoB      ACGGCGATGATCCCGTTCCTCGAGCACGACGACGCGAACCGCGCGCTGATGGGCGCGAAC
Ae406Ps2_rpoB      ACGGCGATGATCCCGTTCCTCGAGCACGACGACGCGAACCGCGCGCTGATGGGCGCGAAC
Ae717Ps2_rpoB      ACGGCGATGATCCCGTTCCTCGAGCACGACGACGCGAACCGCGCGCTGATGGGCGCGAAC
Ae706Ps2_rpoB      ACGGCGATGATCCCGTTCCTCGAGCACGACGACGCGAACCGCGCGCTGATGGGCGCGAAC
Ae150APs1_rpoB     ACCGCGATGATCCCGTTCCTCGAGCACGACGACGCGAACCGCGCCCTCATGGGTGCGAAC
Ae168Ps1_rpoB      ACCGCGATGATCCCGTTCCTCGAGCACGACGACGCGAACCGCGCCCTCATGGGTGCGAAC
Ae263Ps1_rpoB      ACCGCGATGATCCCGTTCCTCGAGCACGACGACGCGAACCGCGCCCTCATGGGTGCGAAC
Ae356Ps1_rpoB      ACCGCGATGATCCCGTTCCTCGAGCACGACGACGCGAACCGCGCCCTCATGGGTGCGAAC
Ae707Ps1_rpoB      ACCGCGATGATCCCGTTCCTCGAGCACGACGACGCGAACCGCGCCCTCATGGGTGCGAAC
                   ** ***************************************** ** ***** ******

Ae331Ps2_rpoB      ATGCAGCGCCAGTCCGTGCCGCTGCTGCGCTCGGAGTCGCCGCTGGTCGGTACCGGCATG
Ae505Ps2_rpoB      ATGCAGCGCCAGTCCGTGCCGCTGCTGCGCTCGGAGTCGCCGCTGGTCGGTACCGGCATG
Ae406Ps2_rpoB      ATGCAGCGCCAGTCCGTGCCGCTGCTGCGCTCGGAGTCGCCGCTGGTCGGTACCGGCATG
Ae717Ps2_rpoB      ATGCAGCGCCAGTCCGTGCCGCTGCTGCGCTCGGAGTCGCCGCTGGTCGGTACCGGCATG
Ae706Ps2_rpoB      ATGCAGCGCCAGTCCGTGCCGCTGCTGCGCTCGGAGTCGCCGCTGGTCGGTACCGGCATG
Ae150APs1_rpoB     ATGCAGCGTCAGTCCGTGCCGCTGCTGCGTTCCGAGTCGCCGCTGGTCGGCACCGGCATG
Ae168Ps1_rpoB      ATGCAGCGTCAGTCCGTGCCGCTGCTGCGTTCCGAGTCGCCGCTGGTCGGCACCGGCATG
Ae263Ps1_rpoB      ATGCAGCGTCAGTCCGTGCCGCTGCTGCGTTCCGAGTCGCCGCTGGTCGGCACCGGCATG
Ae356Ps1_rpoB      ATGCAGCGTCAGTCCGTGCCGCTGCTGCGTTCCGAGTCGCCGCTGGTCGGCACCGGCATG
Ae707Ps1_rpoB      ATGCAGCGTCAGTCCGTGCCGCTGCTGCGTTCCGAGTCGCCGCTGGTCGGCACCGGCATG
                   ******** ******************** ** ***************** *********

Ae331Ps2_rpoB      GAGCTGCGTGCCGCCGTCGACGCCGGTGACGTCGTCACCGCCGAGTCCGCCGGTGTCGTC
Ae505Ps2_rpoB      GAGCTGCGTGCCGCCGTCGACGCCGGTGACGTCGTCACCGCCGAGTCCGCCGGTGTCGTC
Ae406Ps2_rpoB      GAGCTGCGTGCCGCCGTCGACGCCGGTGACGTCGTCACCGCCGAGTCCGCCGGTGTCGTC
Ae717Ps2_rpoB      GAGCTGCGTGCCGCCGTCGACGCCGGTGACGTCGTCACCGCCGAGTCCGCCGGTGTCGTC
Ae706Ps2_rpoB      GAGCTGCGTGCCGCCGTCGACGCCGGTGACGTCGTCACCGCCGAGTCCGCCGGTGTCGTC
Ae150APs1_rpoB     GAGCTGCGGGCCGCGGTCGACGCCGGCGACGTCGTGGTGGCCGAGCAGGCCGGTGTGGTC
Ae168Ps1_rpoB      GAGCTGCGGGCCGCGGTCGACGCCGGCGACGTCGTGGTGGCCGAGCAGGCCGGTGTGGTC
Ae263Ps1_rpoB      GAGCTGCGGGCCGCGGTCGACGCCGGCGACGTCGTGGTGGCCGAGCAGGCCGGTGTGGTC
Ae356Ps1_rpoB      GAGCTGCGGGCCGCGGTCGACGCCGGCGACGTCGTGGTGGCCGAGCAGGCCGGTGTGGTC
Ae707Ps1_rpoB      GAGCTGCGGGCCGCGGTCGACGCCGGCGACGTCGTGGTGGCCGAGCAGGCCGGTGTGGTC
                   ******** ***** *********** ********    ******   ******** ***

Ae331Ps2_rpoB      GAGGAGCTGTGCGCCGACTACATCACCGTCATGGACGACGAGGGCCAGCGTCAGACCTAC
Ae505Ps2_rpoB      GAGGAGCTGTGCGCCGACTACATCACCGTCATGGACGACGAGGGCCAGCGTCAGACCTAC
Ae406Ps2_rpoB      GAGGAGCTGTGCGCCGACTACATCACCGTCATGGACGACGAGGGCCAGCGTCAGACCTAC
Ae717Ps2_rpoB      GAGGAGCTGTGCGCCGACTACATCACCGTCATGGACGACGAGGGCCAGCGTCAGACCTAC
Ae706Ps2_rpoB      GAGGAGCTGTGCGCCGACTACATCACCGTCATGGACGACGAGGGCCAGCGTCAGACCTAC
Ae150APs1_rpoB     GAGGAGCTGTGCGCCGACTACATCACCGTCATGGACGACGAGGGGCAGCGTCAGACCTAC
Ae168Ps1_rpoB      GAGGAGCTGTGCGCCGACTACATCACCGTCATGGACGACGAGGGGCAGCGTCAGACCTAC
Ae263Ps1_rpoB      GAGGAGCTGTGCGCCGACTACATCACCGTCATGGACGACGAGGGGCAGCGTCAGACCTAC
Ae356Ps1_rpoB      GAGGAGCTGTGCGCCGACTACATCACCGTCATGGACGACGAGGGGCAGCGTCAGACCTAC
Ae707Ps1_rpoB      GAGGAGCTGTGCGCCGACTACATCACCGTCATGGACGACGAGGGGCAGCGTCAGACCTAC
                   ******************************************** ***************

Ae331Ps2_rpoB      CGTCTGAACAAGTTCCGCCGCTCGAACCAGGGCACCTGCAACAACCAGAAGCCCATCGTC
Ae505Ps2_rpoB      CGTCTGAACAAGTTCCGCCGCTCGAACCAGGGCACCTGCAACAACCAGAAGCCCATCGTC
Ae406Ps2_rpoB      CGTCTGAACAAGTTCCGCCGCTCGAACCAGGGCACCTGCAACAACCAGAAGCCCATCGTC
Ae717Ps2_rpoB      CGTCTGAACAAGTTCCGCCGCTCGAACCAGGGCACCTGCAACAACCAGAAGCCCATCGTC
Ae706Ps2_rpoB      CGTCTGAACAAGTTCCGCCGCTCGAACCAGGGCACCTGCAACAACCAGAAGCCCATCGTC
Ae150APs1_rpoB     CGTCTGAACAAGTTCCGCCGCTCGAACCAGGGCACCTGCAACAACCAGAAGCCGATCGTC
Ae168Ps1_rpoB      CGTCTGAACAAGTTCCGCCGCTCGAACCAGGGCACCTGCAACAACCAGAAGCCGATCGTC
Ae263Ps1_rpoB      CGTCTGAACAAGTTCCGCCGCTCGAACCAGGGCACCTGCAACAACCAGAAGCCGATCGTC
Ae356Ps1_rpoB      CGTCTGAACAAGTTCCGCCGCTCGAACCAGGGCACCTGCAACAACCAGAAGCCGATCGTC
Ae707Ps1_rpoB      CGTCTGAACAAGTTCCGCCGCTCGAACCAGGGCACCTGCAACAACCAGAAGCCGATCGTC
                   ***************************************************** ******

Ae331Ps2_rpoB      GACGAGGGGCAGCGGGTCGAGGTGGGCCAGGTGCTCGCCGACGGTCCCTGCACCGAGAAC
```

```
Ae505Ps2_rpoB      GACGAGGGGCAGCGGGTCGAGGTGGGCCAGGTGCTCGCCGACGGTCCCTGCACCGAGAAC
Ae406Ps2_rpoB      GACGAGGGGCAGCGGGTCGAGGTGGGCCAGGTGCTCGCCGACGGTCCCTGCACCGAGAAC
Ae717Ps2_rpoB      GACGAGGGGCAGCGGGTCGAGGTGGGCCAGGTGCTCGCCGACGGTCCCTGCACCGAGAAC
Ae706Ps2_rpoB      GACGAGGGGCAGCGGGTCGAGGTGGGCCAGGTGCTCGCCGACGGTCCCTGCACCGAGAAC
Ae150APs1_rpoB     GACGAGGGCATGCGGGTCGAGGTGGGCCAGGTGCTCGCCGACGGACCGTGCACCGAGAAC
Ae168Ps1_rpoB      GACGAGGGCATGCGGGTCGAGGTGGGCCAGGTGCTCGCCGACGGACCGTGCACCGAGAAC
Ae263Ps1_rpoB      GACGAGGGCATGCGGGTCGAGGTGGGCCAGGTGCTCGCCGACGGACCGTGCACCGAGAAC
Ae356Ps1_rpoB      GACGAGGGCATGCGGGTCGAGGTGGGCCAGGTGCTCGCCGACGGACCGTGCACCGAGAAC
Ae707Ps1_rpoB      GACGAGGGCATGCGGGTCGAGGTGGGCCAGGTGCTCGCCGACGGACCGTGCACCGAGAAC
                   ********   ********************************* ** ************

Ae331Ps2_rpoB      GGTGAGATGGCGCTGGGCAAGAACCTGCTCGTGGCGATCATGCCGTGGGAGGGCCACAAC
Ae505Ps2_rpoB      GGTGAGATGGCGCTGGGCAAGAACCTGCTCGTGGCGATCATGCCGTGGGAGGGCCACAAC
Ae406Ps2_rpoB      GGTGAGATGGCGCTGGGCAAGAACCTGCTCGTGGCGATCATGCCGTGGGAGGGCCACAAC
Ae717Ps2_rpoB      GGTGAGATGGCGCTGGGCAAGAACCTGCTCGTGGCGATCATGCCGTGGGAGGGCCACAAC
Ae706Ps2_rpoB      GGTGAGATGGCGCTGGGCAAGAACCTGCTCGTGGCGATCATGCCGTGGGAGGGCCACAAC
Ae150APs1_rpoB     GGCGAGATGGCGCTGGGCAAGAACCTCCTCGTGGCGATCATGCCGTGGGAGGGCCACAAC
Ae168Ps1_rpoB      GGCGAGATGGCGCTGGGCAAGAACCTCCTCGTGGCGATCATGCCGTGGGAGGGCCACAAC
Ae263Ps1_rpoB      GGCGAGATGGCGCTGGGCAAGAACCTCCTCGTGGCGATCATGCCGTGGGAGGGCCACAAC
Ae356Ps1_rpoB      GGCGAGATGGCGCTGGGCAAGAACCTCCTCGTGGCGATCATGCCGTGGGAGGGCCACAAC
Ae707Ps1_rpoB      GGCGAGATGGCGCTGGGCAAGAACCTCCTCGTGGCGATCATGCCGTGGGAGGGCCACAAC
                   ** *********************** *********************************

Ae331Ps2_rpoB      TACGAGGACGCGATCATCCTGTCGCAGCGCCTCGTGCAGGACGACGTGCTCACCTCGATC
Ae505Ps2_rpoB      TACGAGGACGCGATCATCCTGTCGCAGCGCCTCGTGCAGGACGACGTGCTCACCTCGATC
Ae406Ps2_rpoB      TACGAGGACGCGATCATCCTGTCGCAGCGCCTCGTGCAGGACGACGTGCTCACCTCGATC
Ae717Ps2_rpoB      TACGAGGACGCGATCATCCTGTCGCAGCGCCTCGTGCAGGACGACGTGCTCACCTCGATC
Ae706Ps2_rpoB      TACGAGGACGCGATCATCCTGTCGCAGCGCCTCGTGCAGGACGACGTGCTCACCTCGATC
Ae150APs1_rpoB     TACGAGGACGCGATCATCCTGTCGCAGCGCCTCGTCCAGGACGACGTCCTGACCTCGATC
Ae168Ps1_rpoB      TACGAGGACGCGATCATCCTGTCGCAGCGCCTCGTCCAGGACGACGTCCTGACCTCGATC
Ae263Ps1_rpoB      TACGAGGACGCGATCATCCTGTCGCAGCGCCTCGTCCAGGACGACGTCCTGACCTCGATC
Ae356Ps1_rpoB      TACGAGGACGCGATCATCCTGTCGCAGCGCCTCGTCCAGGACGACGTCCTGACCTCGATC
Ae707Ps1_rpoB      TACGAGGACGCGATCATCCTGTCGCAGCGCCTCGTCCAGGACGACGTCCTGACCTCGATC
                   *********************************** *********** ** *********

Ae331Ps2_rpoB      CACATCGAGGAGCACGAGATCGACGCGCGCGACACGAAGCTGGGCGCCGAGGAGATCACC
Ae505Ps2_rpoB      CACATCGAGGAGCACGAGATCGACGCGCGCGACACGAAGCTGGGCGCCGAGGAGATCACC
Ae406Ps2_rpoB      CACATCGAGGAGCACGAGATCGACGCGCGCGACACGAAGCTGGGCGCCGAGGAGATCACC
Ae717Ps2_rpoB      CACATCGAGGAGCACGAGATCGACGCGCGCGACACGAAGCTGGGCGCCGAGGAGATCACC
Ae706Ps2_rpoB      CACATCGAGGAGCACGAGATCGACGCGCGCGACACGAAGCTGGGCGCCGAGGAGATCACC
Ae150APs1_rpoB     CACATCGAGGAGCATGAGGTCGACGCCCGTGACACCAAGCTGGGCGCCGAGGAGATCACC
Ae168Ps1_rpoB      CACATCGAGGAGCATGAGGTCGACGCCCGTGACACCAAGCTGGGCGCCGAGGAGATCACC
Ae263Ps1_rpoB      CACATCGAGGAGCATGAGGTCGACGCCCGTGACACCAAGCTGGGCGCCGAGGAGATCACC
Ae356Ps1_rpoB      CACATCGAGGAGCATGAGGTCGACGCCCGTGACACCAAGCTGGGCGCCGAGGAGATCACC
Ae707Ps1_rpoB      CACATCGAGGAGCACGAGGTCGACGCCCGTGACACCAAGCTGGGCGCCGAGGAGATCACC
                   ************** *** ******* ** ***** ************************

Ae331Ps2_rpoB      CGGGACATCCCGAACGTCTCCGAGGACGTGCTGGCCGACCTCGACGAGCGCGGCATCATC
Ae505Ps2_rpoB      CGGGACATCCCGAACGTCTCCGAGGACGTGCTGGCCGACCTCGACGAGCGCGGCATCATC
Ae406Ps2_rpoB      CGGGACATCCCGAACGTCTCCGAGGACGTGCTGGCCGACCTCGACGAGCGCGGCATCATC
Ae717Ps2_rpoB      CGGGACATCCCGAACGTCTCCGAGGACGTGCTGGCCGACCTCGACGAGCGCGGCATCATC
Ae706Ps2_rpoB      CGGGACATCCCGAACGTCTCCGAGGACGTGCTGGCCGACCTCGACGAGCGCGGCATCATC
Ae150APs1_rpoB     CGGGACATCCCGAACGTCTCCGAGGACGTGCTCGCCGACCTCGACGAGCGCGGCATCATC
Ae168Ps1_rpoB      CGGGACATCCCGAACGTCTCCGAGGACGTGCTCGCCGACCTCGACGAGCGCGGCATCATC
Ae263Ps1_rpoB      CGGGACATCCCGAACGTCTCCGAGGACGTGCTCGCCGACCTCGACGAGCGCGGCATCATC
Ae356Ps1_rpoB      CGGGACATCCCGAACGTCTCCGAGGACGTGCTCGCCGACCTCGACGAGCGCGGCATCATC
Ae707Ps1_rpoB      CGGGACATCCCGAACGTCTCCGAGGACGTGCTCGCCGACCTCGACGAGCGCGGCATCATC
                   ******************************** ***************************

Ae331Ps2_rpoB      CGGATCGGCGCCGAGGTCCAGCCCGGCGACATCCTGGTCGGCAAGGTCACGCCCAAGGGC
Ae505Ps2_rpoB      CGGATCGGCGCCGAGGTCCAGCCCGGCGACATCCTGGTCGGCAAGGTCACGCCCAAGGGC
Ae406Ps2_rpoB      CGGATCGGCGCCGAGGTCCAGCCCGGCGACATCCTGGTCGGCAAGGTCACGCCCAAGGGC
Ae717Ps2_rpoB      CGGATCGGCGCCGAGGTCCAGCCCGGCGACATCCTGGTCGGCAAGGTCACGCCCAAGGGC
Ae706Ps2_rpoB      CGGATCGGCGCCGAGGTCCAGCCCGGCGACATCCTGGTCGGCAAGGTCACGCCCAAGGGC
Ae150APs1_rpoB     CGGATCGGTGCCGAGGTCCAGCCCGGCGACATCCTGGTCGGCAAGGTCACGCCGAAGGGC
Ae168Ps1_rpoB      CGGATCGGTGCCGAGGTCCAGCCCGGCGACATCCTGGTCGGCAAGGTCACGCCGAAGGGC
Ae263Ps1_rpoB      CGGATCGGTGCCGAGGTCCAGCCCGGCGACATCCTGGTCGGCAAGGTCACGCCGAAGGGC
Ae356Ps1_rpoB      CGGATCGGTGCCGAGGTCCAGCCCGGCGACATCCTGGTCGGCAAGGTCACGCCGAAGGGC
Ae707Ps1_rpoB      CGGATCGGTGCCGAGGTCCAGCCCGGCGACATCCTGGTCGGCAAGGTCACGCCGAAGGGC
                   ******** ******************************************** ******

Ae331Ps2_rpoB      GAGACCGAGCTGACCCCGGAGGAGCGCCTGCTCCGCGCGATCTTCGGTGAGAAGGCGCGC
Ae505Ps2_rpoB      GAGACCGAGCTGACCCCGGAGGAGCGCCTGCTCCGCGCGATCTTCGGTGAGAAGGCGCGC
Ae406Ps2_rpoB      GAGACCGAGCTGACCCCGGAGGAGCGCCTGCTCCGCGCGATCTTCGGTGAGAAGGCGCGC
Ae717Ps2_rpoB      GAGACCGAGCTGACCCCGGAGGAGCGCCTGCTCCGCGCGATCTTCGGTGAGAAGGCGCGC
Ae706Ps2_rpoB      GAGACCGAGCTGACCCCGGAGGAGCGCCTGCTCCGCGCGATCTTCGGTGAGAAGGCGCGC
Ae150APs1_rpoB     GAGACCGAGCTGACCCCGGAGGAGCGCCTGCTCCGCGCGATCTTCGGTGAGAAGGCGCGC
```

```
Ae168Ps1_rpoB         GAGACCGAGCTGACCCCGGAGGAGCGCCTGCTCCGCGCGATCTTCGGTGAGAAGGCGCGC
Ae263Ps1_rpoB         GAGACCGAGCTGACCCCGGAGGAGCGCCTGCTCCGCGCGATCTTCGGTGAGAAGGCGCGC
Ae356Ps1_rpoB         GAGACCGAGCTGACCCCGGAGGAGCGCCTGCTCCGCGCGATCTTCGGTGAGAAGGCGCGC
Ae707Ps1_rpoB         GAGACCGAGCTGACCCCGGAGGAGCGCCTGCTCCGCGCGATCTTCGGTGAGAAGGCGCGC
                      ************************************************************

Ae331Ps2_rpoB         GAGGTCCGCGACACCTCGCTGAAGGTCCCGCACGGCGAGAACGGCAAGGTCATCGGCATC
Ae505Ps2_rpoB         GAGGTCCGCGACACCTCGCTGAAGGTCCCGCACGGCGAGAACGGCAAGGTCATCGGCATC
Ae406Ps2_rpoB         GAGGTCCGCGACACCTCGCTGAAGGTCCCGCACGGCGAGAACGGCAAGGTCATCGGCATC
Ae717Ps2_rpoB         GAGGTCCGCGACACCTCGCTGAAGGTCCCGCACGGCGAGAACGGCAAGGTCATCGGCATC
Ae706Ps2_rpoB         GAGGTCCGCGACACCTCGCTGAAGGTCCCGCACGGCGAGAACGGCAAGGTCATCGGCATC
Ae150APs1_rpoB        GAGGTCCGCGACACCTCGCTGAAGGTGCCGCACGGGGAGAACGGCAAGGTCATCGGCATC
Ae168Ps1_rpoB         GAGGTCCGCGACACCTCGCTGAAGGTGCCGCACGGGGAGAACGGCAAGGTCATCGGCATC
Ae263Ps1_rpoB         GAGGTCCGCGACACCTCGCTGAAGGTGCCGCACGGGGAGAACGGCAAGGTCATCGGCATC
Ae356Ps1_rpoB         GAGGTCCGCGACACCTCGCTGAAGGTGCCGCACGGGGAGAACGGCAAGGTCATCGGCATC
Ae707Ps1_rpoB         GAGGTCCGCGACACCTCGCTGAAGGTGCCGCACGGCGAGAACGGCAAGGTCATCGGCATC
                      ************************** ******** ************************

Ae331Ps2_rpoB         CGGGTCTTCTCCCGCGACGACGACGACGAGCTGGCCCCGGGCGTCAACGAGCTGGTCCGC
Ae505Ps2_rpoB         CGGGTCTTCTCCCGCGACGACGACGACGAGCTGGCCCCGGGCGTCAACGAGCTGGTCCGC
Ae406Ps2_rpoB         CGGGTCTTCTCCCGCGACGACGACGACGAGCTGGCCCCGGGCGTCAACGAGCTGGTCCGC
Ae717Ps2_rpoB         CGGGTCTTCTCCCGCGACGACGACGACGAGCTGGCCCCGGGCGTCAACGAGCTGGTCCGC
Ae706Ps2_rpoB         CGGGTCTTCTCCCGCGACGACGACGACGAGCTGGCCCCGGGCGTCAACGAGCTGGTCCGC
Ae150APs1_rpoB        CGGGTCTTCTCCCGCGACGACGACGACGAGCTGGCCCCGGGCGTCAACGAGCTGGTCCGG
Ae168Ps1_rpoB         CGGGTCTTCTCCCGCGACGACGACGACGAGCTGGCCCCGGGCGTCAACGAGCTGGTCCGG
Ae263Ps1_rpoB         CGGGTCTTCTCCCGCGACGACGACGACGAGCTGGCCCCGGGCGTCAACGAGCTGGTCCGG
Ae356Ps1_rpoB         CGGGTCTTCTCCCGCGACGACGACGACGAGCTGGCCCCGGGCGTCAACGAGCTGGTCCGG
Ae707Ps1_rpoB         CGGGTCTTCTCCCGCGACGACGACGACGAGCTGGCCCCGGGCGTCAACGAGCTGGTCCGG
                      *********************************************************** 

Ae331Ps2_rpoB         GTCTACGTGGCCCAGAAGCGCAAGATCTCCGACGGCGACAAGCTCGCCGGCCGGCACGGC
Ae505Ps2_rpoB         GTCTACGTGGCCCAGAAGCGCAAGATCTCCGACGGCGACAAGCTCGCCGGCCGGCACGGC
Ae406Ps2_rpoB         GTCTACGTGGCCCAGAAGCGCAAGATCTCCGACGGCGACAAGCTCGCCGGCCGGCACGGC
Ae717Ps2_rpoB         GTCTACGTGGCCCAGAAGCGCAAGATCTCCGACGGCGACAAGCTCGCCGGCCGGCACGGC
Ae706Ps2_rpoB         GTCTACGTGGCCCAGAAGCGCAAGATCTCCGACGGCGACAAGCTCGCCGGCCGGCACGGC
Ae150APs1_rpoB        GTCTACGTGGCCCAGAAGCGCAAGATCTCCGACGGTGACAAGCTCGCCGGCCGCCACGGC
Ae168Ps1_rpoB         GTCTACGTGGCCCAGAAGCGCAAGATCTCCGACGGTGACAAGCTCGCCGGCCGCCACGGC
Ae263Ps1_rpoB         GTCTACGTGGCCCAGAAGCGCAAGATCTCCGACGGTGACAAGCTCGCCGGCCGCCACGGC
Ae356Ps1_rpoB         GTCTACGTGGCCCAGAAGCGCAAGATCTCCGACGGTGACAAGCTCGCCGGCCGCCACGGC
Ae707Ps1_rpoB         GTCTACGTGGCCCAGAAGCGCAAGATCTCCGACGGTGACAAGCTCGCCGGCCGCCACGGC
                      *********************************** ***************** ******

Ae331Ps2_rpoB         AACAAGGGCGTCATCGGCAAGATCCTCCCGCAGGAGGACATGCCGTTCCTCCCGGACGGC
Ae505Ps2_rpoB         AACAAGGGCGTCATCGGCAAGATCCTCCCGCAGGAGGACATGCCGTTCCTCCCGGACGGC
Ae406Ps2_rpoB         AACAAGGGCGTCATCGGCAAGATCCTCCCGCAGGAGGACATGCCGTTCCTCCCGGACGGC
Ae717Ps2_rpoB         AACAAGGGCGTCATCGGCAAGATCCTCCCGCAGGAGGACATGCCGTTCCTCCCGGACGGC
Ae706Ps2_rpoB         AACAAGGGCGTCATCGGCAAGATCCTCCCGCAGGAGGACATGCCGTTCCTCCCGGACGGC
Ae150APs1_rpoB        AACAAGGGCGTCATCGGCAAGATCCTTCCGGCCGAGGACATGCCGTTCCTCCCGGACGGC
Ae168Ps1_rpoB         AACAAGGGCGTCATCGGCAAGATCCTTCCGGCCGAGGACATGCCGTTCCTCCCGGACGGC
Ae263Ps1_rpoB         AACAAGGGCGTCATCGGCAAGATCCTTCCGGCCGAGGACATGCCGTTCCTCCCGGACGGC
Ae356Ps1_rpoB         AACAAGGGCGTCATCGGCAAGATCCTTCCGGCCGAGGACATGCCGTTCCTCCCGGACGGC
Ae707Ps1_rpoB         AACAAGGGCGTCATCGGCAAGATCCTTCCGGCCGAGGACATGCCGTTCCTCCCGGACGGC
                      ************************** ***   ***************************

Ae331Ps2_rpoB         ACGCCGGTCGACATCATCCTGAACACCCACGGTGTGCCGCGTCGTATGAACATCGGCCAG
Ae505Ps2_rpoB         ACGCCGGTCGACATCATCCTGAACACCCACGGTGTGCCGCGTCGTATGAACATCGGCCAG
Ae406Ps2_rpoB         ACGCCGGTCGACATCATCCTGAACACCCACGGTGTGCCGCGTCGTATGAACATCGGCCAG
Ae717Ps2_rpoB         ACGCCGGTCGACATCATCCTGAACACCCACGGTGTGCCGCGTCGTATGAACATCGGCCAG
Ae706Ps2_rpoB         ACGCCGGTCGACATCATCCTGAACACCCACGGTGTGCCGCGTCGTATGAACATCGGCCAG
Ae150APs1_rpoB        ACGCCGGTCGACATCATCCTGAACACCCACGGTGTGCCGCGTCGTATGAACATCGGCCAG
Ae168Ps1_rpoB         ACGCCGGTCGACATCATCCTGAACACCCACGGTGTGCCGCGTCGTATGAACATCGGCCAG
Ae263Ps1_rpoB         ACGCCGGTCGACATCATCCTGAACACCCACGGTGTGCCGCGTCGTATGAACATCGGCCAG
Ae356Ps1_rpoB         ACGCCGGTCGACATCATCCTGAACACCCACGGTGTGCCGCGTCGTATGAACATCGGCCAG
Ae707Ps1_rpoB         ACGCCGGTCGACATCATCCTGAACACCCACGGTGTGCCGCGTCGTATGAACATCGGCCAG
                      ************************************************************

Ae331Ps2_rpoB         ATCCTGGAGACCCACCTCGGGTGGATCGCCAAGTCCGGCTGGGAGATCGAGGGCAAGCCC
Ae505Ps2_rpoB         ATCCTGGAGACCCACCTCGGGTGGATCGCCAAGTCCGGCTGGGAGATCGAGGGCAAGCCC
Ae406Ps2_rpoB         ATCCTGGAGACCCACCTCGGGTGGATCGCCAAGTCCGGCTGGGAGATCGAGGGCAAGCCC
Ae717Ps2_rpoB         ATCCTGGAGACCCACCTCGGGTGGATCGCCAAGTCCGGCTGGGAGATCGAGGGCAAGCCC
Ae706Ps2_rpoB         ATCCTGGAGACCTACCTCGGGTGGATCGCCAAGTCCGGCTGGGAGATCGAGGGCAAGCCC
Ae150APs1_rpoB        ATCCTGGAGACCCACCTCGGGTGGATCGCCAAGACCGGCTGGGAGATCGAGGGGCAGCCC
Ae168Ps1_rpoB         ATCCTGGAGACCCACCTCGGGTGGATCGCCAAGACCGGCTGGGAGATCGAGGGGCAGCCC
Ae263Ps1_rpoB         ATCCTGGAGACCCACCTCGGGTGGATCGCCAAGACCGGCTGGGAGATCGAGGGGCAGCCC
Ae356Ps1_rpoB         ATCCTGGAGACCCACCTCGGGTGGATCGCCAAGACCGGCTGGGAGATCGAGGGGCAGCCC
Ae707Ps1_rpoB         ATCCTGGAGACCCACCTCGGGTGGATCGCCAAGACCGGCTGGGAGATCGAGGGGCAGCCC
                      ************ ******************** *******************  *****
```

```
Ae331Ps2_rpoB       GAGTGGGCGGCGCAGATGCCCGAGGAGCTCTACTCGGTGCCCGCCGACACGAACACCGCG
Ae505Ps2_rpoB       GAGTGGGCGGCGCAGATGCCCGAGGAGCTCTACTCGGTGCCCGCCGACACGAACACCGCG
Ae406Ps2_rpoB       GAGTGGGCGGCGCAGATGCCCGAGGAGCTCTACTCGGTGCCCGCCGACACGAACACCGCG
Ae717Ps2_rpoB       GAGTGGGCGGCGCAGATGCCCGAGGAGCTCTACTCGGTGCCCGCCGACACGAACACCGCG
Ae706Ps2_rpoB       GAGTGGGCGGCGCAGATGCCCGAGGAGCTCTACTCGGTGCCCGCCGACACGAACACCGCG
Ae150APs1_rpoB      GAATGGGCGTCCCGGATGCCCGAGGAGCTCTACTCGGTGCCCGCCGACACGAAGACCGCG
Ae168Ps1_rpoB       GAATGGGCGTCCCGGATGCCCGAGGAGCTCTACTCGGTGCCCGCCGACACGAAGACCGCG
Ae263Ps1_rpoB       GAATGGGCGTCCCGGATGCCCGAGGAGCTCTACTCGGTGCCCGCCGACACGAAGACCGCG
Ae356Ps1_rpoB       GAATGGGCGTCCCGGATGCCCGAGGAGCTCTACTCGGTGCCCGCCGACACGAAGACCGCG
Ae707Ps1_rpoB       GAATGGGCGTCCCGGATGCCCGAGGAGCTCTACTCGGTGCCCGCCGACACGAAGACCGCG
                    ** ****** * * *************************************** ******

Ae331Ps2_rpoB       ACGCCGGTGTTCGACGGTGCGCGCGAGCACGAGATCACGGGTCTGCTCGGCTCGACCCGC
Ae505Ps2_rpoB       ACGCCGGTGTTCGACGGTGCGCGCGAGCACGAGATCACGGGTCTGCTCGGCTCGACCCGC
Ae406Ps2_rpoB       ACGCCGGTGTTCGACGGTGCGCGCGAGCACGAGATCACGGGTCTGCTCGGCTCGACCCGC
Ae717Ps2_rpoB       ACGCCGGTGTTCGACGGTGCGCGCGAGCACGAGATCACGGGTCTGCTCGGCTCGACCCGC
Ae706Ps2_rpoB       ACGCCGGTGTTCGACGGTGCGCGCGAGCACGAGATCACGGGTCTGCTCGGCTCGACCCGC
Ae150APs1_rpoB      ACGCCGGTCTTCGACGGTGCGCGCGAGCACGAGATCACCGGGCTGCTCGGCAGCACCCTC
Ae168Ps1_rpoB       ACGCCGGTCTTCGACGGTGCGCGCGAGCACGAGATCACCGGGCTGCTCGGCAGCACCCTC
Ae263Ps1_rpoB       ACGCCGGTCTTCGACGGTGCGCGCGAGCACGAGATCACCGGGCTGCTCGGCAGCACCCTC
Ae356Ps1_rpoB       ACGCCGGTCTTCGACGGTGCGCGCGAGCACGAGATCACCGGGCTGCTCGGCAGCACCCTC
Ae707Ps1_rpoB       ACGCCGGTCTTCGACGGTGCGCGCGAGCACGAGATCACCGGGCTGCTCGGCAGCACCCTC
                    ******** ***************************** ** *********   **** *

Ae331Ps2_rpoB       CCCAACCGGGACGGCGAGCGGATGGTCGGTCCGGACGGCAAGGCGCAGCTGCTCGACGGC
Ae505Ps2_rpoB       CCCAACCGGGACGGCGAGCGGATGGTCGGTCCGGACGGCAAGGCGCAGCTGCTCGACGGC
Ae406Ps2_rpoB       CCCAACCGGGACGGCGAGCGGATGGTCGGTCCGGACGGCAAGGCGCAGCTGCTCGACGGC
Ae717Ps2_rpoB       CCCAACCGGGACGGCGAGCGGATGGTCGGTCCGGACGGCAAGGCGCAGCTGCTCGACGGC
Ae706Ps2_rpoB       CCCAACCGGGACGGCGAGCGGATGGTCGGTCCGGACGGCAAGGCGCAGCTGCTCGACGGC
Ae150APs1_rpoB      CCGAACCGCGACGGTGAGCGGATGGTGCAGCCGGACGGCAAGGCGCAGCTGCTCGACGGG
Ae168Ps1_rpoB       CCGAACCGCGACGGTGAGCGGATGGTGCAGCCGGACGGCAAGGCGCAGCTGCTCGACGGG
Ae263Ps1_rpoB       CCGAACCGCGACGGTGAGCGGATGGTGCAGCCGGACGGCAAGGCGCAGCTGCTCGACGGG
Ae356Ps1_rpoB       CCGAACCGCGACGGTGAGCGGATGGTGCAGCCGGACGGCAAGGCGCAGCTGCTCGACGGG
Ae707Ps1_rpoB       CCGAACCGCGACGGTGAGCGGATGGTGCAGCCGGACGGCAAGGCCCAGCTGCTCGACGGG
                    ** ***** ***** ***********    ************** ************** 

Ae331Ps2_rpoB       CGCTCCGGTGAGCCGTACCCGTTCCCGGTCGCGGTCGGCTACATGTACATCCTGAAGCTG
Ae505Ps2_rpoB       CGCTCCGGTGAGCCGTACCCGTTCCCGGTCGCGGTCGGCTACATGTACATCCTGAAGCTG
Ae406Ps2_rpoB       CGCTCCGGTGAGCCGTACCCGTTCCCGGTCGCGGTCGGCTACATGTACATCCTGAAGCTG
Ae717Ps2_rpoB       CGCTCCGGTGAGCCGTACCCGTTCCCGGTCGCGGTCGGCTACATGTACATCCTGAAGCTG
Ae706Ps2_rpoB       CGCTCCGGTGAGCCGTACCCGTTCCCGGTCGCGGTCGGCTACATGTACATCCTGAAGCTG
Ae150APs1_rpoB      CGCTCCGGCGAGCCCTACCCGTTCCCGGTCGCGGTCGGCTACATGTACATCCTGAAGCTG
Ae168Ps1_rpoB       CGCTCCGGCGAGCCCTACCCGTTCCCGGTCGCGGTCGGCTACATGTACATCCTGAAGCTG
Ae263Ps1_rpoB       CGCTCCGGCGAGCCCTACCCGTTCCCGGTCGCGGTCGGCTACATGTACATCCTGAAGCTG
Ae356Ps1_rpoB       CGCTCCGGCGAGCCCTACCCGTTCCCGGTCGCGGTCGGCTACATGTACATCCTGAAGCTG
Ae707Ps1_rpoB       CGCTCCGGCGAGCCGTACCCGTTCCCGGTCGCGGTCGGCTACATGTACATCCTGAAGCTG
                    ******** ***** *********************************************

Ae331Ps2_rpoB       GCCCACCTGGTGGACGACAAGATCCACGCCCGGTCGACCGGCCCCTACTCGATGATCACC
Ae505Ps2_rpoB       GCCCACCTGGTGGACGACAAGATCCACGCCCGGTCGACCGGCCCCTACTCGATGATCACC
Ae406Ps2_rpoB       GCCCACCTGGTGGACGACAAGATCCACGCCCGGTCGACCGGCCCCTACTCGATGATCACC
Ae717Ps2_rpoB       GCCCACCTGGTGGACGACAAGATCCACGCCCGGTCGACCGGCCCCTACTCGATGATCACC
Ae706Ps2_rpoB       GCCCACCTGGTGGACGACAAGATCCACGCCCGGTCGACCGGCCCCTACTCGATGATCACC
Ae150APs1_rpoB      GCGCACCTGGTGGACGACAAGATCCACGCACGGTCGACCGGCCCGTACTCGATGATCACC
Ae168Ps1_rpoB       GCGCACCTGGTGGACGACAAGATCCACGCACGGTCGACCGGCCCGTACTCGATGATCACC
Ae263Ps1_rpoB       GCGCACCTGGTGGACGACAAGATCCACGCACGGTCGACCGGCCCGTACTCGATGATCACC
Ae356Ps1_rpoB       GCGCACCTGGTGGACGACAAGATCCACGCACGGTCGACCGGCCCGTACTCGATGATCACC
Ae707Ps1_rpoB       GCGCACCTGGTGGACGACAAGATCCACGCCCGGTCGACCGGCCCGTACTCGATGATCACC
                    ** ************************** ************** ***************

Ae331Ps2_rpoB       CAGCAGCCGCTGGGCGGTAAGGCGCAGTTCGGTGGCCAGCGCTTCGGCGAGATGGAGTGC
Ae505Ps2_rpoB       CAGCAGCCGCTGGGCGGTAAGGCGCAGTTCGGTGGCCAGCGCTTCGGCGAGATGGAGTGC
Ae406Ps2_rpoB       CAGCAGCCGCTGGGCGGTAAGGCGCAGTTCGGTGGCCAGCGCTTCGGCGAGATGGAGTGC
Ae717Ps2_rpoB       CAGCAGCCGCTGGGCGGTAAGGCGCAGTTCGGTGGCCAGCGCTTCGGCGAGATGGAGTGC
Ae706Ps2_rpoB       CAGCAGCCGCTGGGCGGTAAGGCGCAGTTCGGTGGCCAGCGCTTCGGCGAGATGGAGTGC
Ae150APs1_rpoB      CAGCAGCCGCTGGGCGGTAAGGCGCAGTTCGGTGGTCAGCGCTTCGGTGAGATGGAGTGC
Ae168Ps1_rpoB       CAGCAGCCGCTGGGCGGTAAGGCGCAGTTCGGTGGTCAGCGCTTCGGTGAGATGGAGTGC
Ae263Ps1_rpoB       CAGCAGCCGCTGGGCGGTAAGGCGCAGTTCGGTGGTCAGCGCTTCGGTGAGATGGAGTGC
Ae356Ps1_rpoB       CAGCAGCCGCTGGGCGGTAAGGCGCAGTTCGGTGGTCAGCGCTTCGGTGAGATGGAGTGC
Ae707Ps1_rpoB       CAGCAGCCGCTGGGCGGTAAGGCACAGTTCGGTGGTCAGCGCTTCGGTGAGATGGAGTGC
                    *********************** *********** *********** ************

Ae331Ps2_rpoB       TGGGCGATGCAGGCGTACGGCGCGGCCTACACCCTGCAGGAGCTGCTGACGATCAAGTCC
Ae505Ps2_rpoB       TGGGCGATGCAGGCGTACGGCGCGGCCTACACCCTGCAGGAGCTGCTGACGATCAAGTCC
Ae406Ps2_rpoB       TGGGCGATGCAGGCGTACGGCGCGGCCTACACCCTGCAGGAGCTGCTGACGATCAAGTCC
Ae717Ps2_rpoB       TGGGCGATGCAGGCGTACGGCGCGGCCTACACCCTGCAGGAGCTGCTGACGATCAAGTCC
```

```
Ae706Ps2_rpoB        TGGGCGATGCAGGCGTACGGCGCGGCCTACACCCTGCAGGAGCTGCTGACGATCAAGTCC
Ae150APs1_rpoB       TGGGCGATGCAGGCGTACGGCGCGGCCTACACCCTGCAGGAGCTGCTGACGATCAAGTCC
Ae168Ps1_rpoB        TGGGCGATGCAGGCGTACGGCGCGGCCTACACCCTGCAGGAGCTGCTGACGATCAAGTCC
Ae263Ps1_rpoB        TGGGCGATGCAGGCGTACGGCGCGGCCTACACCCTGCAGGAGCTGCTGACGATCAAGTCC
Ae356Ps1_rpoB        TGGGCGATGCAGGCGTACGGCGCGGCCTACACCCTGCAGGAGCTGCTGACGATCAAGTCC
Ae707Ps1_rpoB        TGGGCGATGCAGGCGTACGGCGCGGCCTACACCCTGCAGGAGCTGCTGACGATCAAGTCC
                     ************************************************************

Ae331Ps2_rpoB        GACGACGTCGTGGGCCGCGTGAAGGTCTACGAGGCGATCGTCAAGGGCGAGAACATCCCG
Ae505Ps2_rpoB        GACGACGTCGTGGGCCGCGTGAAGGTCTACGAGGCGATCGTCAAGGGCGAGAACATCCCG
Ae406Ps2_rpoB        GACGACGTCGTGGGCCGCGTGAAGGTCTACGAGGCGATCGTCAAGGGCGAGAACATCCCG
Ae717Ps2_rpoB        GACGACGTCGTGGGCCGCGTGAAGGTCTACGAGGCGATCGTCAAGGGCGAGAACATCCCG
Ae706Ps2_rpoB        GACGACGTCGTGGGCCGCGTGAAGGTCTACGAGGCGATCGTCAAGGGCGAGAACATCCCG
Ae150APs1_rpoB       GACGACGTCGTGGGCCGCGTGAAGGTCTACGAGGCGATCGTCAAGGGCGAGAACATCCCG
Ae168Ps1_rpoB        GACGACGTCGTGGGCCGCGTGAAGGTCTACGAGGCGATCGTCAAGGGCGAGAACATCCCG
Ae263Ps1_rpoB        GACGACGTCGTGGGCCGCGTGAAGGTCTACGAGGCGATCGTCAAGGGCGAGAACATCCCG
Ae356Ps1_rpoB        GACGACGTCGTGGGCCGCGTGAAGGTCTACGAGGCGATCGTCAAGGGCGAGAACATCCCG
Ae707Ps1_rpoB        GACGACGTCGTGGGCCGCGTGAAGGTCTACGAGGCGATCGTCAAGGGCGAGAACATCCCG
                     ************************************************************

Ae331Ps2_rpoB        GAGCCGGGGATCCCCGAGTCGTTCAAGGTGCTCCTCAAGGAGCTGCAGTCGCTCTGCCTG
Ae505Ps2_rpoB        GAGCCGGGGATCCCCGAGTCGTTCAAGGTGCTCCTCAAGGAGCTGCAGTCGCTCTGCCTG
Ae406Ps2_rpoB        GAGCCGGGGATCCCCGAGTCGTTCAAGGTGCTCCTCAAGGAGCTGCAGTCGCTCTGCCTG
Ae717Ps2_rpoB        GAGCCGGGGATCCCCGAGTCGTTCAAGGTGCTCCTCAAGGAGCTGCAGTCGCTCTGCCTG
Ae706Ps2_rpoB        GAGCCGGGGATCCCCGAGTCGTTCAAGGTGCTCCTCAAGGAGCTGCAGTCGCTCTGCCTG
Ae150APs1_rpoB       GAGCCGGGGATCCCCGAGTCGTTCAAGGTGCTCCTCAAGGAGCTGCAGTCCCTCTGCCTG
Ae168Ps1_rpoB        GAGCCGGGGATCCCCGAGTCGTTCAAGGTGCTCCTCAAGGAGCTGCAGTCCCTCTGCCTG
Ae263Ps1_rpoB        GAGCCGGGGATCCCCGAGTCGTTCAAGGTGCTCCTCAAGGAGCTGCAGTCCCTCTGCCTG
Ae356Ps1_rpoB        GAGCCGGGGATCCCCGAGTCGTTCAAGGTGCTCCTCAAGGAGCTGCAGTCCCTCTGCCTG
Ae707Ps1_rpoB        GAGCCGGGGATCCCCGAGTCGTTCAAGGTGCTCCTCAAGGAGCTGCAGTCCCTCTGCCTG
                     ************************************************** *********

Ae331Ps2_rpoB        AACGTCGAGGTGCTCTCCAGCGACGGTGCGGCGATCGAGATGCGGGACGCCGACGACGAA
Ae505Ps2_rpoB        AACGTCGAGGTGCTCTCCAGCGACGGTGCGGCGATCGAGATGCGGGACGCCGACGACGAA
Ae406Ps2_rpoB        AACGTCGAGGTGCTCTCCAGCGACGGTGCGGCGATCGAGATGCGGGACGCCGACGACGAA
Ae717Ps2_rpoB        AACGTCGAGGTGCTCTCCAGCGACGGTGCGGCGATCGAGATGCGGGACGCCGACGACGAA
Ae706Ps2_rpoB        AACGTCGAGGTGCTCTCCAGCGACGGTGCGGCGATCGAGATGCGGGACGCCGACGACGAA
Ae150APs1_rpoB       AACGTCGAGGTGCTCTCCAGCGACGGCGCGGCGATCGAGATGCGGGACGCCGACGACGAG
Ae168Ps1_rpoB        AACGTCGAGGTGCTCTCCAGCGACGGCGCGGCGATCGAGATGCGGGACGCCGACGACGAG
Ae263Ps1_rpoB        AACGTCGAGGTGCTCTCCAGCGACGGCGCGGCGATCGAGATGCGGGACGCCGACGACGAG
Ae356Ps1_rpoB        AACGTCGAGGTGCTCTCCAGCGACGGCGCGGCGATCGAGATGCGGGACGCCGACGACGAG
Ae707Ps1_rpoB        AACGTCGAGGTGCTCTCCAGCGACGGCGCGGCGATCGAGATGCGGGACGCCGACGACGAG
                     ************************** ********************************* 

Ae331Ps2_rpoB        GACCTCGAGCGTGCCGCGGCGAATCTGGGCATCAACCTGTCCAGCCGCCCGGGCTCGGAG
Ae505Ps2_rpoB        GACCTCGAGCGTGCCGCGGCGAATCTGGGCATCAACCTGTCCAGCCGCCCGGGCTCGGAG
Ae406Ps2_rpoB        GACCTCGAGCGTGCCGCGGCGAATCTGGGCATCAACCTGTCCAGCCGCCCGGGCTCGGAG
Ae717Ps2_rpoB        GACCTCGAGCGTGCCGCGGCGAATCTGGGCATCAACCTGTCCAGCCGCCCGGGCTCGGAG
Ae706Ps2_rpoB        GACCTCGAGCGTGCCGCGGCGAATCTGGGCATCAACCTGTCCAGCCGCCCGGGCTCGGAG
Ae150APs1_rpoB       GACCTCGAGCGTGCCGCCGCGAACCTGGGCATCAACCTGTCCAGCCGCCCGGGCTCGGAG
Ae168Ps1_rpoB        GACCTCGAGCGTGCCGCCGCGAACCTGGGCATCAACCTGTCCAGCCGCCCGGGCTCGGAG
Ae263Ps1_rpoB        GACCTCGAGCGTGCCGCCGCGAACCTGGGCATCAACCTGTCCAGCCGCCCGGGCTCGGAG
Ae356Ps1_rpoB        GACCTCGAGCGTGCCGCCGCGAACCTGGGCATCAACCTGTCCAGCCGCCCGGGCTCGGAG
Ae707Ps1_rpoB        GACCTCGAGCGTGCCGCCGCGAACCTGGGCATCAACCTGTCCAGCCGCCCGGGCTCGGAG
                     ***************** ***** ************************************

Ae331Ps2_rpoB        TCGATGACCGTCGACGACGTCGTCAACTGA
Ae505Ps2_rpoB        TCGATGACCGTCGACGACGTCGTCAACTGA
Ae406Ps2_rpoB        TCGATGACCGTCGACGACGTCGTCAACTGA
Ae717Ps2_rpoB        TCGATGACCGTCGACGACGTCGTCAACTGA
Ae706Ps2_rpoB        TCGATGACCGTCGACGACGTCGTCAACTGA
Ae150APs1_rpoB       TCGATGACCGTCGACGACGTCGTCAACTGA
Ae168Ps1_rpoB        TCGATGACCGTCGACGACGTCGTCAACTGA
Ae263Ps1_rpoB        TCGATGACCGTCGACGACGTCGTCAACTGA
Ae356Ps1_rpoB        TCGATGACCGTCGACGACGTCGTCAACTGA
Ae707Ps1_rpoB        TCGATGACCGTCGACGACGTCGTCAACTGA
                     ******************************
```

Supplementary Table 4. *rpoB* gene percentage identity across *Pseudonocardia* strains.

	**Ae150A_Ps1_*rpoB***	**Ae168_Ps1_*rpoB***	**Ae263_Ps1_*rpoB***	**Ae356_Ps1_*rpoB***	**Ae707_Ps1_*rpoB***	**Ae331_Ps2_*rpoB***	**Ae406_Ps2_*rpoB***	**Ae505_Ps2_*rpoB***	**Ae706_Ps2_*rpoB***	**Ae717_Ps2_*rpoB***
**Ae150A_Ps1_*rpoB***	100	100	100	100	99.74	95.01	95.01	94.96	94.99	95.01
**Ae168Ps1_*rpoB***	100	100	100	100	99.74	95.01	95.01	94.96	94.99	95.01
**Ae263_Ps1_*rpoB***	100	100	100	100	99.74	95.01	95.01	94.96	94.99	95.01
**Ae356Ps1_*rpoB***	100	100	100	100	99.74	95.01	95.01	94.96	94.99	95.01
**Ae707_Ps1_*rpoB***	99.74	99.74	99.74	99.74	100	95.1	95.1	95.04	95.07	95.1
**Ae331_Ps2_*rpoB***	95.01	95.01	95.01	95.01	95.1	100	100	99.94	99.97	100
**Ae406_Ps2_*rpoB***	95.01	95.01	95.01	95.01	95.1	100	100	99.94	99.97	100
**Ae505_Ps2_*rpoB***	94.96	94.96	94.96	94.96	95.04	99.94	99.94	100	99.91	99.94
**Ae706_Ps2_*rpoB***	94.99	94.99	94.99	94.99	95.07	99.97	99.97	99.91	100	99.97
**Ae717_Ps2_*rpoB***	95.01	95.01	95.01	95.01	95.1	100	100	99.94	99.97	100

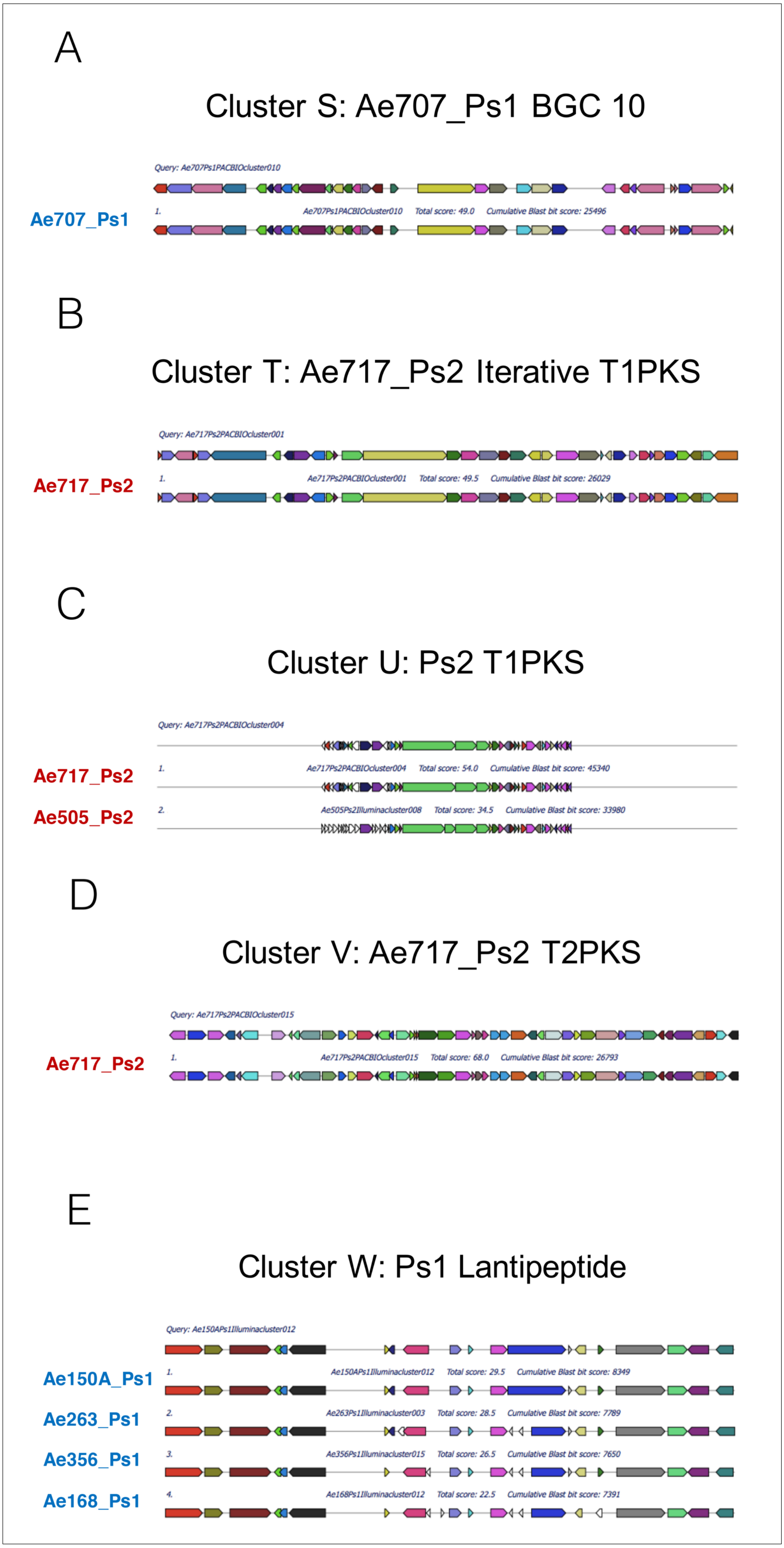

A
Cluster S: Ae707_Ps1 BGC 10
Query: Ae707Ps1PACBIOcluster010
Ae707_Ps1
1. Ae707Ps1PACBIOcluster010 Total score: 49.0 Cumulative Blast bit score: 25496
B
Cluster T: Ae717_Ps2 Iterative T1PKS
Query: Ae717Ps2PACBIOcluster001
Ae717_Ps2
1. Ae717Ps2PACBIOcluster001 Total score: 49.5 Cumulative Blast bit score: 26029
C
Cluster U: Ps2 T1PKS
Query: Ae717Ps2PACBIOcluster004
Ae717_Ps2
1. Ae717Ps2PACBIOcluster004 Total score: 54.0 Cumulative Blast bit score: 45340
Ae505_Ps2
2. Ae505Ps2Illuminacluster008 Total score: 34.5 Cumulative Blast bit score: 33980
D
Cluster V: Ae717_Ps2 T2PKS
Query: Ae717Ps2PACBIOcluster015
Ae717_Ps2
1. Ae717Ps2PACBIOcluster015 Total score: 68.0 Cumulative Blast bit score: 26793
E
Cluster W: Ps1 Lantipeptide
Query: Ae150APs1Illuminacluster012
Ae150A_Ps1
1. Ae150APs1Illuminacluster012 Total score: 29.5 Cumulative Blast bit score: 8349
Ae263_Ps1
2. Ae263Ps1Illuminacluster003 Total score: 28.5 Cumulative Blast bit score: 7789
Ae356_Ps1
3. Ae356Ps1Illuminacluster015 Total score: 26.5 Cumulative Blast bit score: 7650
Ae168_Ps1
4. Ae168Ps1Illuminacluster012 Total score: 22.5 Cumulative Blast bit score: 7391

Supplementary Figure 11. Multigene blast outputs of other biosynthetic gene clusters that are not shared amongst or between Ps phylotypes. Unique clusters include; cluster S: Ae707_Ps1 encodes a unique cluster forming an unknown product **(A)**, cluster T: Ae717_Ps2 encodes an iterative PKS cluster not found in other Ps strains **(B)**, cluster U: Ae717_Ps2 and Ae505_Ps2 are the only strains to have an additional T1PKS cluster **(C)**, cluster V: Ae717_Ps2 encodes a T2PKS cluster not found in other Ps strains **(D)**, cluster W: a subset of Ps1 strains encode a cluster for production of a lantipeptide **(E)**.

Supplementary Table 5: tBlastn of *Pseudonocardia autotrophica* polyene NPP BGC amino acid sequences against putative nystatin clusters of Ae707_Ps1 (PacBio) and Ae717_Ps2 (PacBio). NppC against Ae707_Ps1 nystatin cluster has two results as the blast algorithm separated the sequences into two. All PKS type I proteins against Ae706_Ps2 were given the 3 top hits, as the conservation is low it is difficult to assign identity of the PKS proteins to the respective homologues present in the *P. autotrophica* cluster.

NPP BGC	Amino Acid Length	Protein Function	Ae707_Ps1 Cluster % Identity	Ae707_Ps1 Query % Coverage	Ae717_Ps2 Cluster % Identity	Ae717_Ps2 Query % Coverage
NppY	475	Glycosyltransferase	83	98	43	94
NppF	224	Phospho-pantetheinyl transferase	90	99	70	0.045
NppG	592	ABC transporter	90	99	56	86
NppH	584	ABC transporter	89	100	52	97
NppDIII	344	GDP-mannose-4,6-dehydratase	97	100	79	100
NppI	9499	Type I PKS	90	100	59<br>49<br>62	26<br>32<br>15
NppJ	5425	Type I PKS	86	100	57<br>59<br>53	35<br>23<br>34
NppK	2031	Type I PKS	89	100	60<br>59<br>60	55<br>49<br>50
NppL	403	P450 mono oxygenase	85	97	47	90
NppN	413	P450 mono oxygenase	84	100	69	90
NppDII	352	Aminotransferase	96	100	74	99
NppDI	489	Glycosyltransferase	93	97	60	96
NppA	1097	Type I PKS	89	100	59<br>57<br>60	79<br>79<br>78
NppB	3175	Type I PKS	90	100	54<br>48	78<br>47

					48	46
NppC	10876	Type I PKS	87<br>89	56<br>43	54<br>52<br>53	43<br>37<br>32
NppE	274	Thioesterase	91	100	53	82
NppRI	946	Regulation	86	100	47	97
NppRII	765	Regulation	79	100	45	93
NppRIII	925	Regulation	77	97	43	62
NppO	526	Decarboxylase	97	100	87	98
NppRIV	213	Regulation	94	100	68	91
NppM	138	Ferredoxin	74	90	55	69
NppRV	963	Regulation	69	97	40	95
NppRVI	684	Regulation	70	97	38	97

Supplementary Table 6. Blastx of the PKS amino acid sequences from either the Ae707_Ps1 PacBio nystatin cluster against Ps1 strains nucleotide sequence or the PKS amino acid sequences from the Ae717_Ps2 PacBio nystatin cluster against Ps2 strains nucleotide sequence. (*) The PKS modules in PKS amino acid sequences for 3119 do not cover the full amino acid length in any of the other Ps1 strains and this corresponds to an amino acid sequence that encodes two modules.

**Amino Acid**	**Length (aa)**	**Ae150A_ Ps1 Illumina Identity**	**Ae150A_ Ps1 Illumina Locus**	**Ae168_Ps 1 Illumina Identity**	**Ae168_Ps 1 Illumina Locus**	**Ae263_Ps 1 Illumina Identity**	**Ae263_Ps 1 Illumina Locus**	**Ae356_Ps 1 Illumina Identity**	**Ae356_Ps 1 Illumina Locus**	**Ae707_Ps 1 PacBio Identity**	**Ae707_Ps 1 PacBio Locus**
Ae707_Ps1 _3121 NypA/Npp A	1106	565/603 (94%)<br>391/431 (91%)	43301-45049<br>42105-43289	565/603 (94%)<br>391/431 (91%)	47494-49242<br>46298-47482	565/603 (94%)<br>391/431 (91%)	5432888-5431140<br>5434084-5432900	565/603 (94%)<br>391/431 (91%)	1678228-1679976<br>1677032-1678216	1106/1106 (100%)	3411435-3408118
Ae707_Ps1 _3120 NypB/Npp B	3188	1530/1555 (98%)<br>832/843 (99%)<br>750/767 (98%)	50138-54793<br>45302-47830<br>47842-50133	1530/1555 (98%)<br>832/843 (99%)<br>750/767 (98%)	54331-58986<br>49495-52023<br>52035-54326	1530/1555 (98%)<br>832/843 (99%)<br>750/767 (98%)	5426051-5421396<br>5430887-5428359<br>5428347-5426056	1530/1555 (98%)<br>832/843 (99%)<br>750/767 (98%)	1685065-1689720<br>1680409-1682757<br>1682769-1685060	3188/3188 (100%)	3408069-3398506
Ae707_Ps1 _3119 NypC/Npp C *	10899	3683/4065 (91%)<br>1826/1857 (98%)<br>1118/1139 (98%)	6846-76113<br>54817-60387<br>60389-63805	3685/4065 (91%)<br>1826/1857 (98%)<br>1118/1139 (98%)	68136-80306<br>59010-64580<br>64582-67998	3685/4065 (91%)<br>1825/1857 (98%)<br>1118/1139 (98%)	5412246-5400076<br>5421192-5415802<br>5415800-5412384	3685/4065 (91%)<br>1826/1857 (98%)<br>1118/1139 (98%)	1698870-1711040<br>1689744-1695314<br>1695316-1698732	10895/10895 (100%)	3398482-3365798
Ae707_Ps1 _3128 NypI/NppI	9524	6924/7034 (98%)<br>1448/1470 (99%)<br>994/1007 (99%)	760991-739890<br>739869-735466<br>764056-761042	6924/7034 (98%)<br>1448/1470 (99%)<br>994/1007 (99%)	760457-739356<br>739335-734932<br>763522-760508	6925/7034 (98%)<br>1448/1470 (99%)<br>994/1007 (99%)	4718060-4739161<br>4739182-4743585<br>4714995-4718009	6925/7034 (98%)<br>1448/1470 (99%)<br>994/1007 (99%)	2393068-2371967<br>2371946-2367543<br>2396133-2393119	9524/9524 (100%)	3468317-3439746
Ae707_Ps1 _3127 NypJ/NppJ	5420	3079/3163 (97%)<br>2178/2248 (97%)	735442-726068<br>726011-719274	3079/3163 (97%)<br>2178/2248 (97%)	734908-725534<br>725477-718740	3079/3163 (97%)<br>2178/2248 (97%)	4743609-4752983<br>4753040-47597777	3079/3163 (97%)<br>2178/2248 (97%)	2367519-2358145<br>2358088-2351351	5420/5420 (100%)	3439722-3423463
Ae707_Ps1 _3126 NypK/Npp K	2027	969/981 (99%)<br>1003/1007 (99%)	719233-716291<br>716166-713146	969/981 (99%)<br>1002/1007 (99%)	718699-715757<br>715632-712612	969/981 (99%)<br>1001/1006 (99%)	4759818-4762760<br>4762889-4765906	969/981 (99%)<br>1001/1006 (99%)	2351310-2348368<br>2348239-2345222	2025/2025 (100%)	3423422-3417348
**Amino Acid**	**Length (aa)**	**Ae331_Ps 2 Illumina Identity**	**Ae331_Ps 2 Illumina Locus**	**Ae406_Ps 2 Illumina Identity**	**Ae406_Ps 2 Illumina Locus**	**Ae505_Ps 2 Illumina Identity**	**Ae505_Ps 2 Illumina Similarity**	**Ae706_Ps 2 Illumina Identity**	**Ae706_Ps 2 Illumina Similarity**	**Ae717_Ps 2 PacBio Identity**	**Ae717_Ps 2 PacBio Similarity**
Ae717_Ps2 _3010 PKS A	2554	2540/2540 (100%)	4471485-4479104	2540/2540 (100%)	1602520-1594901	2539/2540 (99%)	5280963-5273347	2540/2540 (100%)	1611426-1603807	2540/2540 (100%)	3308031-3300412
Ae717_Ps2 _3009 PKS B	6856	6838/6859 (99%)	4479198-4499774	6838/6865 (99%)	1594807-1574213	6192/6239 (99%)<br>613/632 (97%)	5271344-5252703<br>5273253-5271358	6838/6859 (99%)	1603713-1583137	6856/6856 (100%)	3300318-3279751
Ae717_Ps2 _3026 PKS C	3490	3344/3351 (99%)	4400747-4410799	3344/3351 (99%)	1668401-1658349	-	-	3344/3351 (99%)	1682164-1672112	3361/3361 (100%)	3377832-3367750
Ae717_Ps2 _3025 PKS D	5917	5878/5917 (99%)	4411202-4428913	5879/5917 (99%)	1657946-1640235	-	-	5879/5917 (99%)	1671709-1653998	5917/5917 (100%)	3367381-3349631
Ae717_Ps2 _3024 PKS E	3234	3074/3078 (99%)<br>155/155 (100%)	4428930-4438163<br>4438177-4438641	3074/3078 (99%)<br>155/155 (100%)	1640218-1630985<br>1630971-1630507	-	-	3074/3078 (99%)<br>155/155 (100%)	1653981-1644748<br>1644734-1644270	3234/3234 (100%)	3349614-3339913
Ae717_Ps2 _3023 PKS F	3747	3740/3747 (99%)	4438641-4449881	3740/3747 (99%)	1630507-1619267	-	-	3739/3747 (99%)	1644270-1633030	3747/3747 (100%)	3339913-3328673
Ae717_Ps2 _3022 PKS G	2048	2047/2048 (99%)	4449921-4456064	2047/2048 (99%)	1619227-1613084	-	-	2047/2048 (99%)	1632990-1626847	2048/2048 (100%)	3328633-3322490

References

Larkin, M. A., Blackshields, G., Brown, N. P., Chenna, R., McGettigan, P. A., McWilliam, H., et al. (2007). Clustal W and Clustal X version 2.0. *Bioinformatics* 23, 2947–2948. doi:10.1093/bioinformatics/btm404.

Weber, T., Blin, K., Duddela, S., Krug, D., Kim, H. U., Bruccoleri, R., et al. (2015). antiSMASH 3.0-a comprehensive resource for the genome mining of biosynthetic gene clusters. *Nucleic Acids Res* 43, W237–43. doi:10.1093/nar/gkv437.
